# Characterization of novel pollen-expressed transcripts reveals their potential roles in pollen heat stress response in *Arabidopsis thaliana*

**DOI:** 10.1007/s00497-020-00400-1

**Published:** 2021-01-18

**Authors:** Nicholas Rutley, Laetitia Poidevin, Tirza Doniger, Richard L. Tillett, Abhishek Rath, Javier Forment, Gilad Luria, Karen A. Schlauch, Alejandro Ferrando, Jeffery F. Harper, Gad Miller

**Affiliations:** 1 The Mina and Everard Goodman Faculty of Life Sciences, Bar Ilan University, 5290002 Ramat-Gan, Israel; 2 Instituto de Biología Molecular y Celular de Plantas, Consejo Superior de Investigaciones Cient́́if́icas-Universitat Politècnica de València, Valencia, Spain; 3 Department of Biochemistry and Molecular Biology, University of Nevada at Reno, Reno, NV 89557 USA; 4 Nevada INBRE Bioinformatics Core, University of Nevada at Reno, Reno, NV 89557 USA; 5 Institute of Health Innovation, Desert Research Institute, Department of Pharmacology, University of Nevada at Reno, Reno, NV 89557 USA

**Keywords:** Heat, Transcriptome, Pollen, Long noncoding RNA, Translatome, *Arabidopsis*

## Abstract

**Key message:**

*Arabidopsis* pollen transcriptome analysis revealed new intergenic transcripts of unknown function, many of which are long non-coding RNAs, that may function in pollen-specific processes, including the heat stress response.

**Abstract:**

The male gametophyte is the most heat sensitive of all plant tissues. In recent years, long noncoding RNAs (lncRNAs) have emerged as important components of cellular regulatory networks involved in most biological processes, including response to stress. While examining RNAseq datasets of developing and germinating *Arabidopsis thaliana* pollen exposed to heat stress (HS), we identified 66 novel and 246 recently annotated intergenic expressed loci (XLOCs) of unknown function, with the majority encoding lncRNAs. Comparison with HS in cauline leaves and other RNAseq experiments indicated that 74% of the 312 XLOCs are pollen-specific, and at least 42% are HS-responsive. Phylogenetic analysis revealed that 96% of the genes evolved recently in *Brassicaceae*. We found that 50 genes are putative targets of microRNAs and that 30% of the XLOCs contain small open reading frames (ORFs) with homology to protein sequences. Finally, RNAseq of ribosome-protected RNA fragments together with predictions of periodic footprint of the ribosome P-sites indicated that 23 of these ORFs are likely to be translated. Our findings indicate that many of the 312 unknown genes might be functional and play a significant role in pollen biology, including the HS response.

**Electronic supplementary material:**

The online version of this article (10.1007/s00497-020-00400-1) contains supplementary material, which is available to authorized users.

## Introduction

High temperatures have profound harmful effects on plants’ reproduction, often causing severe damages to complete loss of crops (Slattery and Ort 2019; Jacott and Boden 2020; Lohani et al. 2020). The male gametophyte is thought to be the most sensitive to heat stress (HS) compared to all other organs and tissues in most plants, including the female gametophyte (Zinn et al. 2010; Muller and Rieu 2016; Rieu et al. 2017). The male gametophyte exists as a short cell lineage, beginning with the completion of the pollen mother cell meiosis within the anther, producing four haploid microspores. These microspores undergo an asymmetric cell division (pollen mitosis I) to produce a vegetative cell and generative cell. The generative cell divides once more (pollen mitosis II), giving rise to two sperm cells, followed by a maturation stage of the pollen in preparation for dehiscence. At pollination, pollen lands on a receptive stigma and then grows a tube that is guided toward the ovule. In species that underwent one round of pollen mitosis in the anther, the mitotic division of the generative cell takes place during pollen tube growth. Upon arrival at the ovule, the sperm cells are released from the pollen tube and go on to fertilize the egg and central cell (Suzuki 2009). These delicate stages of pollen developmental are extremely vulnerable to HS, with even a short moderate or mild chronic heat stress causing an increase in pollen abortion, reduced fitness, tube growth arrest, or even tube rupture (Mesihovic et al. 2016; Luria et al. 2019). Also, the expression of many heat shock proteins (HSPs) and heat shock transcription factors (HSFs) is poor in pollen compared to other plant cells (Mascarenhas and Crone 1996; Muller and Rieu 2016). These findings have contributed to the perception that pollen lacks a robust HS response (HSR).

RNAseq experiments in pollen from different species indicated that HS has a profound impact on pollen gene expression and suggested that pollen may have different HSRs than other types of plant cells (Fragkostefanakis et al. 2016; Muller and Rieu 2016; Begcy et al. 2019). The first RNAseq reports in *Arabidopsis* and maize mature pollen identified a significant number of novel transcribed loci, including transcripts with homologies to known proteins and long noncoding RNAs (lncRNAs) (Loraine et al. 2013; Chettoor et al. 2014). Interestingly, the reproductive tissues of maize, including pollen and embryo sac, had more examples of lncRNA expression than any other tissues characterized (Chettoor et al. 2014). LncRNAs are transcripts exceeding 200 nucleotides in length that lack open reading frames longer than 100 amino acids. This somewhat arbitrary limit distinguishes lncRNAs from small noncoding RNAs such as microRNAs (miRNAs), small interfering RNAs (siRNAs), small nucleolar RNAs (snoRNAs), and other short RNAs (Ma et al. 2013). LncRNAs are generally polyadenylated and often have highly tissue-specific expression (Yu et al. 2019). LncRNAs mainly include intergenic ncRNAs (lincRNAs), intronic ncRNAs (incRNAs), and natural antisense transcripts (NATs) that overlap with coding regions. They are associated with a broad range of biological processes, including plant development and stress response, influencing gene expression by acting as molecular scaffolds, decoys or target mimics of microRNAs (miRNAs), and small interfering RNA precursors (Liu et al. 2012; Yu et al. 2019). As molecular scaffolds, lncRNAs may bind both DNA and protein recruiting regulatory components such as chromatin modulators to specific gene loci. As decoys, some lncRNAs may bind transcription factors to prevent them from interacting with DNA to induce target gene expression. Functional analyses of several plant lncRNAs demonstrated their profound involvement in plant development and physiology, including that of the male gametophyte (Liu et al. 2012; Yu et al. 2019). For example, a single polymorphism change in the sequence of long-day-specific male-fertility–associated RNA (LDMAR) in rice alters the secondary structure of the RNA molecule and its ability to function, causing defective anthers and pollen grains resulting in male sterility (Ding et al. 2012).

Many lncRNAs contain small open reading frames (smORFs) that can be translated into small polypeptides (> 100 amino acids), also known as micropeptides or microproteins, with growing evidence supporting that they are biologically functional, regulating target genes in *cis* or *trans* (Liu et al. 2012; Plaza et al. 2017). The pollen-specific Zm908 lncRNA gene in maize encodes microproteins that interact with profilin 1, sequestering it from binding actin filaments. Overexpression of Zm908 caused developmental defects in maize pollen development and reduced germination (Dong et al. 2013). The potential involvement of lncRNAs in cytoplasmic male sterility (CMS) has been recently suggested; however, conclusive evidence directly implicating lncRNAs with CMS is still missing (Mishra and Bohra 2018). An extensive data survey of RNAseq experiments in several plants, including wheat, maize, rice, and *Arabidopsis*, showed that a relatively large proportion of lncRNAs are responsive to abiotic stresses (Di et al. 2014; Yuan et al. 2018; Lv et al. 2019). The expression of HSFB2a, which is essential for the fertility of both the female and male gametophytes in *Arabidopsis*, is controlled by heat stress-induced NAT (Wunderlich et al. 2014). The study of lncRNAs in plants is still an emerging area of research with the function of most lncRNAs awaiting discovery (Yu et al. 2019). Thus, research is needed to identify additional functional examples of lncRNAs involved in the HSR in both vegetative tissues and male gametophyte.

The latest complete *Arabidopsis thaliana* reference genome annotation, Araport11v4, significantly expanded the number of genes compared with the earlier TAIR10 annotation, including a massive increase in the number of long noncoding RNAs (lincRNAs and NATs) from 259 to 3559 genes. The updated annotation version also included 508 intergenic novel transcribed regions (Cheng et al. 2017; Krishnakumar et al. 2017) and resulted from an assembly of tissue-specific RNAseq libraries from 113 datasets, which also included pollen. However, the transcriptome coverage and number of expressed genes in these pollen datasets were relatively small, including only 6301 pollen-expressed genes (Cheng et al. 2017).

In this study, we report on the identification of 312 uncharacterized polyadenylated expressed loci (XLOCs) from three independent RNAseq experiments with *Arabidopsis* pollen exposed to HS. Sixty-six of those pollen XLOCs are entirely novel. Ninety-two percent of the 312 XLOCs encode for lncRNAs, and 73% were not present in other sporophytic tissues RNAseq datasets, suggesting that they are pollen-specific. Also, most of the XLOCs show differential expression in pollen matured under HS. Phylogenetic classification revealed that more than half of these genes are *A. thaliana*-specific, but nine are highly conserved in eudicots. We identified 50 of the XLOCs as putative targets of microRNAs, suggesting potential involvement in gene expression regulation. Many of the identified lncRNAs contain smORFs for potential microproteins with homology to protein sequences. Lastly, ribosome elongating footprint analysis of ribosome-protected RNAseq data showed that 23 of the ORF-containing transcripts are very likely to be translated. Our findings support the perception that pollen has a unique HSR and provide multiple targets for functional analyses of new pollen-specific genes with the potential of profoundly impacting pollen development and physiology.

## Materials and methods

### Plant material, growth conditions, and pollen collection

*Arabidopsis thaliana* (Col-0) were grown in 16:8 light regime and at 21 °C for 5 weeks until flowering had established. Pollen was harvested from open flowers using a customized pollen vacuum wand (Johnson-Brousseau and McCormick 2004). The customized pollen vacuum wand consisted of nylon mesh filters (Membrane Solutions, USA) of three grades: 80 µm, 40 µm, and 10 µm. Pollen deposited on the 10-micron filter was washed off using 0.3 M mannitol, pelleted, frozen in liquid nitrogen, and stored at − 80 °C until required. After vacuuming, plants were then moved to a heat stress regime during the light photoperiod, consisting of a stepwise increase in temperature from the beginning of the light period from 22 °C to 38 °C over 6 h, holding at 38 °C for 2 h, then a decrease to 22 °C overnight. Plants were maintained at 22 °C during the night (Figure S1). This heat stress regime was repeated for 3 days, and pollen was collected on the morning of the fourth day. In parallel with pollen sampling, cauline leaves representing control and heat stress conditions were collected from the same plants. Cauline leaf control samples were collected on the same day as pollen control samples, while heat-stressed cauline leaf samples were collected at the heat stress maximum (38 °C) on the third day of heat stress. Three independent biological replicates were collected for each sample type and growth condition.

### RNA isolation, library preparation, and sequencing

For RNA sequencing, total RNA was extracted from pollen and cauline leaf samples using RNA were extracted using the TRizolTM reagent (Life Technologies, Carlsbad, CA) according to the manufacturer’s recommendations. RNA was shipped to Beijing Genomics Institute (BGI, China) for multiplexed Illumina HiSeq 2000 paired-end sequencing. Sequencing was performed at 2 × 50 bp for all samples.

### Sequence quality control (QC) and novel gene prediction

Sequence pairs were trimmed and filtered using Trimmomatic v. 032 (Bolger et al. 2014). After filtering sequences to remove multiplexing barcodes and sequencing adapters using NGS QC Toolkit (v.2.3), sequence pairs were aligned to TAIR10 *A. thaliana* reference genome using the spliced aligner tool Tophat2 (V.2.0.13) and Bowtie 2 (v.2.2.4).

Unannotated transcripts (i.e., transcripts not mapped to the TAIR10 reference transcriptome) were identified and mapped using the in-house services of the Nevada INBRE Bioinformatics Core at the University of Reno (USA). Briefly, genome alignments generated by Tophat2 were processed using the Cufflinks package (v.2.2.1) reference annotation-based transcript (RABT) assembly method. Predictions from the independent Cufflinks runs were combined using the cuffmerge command to produce a single set of predicted transcripts. To determine which of the unannotated transcripts were annotated in the Araport11 genome annotation, we used IntersectBed from the Bedtools suite (Quinlan and Hall 2010) to compare Araport11 with our novel transcripts. Those transcripts that overlapped with an Araport11 annotated gene were considered annotated.

### Gene quantification

Generating sequence alignments: Sequence pairs were aligned to the *A. thaliana* TAIR10 genome using HISAT spliced read alignment tool (v.0.1.6; (Kim et al. 2015)). Genomic coordinates of the putative novel transcripts were combined with the exon coordinates of all known TAIR10 genes (Swarbreck et al. 2008), as annotated in Ensembl build 27 into a Gene Transfer Format (GTF) file and together supplied to the HISAT aligner via the included extract_splice_sites.py tool and ‘-known-splice-site-infile’ option, with all other options set as default. Novel genes were assigned intergenic XLOCs identifiers. For expression quantification, the number of read pairs aligned to each gene was counted using the featureCounts tool from the subread package (v. 0.30; (Liao et al. 2014)). Read pairs were counted just once per pair, summarized to gene loci. Ambiguous or multiply-aligned read pairs were excluded from count totals. These raw read counts were used as input for DESeq2 (v1.18.1) (Love et al. 2014). Reads were then normalized to Reads Per Million reads (RPM).

### GP_HS data analysis

The bioinformatics protocols for the GP_HS libraries followed the same procedures as described (Poidevin et al. 2020) with a few changes. First, during the cleaning steps the noncoding RNAs were not bioinformatically removed. The GTF file used for the htseq-count included the identified XLOCs both in forward and in reverse orientation. Finally, the htseq-count used the ‘nonunique all’ function to attribute a read to both RNAs when the mapping overlapped two RNAs.

### RNA-seq data analysis

Transcript counts were filtered to exclude those with < 10 counts in all samples. Filtered count data were then normalized via the median ratio method (Anders and Huber 2010). Differential gene expression between control and HS conditions was examined using DESeq2 (Love et al. 2014). Comparisons were considered using simple contrasts. Fold changes in gene expression were transformed to log_2_ fold change values. Correction for multiple testing was performed within each comparison to adjust for the false discovery rate. XLOCs with ≥ 1 or ≤  − 1 log_2_ fold changes and adjusted *p* value < 0.05 were considered to meet the standard significance threshold for this study. Principal component analysis (PCA) was performed using DESeq2 with the variance-stabilized normalized RNA-Seq data to validate the clear separation between the different conditions.

### Validation of RNA-Seq data by real-time quantitative PCR (qRT-PCR)

For validation of gene expression using qRT-PCR: Total RNA was extracted from Col-0 wild-type control inflorescences, following the heat stress regime using Trizol reagent (Thermo Fisher Scientific). First-strand cDNA synthesis was performed using qScript Flex cDNA synthesis kit (Quantabio). qRT-PCR was performed in CFX96Connect (Bio-Rad). The gene HTR5 (AT4G40040) served as a reference gene. Relative normalized expressed was calculated using 2−^ΔΔCq^ method. Primer sequences are listed in Table S1.

### Phylostratigraphic analysis

To understand the conservation of these novel transcripts, we performed megablast at the NCBI using the novel transcripts as the query and requiring an *E* value of <  = 1e − 05. We ran the search twice using the nr/nt database once excluding hits within *Brassicaceae* and second time only searching within *Brassicaceae* but eliminating hits to *Arabidopsis thaliana*.

We then extracted the full taxonomic lineage for each hit and were then able to assign hits to a given level in the phylogenetic tree.

### Other bioinformatics tools used

CANTATAdb (http://cantata.amu.edu.pl/) was used to identify conserved lncRNAs (Szczesniak et al. 2016). psRNATarget V2 (2017 release) (http://plantgrn.noble.org/psRNATarget/) (Dai and Zhao 2011) was used to identify putative miRNA targets among novel genes. Hits were filtered using an expectation threshold of ≤ 3. IVG (v.2.4.3.) was used to visualize mapped reads to the *Arabidopsis* genome, importing Araport11.bed files.

## Results

### Mapping and identification of novel pollen-expressed transcripts

**Fig. 1:**
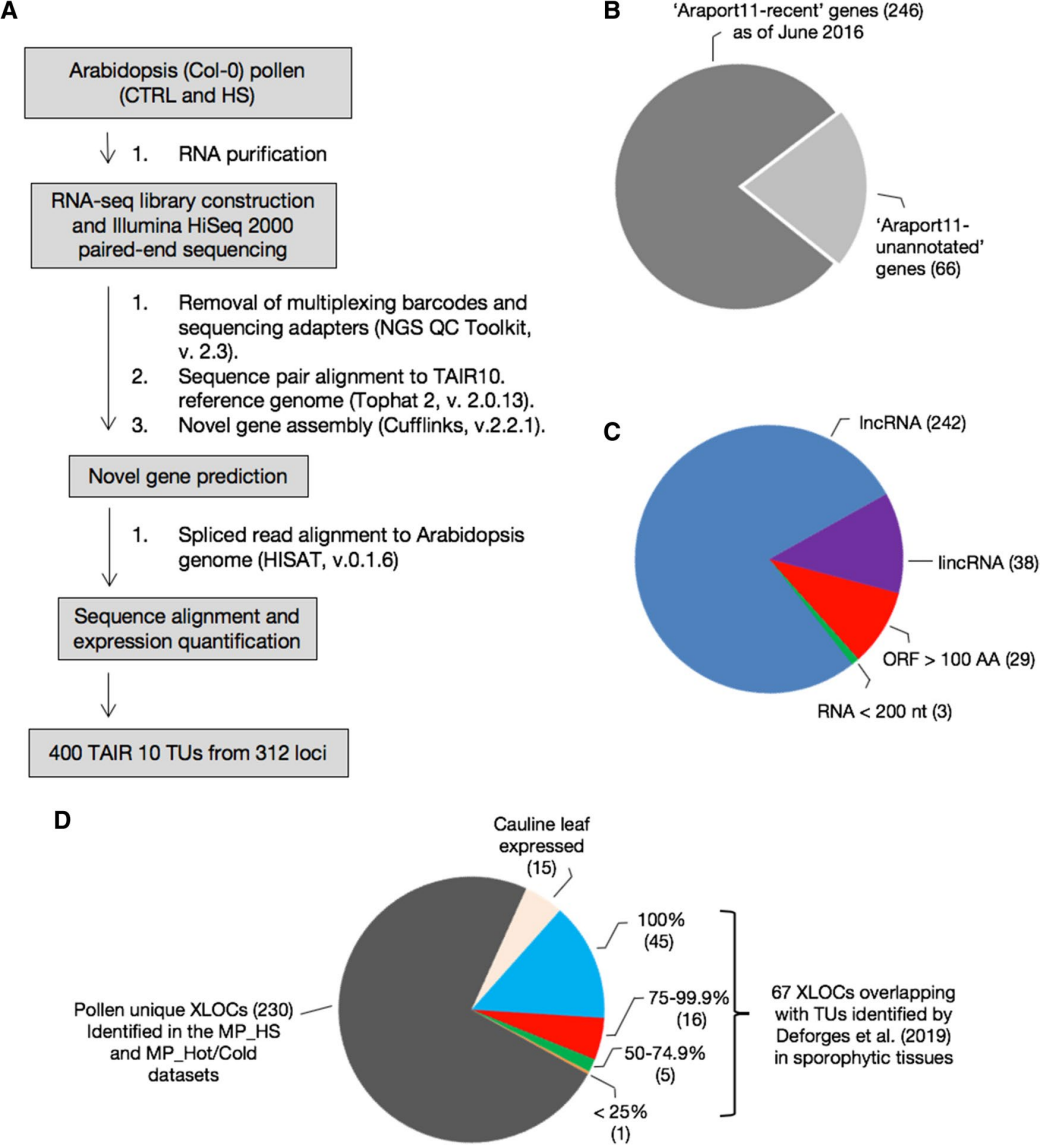
Identification and characterization of novel transcriptional units (TUs). **a** The workflow pipeline for the identification of TUs in MP_HS and MP_Hot/Cold RNA-seq data. **b** Pie chart of the distribution between expressed loci (XLOCs) annotated at the previous update of Araport11 (‘Araport-recent’) and XLOCs that remained as unannotated Araport11 genome annotation (v1.10.4, release 06/2016). **c** Breakdown of XLOCs by class: long noncoding RNA (lncRNA), long intergenic noncoding RNA (lincRNA), open reading frame (ORF) > 100 amino acids, and RNA length < 200 nucleotides. **d** 230 TUs identified in pollen libraries from MP_HS and MP_Hot/Cold that were not found among MP_HS cauline leaf libraries or sporophytic lncRNAs from Deforges et al. (2019)

**Fig. 2:**
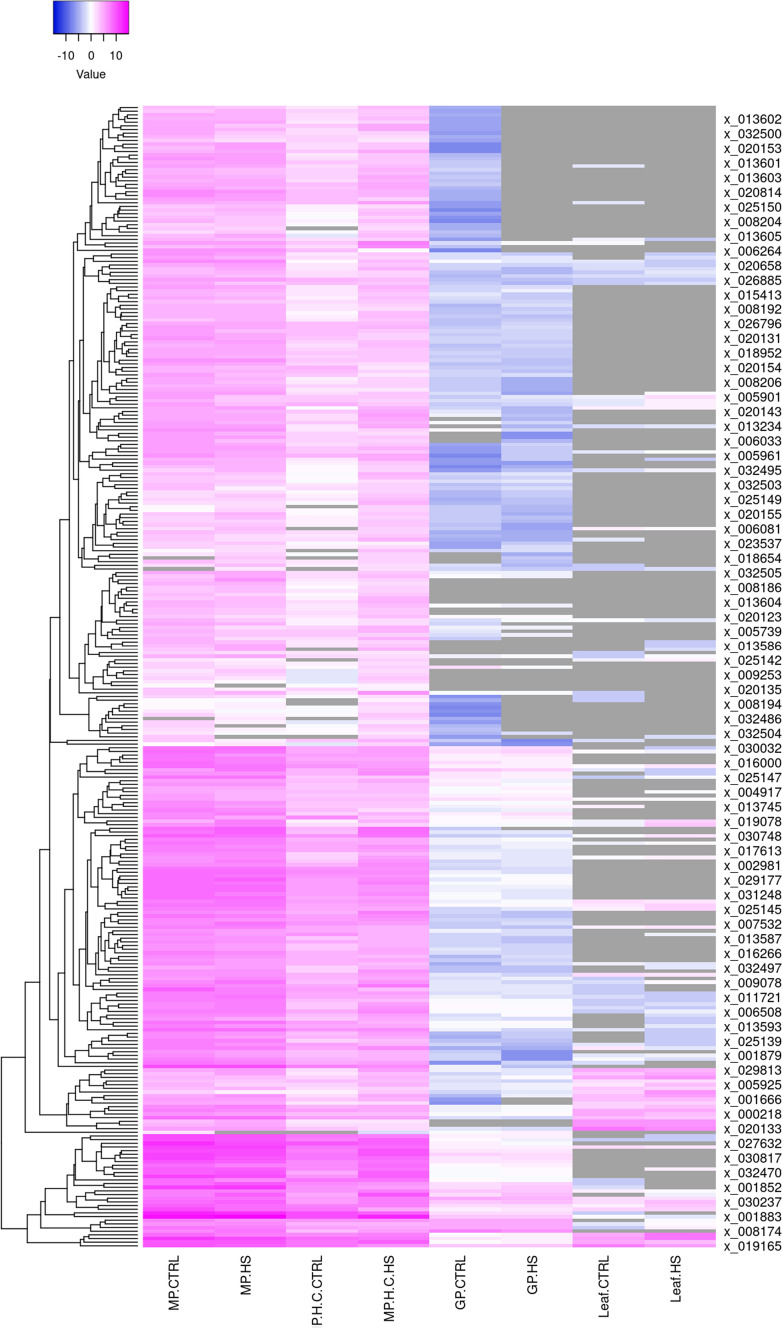
Heatmap of the expression abundance of XLOCs in the four RNAseq datasets under control and HS conditions

In a transcriptome experiment designed to identify differentially expressed genes that are pollen-specific and responsive to temperature stress, we compared the transcriptome of maturing pollen and cauline leaves exposed to heat stress (HS) (Rutley et al. in preparation; stress regime plot in Figure S1). Paired-end RNAseq data were generated from polyadenylated RNA from *Arabidopsis* (Col-0) mature pollen (MP) and cauline leaves grown under control (22 °C) or exposed to heat stress cycle for 3 days, referred to herein as MP_HS dataset and CL_dataset, respectively. The analysis of the RNAseq filtered raw data was performed using an in-house de novo assembly pipeline with the TAIR10 genome build as reference (Fig. 1a;Materials and methods section). Principal component analysis (PCA) of the normalized RNA-Seq data (Figure S2) showed that 78% and 83% of the variance in pollen and cauline leaf samples, respectively, could be explained by differences in conditions (control vs. HS). We also similarly analyzed recently published RNAseq data from mature pollen developed under a diurnal cycle of hot day and cold nights and control conditions (Rahmati Ishka et al. 2018), referred to herein as MP_Hot/Cold dataset. While interrogating the three RNAseq datasets, we assembled 400 transcriptional units (TUs, assigned with TCONS identifiers) originating from 312 intergenic expressed loci (XLOCs) that did not overlap with any loci annotated in TAIR10, with 41 of the XLOCs having two or more splice variants TCONS (Tables S2, S3). About 80% (246/312) of these transcripts were later annotated in the Araport11 database as expressed genes mostly without any other functional annotations (i.e., ‘unknown genes’) (v1.10.4, release 06/2016; Cheng et al. 2017), referred herein as ‘Araport recent’ genes. The remaining 66 genes are yet to be annotated (Fig. 1b, Table S3). The average depth of the MP_HS, MP_Hot/Cold, and CL_HS datasets was 63.7, 30.7, and 60.7 million reads, respectively (Table S4). Notwithstanding, while reads for all 312 XLOCs with overlapping coordinates were present in the MP datasets (all but one in MP_Cold/Hot dataset), 190 were absent in the CL dataset, suggesting that these transcripts are enriched in pollen (Fig. 2, Table 1, Table S4).

**Table 1:** Summary of novel genes from three independent pollen HS RNAseq experiments. Clear reads refer to the filtered raw data after removing adapter sequences, contamination, and low-quality reads

	Total clean reads (million)	Mean reads per sample (million)	PEXs (RPM > 1)	hPEXs (RPM > 10)	DEXs (Log2 ≥ 1, *P* ≤ 0.05)	PSXs	Pollen-specific DEX
MP_HS	382.0	63.7	312/312	269	37/312	230/312	21/230
MP_Hot/Cold	184.0	30.7	311/312	213	96/311	229/311	73/229
GP_HS	609.7	101.6	61/312	9	12/61	30/61	10/30

PEXs *pollen-expressed XLOC genes*; hPEXs *high PEX*

DEXs *differentially expressed XLOC genes*, PSXs *pollen-specific XLOC gene*

### The majority of the newly discovered XLOC genes encode pollen-specific long noncoding RNAs

Based on the criteria of RNA longer than 200 nucleotides or having ORF(s) shorter than 100 amino acids (Yu et al. 2019), 286 of the XLOCs were categorized as encoding long noncoding RNAs (lncRNAs) and 29 XLOCs contain ORFs ranging between 100 and 379 amino acids, suggesting that they may be protein-coding genes (Fig. 1c and Table S3). Indeed, 19/29 overlap with gene models in Araport11 (Table S3). Three remaining TUs transcribe RNA shorter than 200 nucleotides, and so do not meet the criteria of lncRNAs (Fig. 1c).

A subclass of lncRNAs are referred to as long intergenic noncoding RNAs (lincRNAs), defined as being located 500 or more bp away from annotated protein-coding genes, not encoding transposable elements (TEs), and not overlapping with natural antisense transcripts (NATs; Liu et al. 2012). As the MP_HS and MP_Hot/Cold RNAseq datasets are non-strand-specific, we employed only the two former criteria mentioned above, searching for lincRNAs. Among the 286 lncRNAs, 65% are located > 500 bp from the nearest gene model and 51% show overlaps with TEs (Table S3). Only 38 XLOCs (13.3%) meet two of these additional requirements for long intergenic noncoding RNA (referred to here as putative lincRNA; Fig. 1c).

Similarly, Deforges and co-authors recently identified 862 new lncRNA genes in RNAseq experiments in *Arabidopsis* seedlings that had no prior annotation in TAIR10, with about half of them later independently annotated in Araport11 v1.10.4 database release before their publication (Deforges et al. 2019). The dataset of Deforges et al. 2019 included RNAseq from whole *Arabidopsis* seedlings or roots and shoots from 12 experimental conditions, including high or low phosphate concentrations, and treatments with the plant hormones auxin (indole acetic acid, IAA), abscisic acid (ABA), methyl jasmonate (MeJA), or the ethylene precursor 1-aminocyclopropane-1-carboxylic acid (ACC). We, therefore, compared the coordinates of the newly identified TUs encoding genes in our pollen datasets with those of the genes identified in the Deforges et al. (2019) datasets. Of the 312 XLOCs identified in the MP_HS dataset, 14.4% and 7.1% overlapped completely or partially, respectively, whereas 230 of the pollen-identified expressed XLOC loci were not present within the sporophytic RNAseq datasets of Deforges et al. (2019) or our cauline leaves libraries (Fig. 1d, Table S5). Thus, this comparison between the pollen and sporophytic RNAseq datasets suggested that 78.5% of the identified XLOCs may exclusively express in pollen.

### The majority of the TUs are pollen-specific

**Fig. 3:**
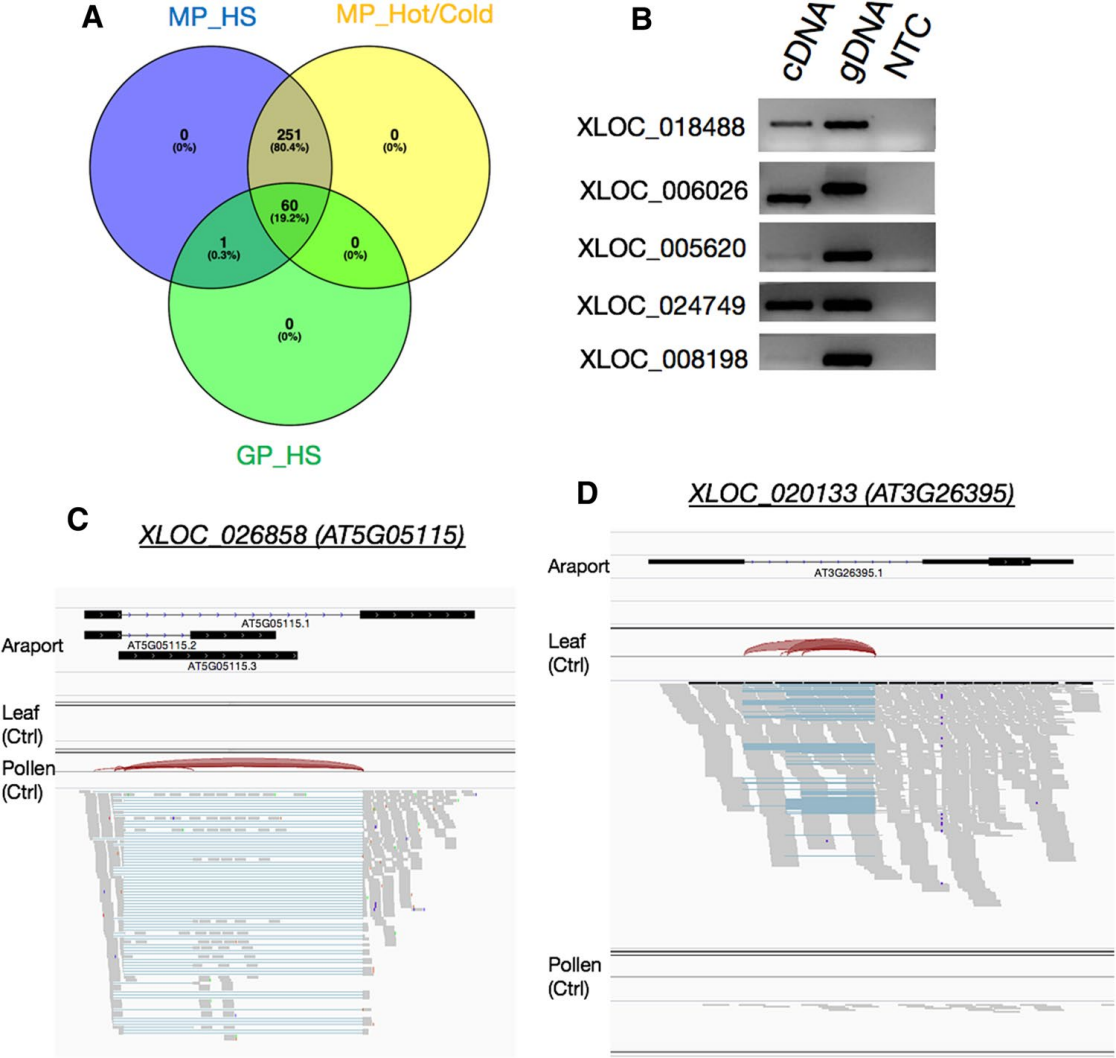
Comparison between the XLOCs expression between the three pollen RNAseq experiments and validation of expression. **a** Venn diagram of the presence of pollen-expressed XLOCs (PEXs; RPM > 1) in the three pollen datasets. **c** and **d** Visualization from IGV of pollen-specific **(c)** and cauline leaf-specific **(d)** XLOCs. Reads are shown aligned onto the reference Araport11 reference genome. Splice junctions are represented by brown arcs from the beginning to the end of the junction. gDNA, genomic DNA. NTC, no template control

We interrogated yet another pollen RNAseq dataset from an HS experiment in which pollen was germinated in vitro for 5 h at 24 °C or 35 °C for 5 h (GP_HS dataset; (Poidevin et al. 2020)). The GP_HS libraries are strand-specific, adding a higher order of resolution for producing gene models, providing us with the ability to exclude a potential expression of overlapping TEs on the opposite strand (Table S6). The average sequencing depth of the GP_HS sequencing was 101.6 million reads per sample (Table 1, Table S4). Yet, 75 of the 312 XLOCs were either absent or had a negligible abundance of mean read per million (RPM) lower than 0.05 in both the control and HS samples (Fig. 2, Table S5). Thus, given the depth of the RNAseqs, the differences in the presence of the XLOCs between the MP and GP experiments reflect the differences in the physiological and developmental phases of mature dry versus hydrated germinating pollen. Moreover, all the 312 XLOCs detected in mature pollen had the expression level > 1 RPM, whereas in the GP_HS, only 61 had > 1 RPM (Fig. 3a, Table 1, Table S5).

Therefore, we set the threshold indicating pollen expressed XLOC genes (PEXs) to 1 RPM.

We verified the expression of TUs in the RNAseq datasets using endpoint PCRs for five selected stress-responsive PEX with cDNA pooled from inflorescences grown under control and heat stress (Fig. 3b).

Among pollen-expressed genes, 269 and 213 from MP_HS and MP_Hot/Cold datasets, respectively, were shown higher than 10 mean RPM. All of these genes had none or low expression level (RPM < 1) in cauline leaves (Table 1, Table S5). As an example, using the Integrative Genome Viewer (IGV) tool, we verified that XLOC_0265858 lacks detectable reads in cauline leaves (Fig. 3c). In contrast, as a non-pollen exclusive example, we show IGV of XLOC_020133, one of only eight PEXs with higher expression in cauline leaves compared to pollen (Fig. 3d). Thus, we defined a total of 230 pollen-specific XLOCs (PSXs) as those genes not present and with RPM < 1 in either CL_HS or Deforges et al. (2019) RNAseq datasets. Interestingly, out of 61 PEXs in germinating pollen only 30 are pollen-specific (Table 1).

### Conservation among land plants

To search for conserved genes among the 312 XLOCs, we used CANTATAdb 2.0, a database of putative lncRNAs predicted from hundreds of RNAseq libraries from 39 species covering a broad diversity of land plant species (Szczesniak et al. 2019). Query entry for each of the 312 XLOC main TCONs identified homologs for only 30 genes in *A. lyrata* and an additional four genes in three species of other *Brassicaceae* family members (Table S7). All the identified conserved genes were expressed in both MP libraries, and six were also expressed in germinating pollen. In addition, around half (18/34) were pollen-specific. However, no putative lncRNA homologs were identified from more evolutionarily diverse species, suggesting that the majority of TUs encoding loci identified here originated within the *Brassicaceae* lineage (Table S7). The relatively low number of lncRNA homologs hits from CANTATAdb 2.0 may result from the libraries used to construct the database, as it is curated from transcriptomic studies not including pure pollen or pollen-enriched samples.

**Fig. 4:**
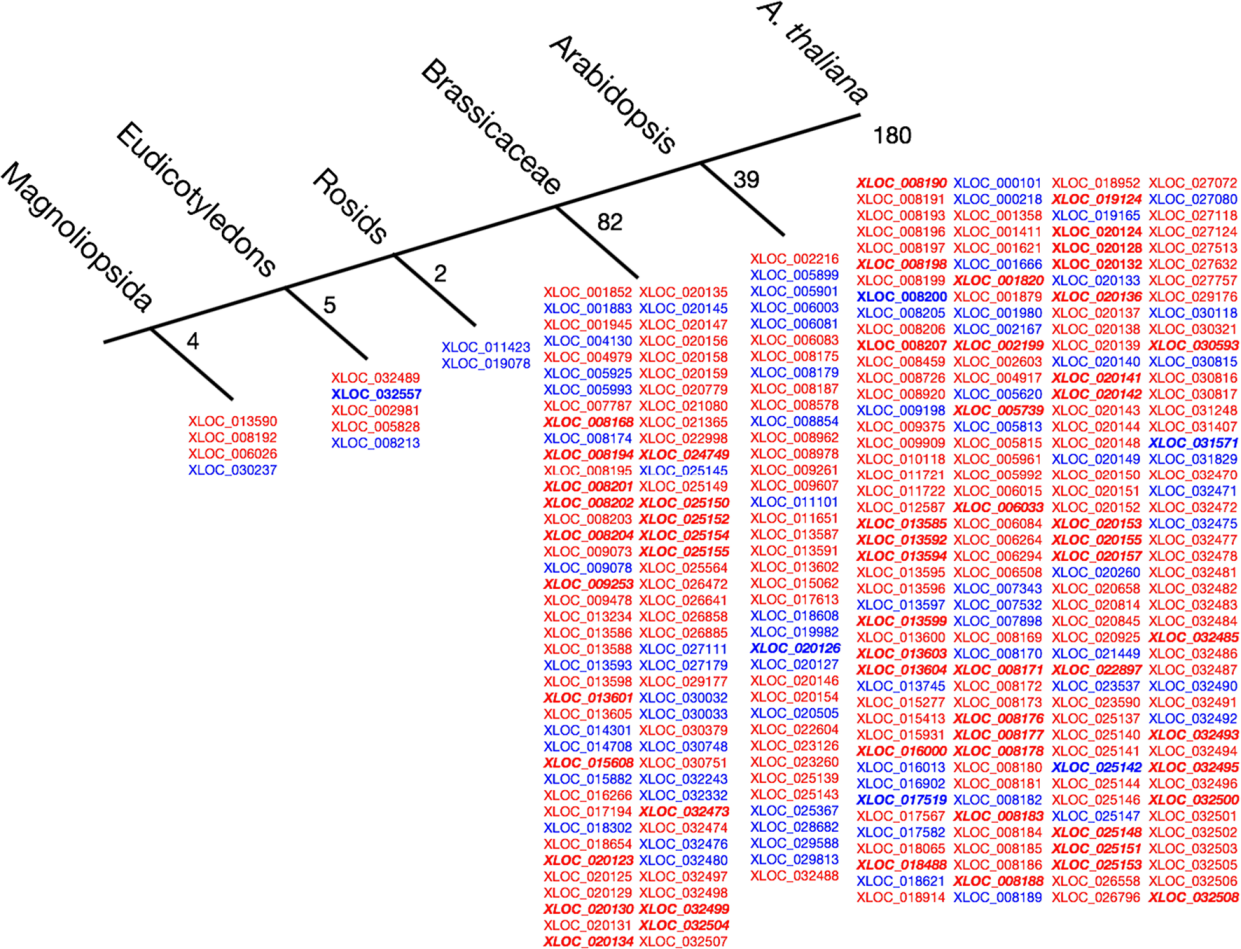
Phylostratigraphy of the 312 PEXs. Each XLOC was assigned to a phylostratum corresponding to the oldest phylogenetic node to which the gene could be traced. In red, pollen-specific XLOCs. In blue, XLOCs also expressed in sporophytic tissue in Deforges et al. (2019) or cauline leaf from MP_HS. In bold italics, novel unannotated genes

Because of the low number of matches obtained from CANTATA, we conducted a more comprehensive search for homologous genes using BLASTn megablast within the standard databases nucleotide collection (nr/nt). We then used the BLASTn results in a phylostratigraphic approach to determine the phylogenetic origin of each XLOC, assigning each XLOC to a phylostratum according to the oldest phylogenetic node to which the XLOC can be traced (Fig. 4). We found that the vast majority of XLOCs (301 of 312) evolved within the *Brassicaceae* family, including all novel yet unannotated TUs. Eleven PEXs matched homologous sequence in earlier divergent species, including four XLOCs assigned to the Magnoliopsida node, which have homologs in monocots (XLOC_013590, XLOC_008192, XLOC_006026, and XLOC_030237). Among the nine phylogenetically oldest PEXs, belonging to Eudicotyledons and Magnoliopsida, six are pollen-specific.

### Heat stress-driven differential expression in developing pollen

The induction of HSPs and HSFs in cauline leaves was severalfold higher than in the three pollen datasets, and the abundance (RPM) of the transcripts was 1–2 orders of magnitude higher, in most cases (Figure S3), in line with previous reports indicating that the heat stress response (HSR) in the male gametophyte is not robust as in vegetative tissues (Mascarenhas and Crone 1996; Muller and Rieu 2016). Yet, there were significant differences in the level of induction or lack of induction for some HSPs in pollen (Figure S3), which likely resulted from differences in the experimental setups (see Materials and methods section).

**Fig. 5:**
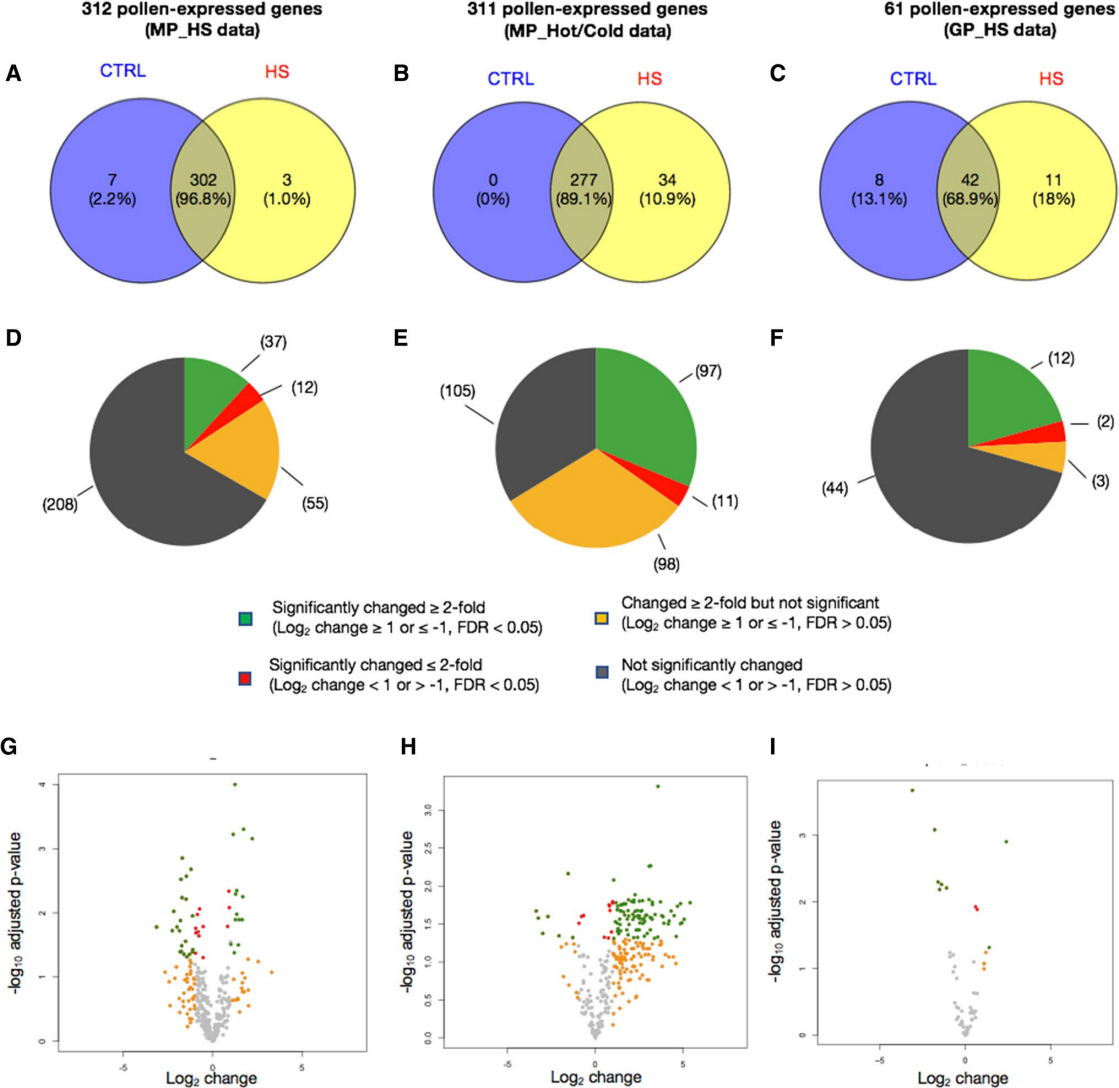
Differential expression of the PEXs in pollen RNA-seq libraries. **a**–**c** Comparison between control and HS conditions of the PEXs (RPM > 1) in each of the pollen experiments. **d–f** Pie charts showing the distribution of HS-responsive and non-responsive PEXs in each of the experiments. **g**–**i** Volcano plots showing the dynamic range of the differential expression of the PEXs

**Fig. 6:**
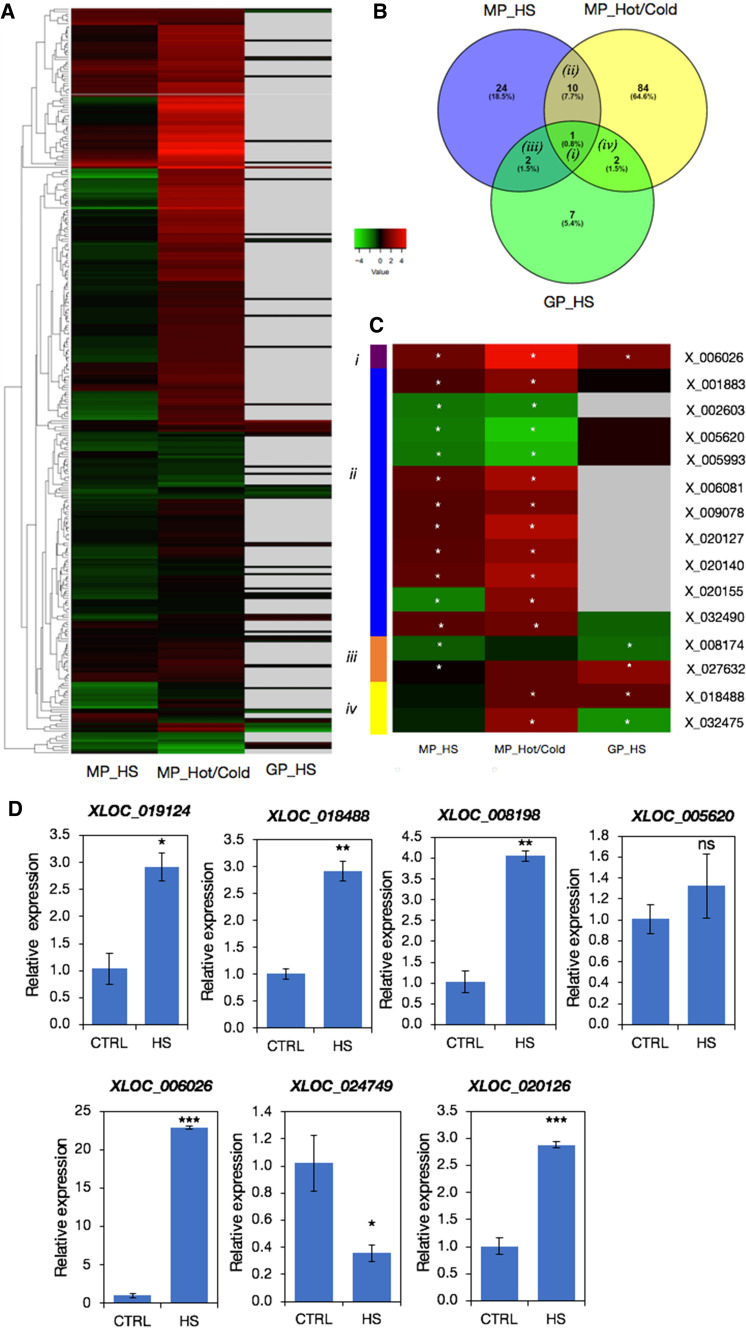
Comparison of differential expressed XLOCs (DEXs) in the three pollen experiments. **a** Heatmap of log_2_ changes for all 312 XLOCs in MP_HS, MP_Hot/Cold, and GP_HS libraries. **b** Venn diagram showing overlap of DEXs among the three pollen RNAseq datasets. **c** Heatmap of the DEXs present in two or more of the RNAseq datasets (sections *i–iv* in **b**. **d** Real-time PCR validation of differential expression of seven PEXs using cDNA from control and heat stress inflorescences

In each of the three independent pollen datasets, the majority of XLOCs were present in both control and heat stress conditions, having RPM values > 1 (Fig. 5a-c). Only 10, 34 and 19 XLOCs in the MP_HS, MP_Hot/Cold, and GP_HS, respectively, were specifically expressed in either one of the conditions with MP_HS. We then explored differential expression among the XLOCs in each of the three pollen datasets, comparing between control and HS conditions, using the criteria of a log fold change threshold of ≥ 1 or ≤  − 1 and significance of adjusted *p* value < 0.05. In the experiments in which the HS occurred during the pollen development, 37 and 97 of the XLOCs were deemed differentially expressed (DEXs) in the MP_HS and MP_Hot/Cold datasets, respectively (Fig. 5d, e). Nevertheless, a potentially higher percentage of the PEXs may be heat stress-responsive as an additional 12 and 11 genes passed the significance threshold for being differentially expressed in the MP_HS and MP_Hot/Cold datasets, respectively (Fig. 5d, e; Table S5). Additionally, of the 55 and 98 PEXs in the MP_HS and MP_Hot/Cold datasets, respectively, showing an average fold change ≥ 2 but with significance above the adjusted p value threshold (Fig. 5d, e), 24 were common to both (Figure S4). In germinated pollen, 12 PEXs showed clear differential expression under the in vitro heat stress treatment, and an additional five passed only the fold change or significant threshold suggesting potential stress responsiveness (Fig. 5f). HS cycle of 22–37 °C/16° day/night (MP_HS) resulted in similar numbers of up- and down-regulated genes (Fig. 5g), whereas pollen developed under a hot day (peaking at 40 °C at noon) and cold nights (1 °C) had far more upregulated genes than down-regulated genes (Fig. 5h). The differences in the pattern of DEXs (Figs. 5g–i, 6a) potentially reflect the difference between the heat stress regimes and the physiological phase of the pollen during the experiment. Consequently, there was a relatively small overlap among the DEGs between the three HS experiments (Fig. 6a, b, Figures S4-S6), suggesting that the experimental setups impacted the level and the direction of expression in most of the HS-responsive XLOCs. Yet, 42% of the PEXs showed significantly responded to HS across all three experiments (130 DEXs). Only one DEX, XLOC_006026, was common to all three HS experiments and changed in the same direction in all the three experiments, suggesting that it might be a part of the core heat stress response in pollen. An additional 10 DEGs were common to both mature pollen datasets (Fig. 6b, c). Nine of these 10 DEGs were showing similar expression trend following heat stress in the mature pollen datasets (Fig. 6b). Of the four DEX from GP_HS that were also differentially expressed in one of the other datasets, three showed a similar response trend to heat stress.

We further conducted quantitative real-time PCRs to validate differential expression during HS for six PSXs and one PEX that is not pollen-specific, XLOC_005620. To this end, we used cDNA from inflorescence (flower buds and open flowers) of wild-type Col-0 plants grown under control conditions or exposed to 3-day HS regime as in the MP_HS experiment (Figure S1). We show that five of six PSXs were upregulated in response to heat stress (Fig. 6d). Among the six PSXs, the induction of *XLOC_006026*, which increased in all three experiments (Table S8), was the most prominent, with a 23-fold increase (Fig. 6d). In comparison, *XLOC_019124*, *XLOC_ 018488*, *XLOC_008198*, and *XLOC_020126*, which were induced in the MP_Hot/Cold experiment (Table S8), only increased threefold to fourfold during HS in the inflorescences (Fig. 6d). In contrast, *XLOC_005620*, which significantly decreased in both mature pollen HS datasets (Fig. 6c), did not change in the qRT-PCR experiment (Fig. 6d), which might be due to non-gametophyte-specific expression. The real-time PCR results validate the DEXs identified in the datasets from three pollen experiments and support the notion that some of the PEXs induced during HS may play a role in the stress acclimation of the male gametophyte.

### XLOCs potentially regulated by miRNAs

A major mechanism of gene expression regulation is mediated through small RNAs, including microRNAs (miRNAs), which can impact mRNA degradation and translational repression. Similar to mRNA, lncRNAs can be targets of miRNAs and act as miRNA decoys, sequestering specific miRNAs (Franco-Zorrilla et al. 2007). To predict which PEXs are potentially targeted by miRNA binding, we used the psRNATarget tool employing the confidence cutoff threshold of 3.0 (Dai et al. 2018). We identified 50 PEXs as putative targets of one or more *Arabidopsis* miRNAs, the majority of which were predicted to be processed by cleavage rather than translational inhibition (Table S9; Figure S8A). Several miRNAs have been characterized as pollen-expressed under control conditions (Borges et al. 2011). While our RNA-seq libraries are unlikely to contain mature miRNAs, we looked for reads mapping to the pollen-expressed primary (pri)-miRNAs in the MP_HS dataset. We found several reads mapping to miR447a (Table S9, Figure S8B), an *A. thaliana*-specific microRNA (Rathore et al. 2016), in the control treatment but not following HS. Correspondingly, while the expression of pri-miRNA447a decreased in HS, expression of its putative target XLOC_032495 (TCONS_00050386) increased in both MP_HS and MP_Hot/Cold (Figure S8C). miRNA447a is down-regulated in cold-imbibed seeds vs. dry seeds (Sarkar Das et al. 2018) and hypoxia-treated roots vs. control (Moldovan et al. 2010), pointing to it being a stress-regulated, and possibly stress regulating, miRNA.

### Periodic movement in ribosome-protected mRNA fragments (RPFs) indicates translation potential for some XLOCs

Ribosome profiling, also known as ribosome sequencing (Riboseq), is a powerful method for identifying transcripts engaged with ribosomes and allows for the likelihood prediction of transcripts undergoing in vivo translation. In parallel with the RNAseq of the GP_HS experiment, sequencing of ribosome-protected fragments (RPF) was performed from the same in vitro HS experiment of in vitro germinated pollen (Poidevin et al. 2020). As the germinated pollen sequencing libraries are strand-oriented, we determined the coding strand for each of these XLOC genes (Table S6). We found consistent alignments for a total of 75 RPFs (RPF-GP_HS dataset) to 73 XLOCs within the RNA (GP_HS dataset), where XLOC_020146 and XLOC_005993 had RPF transcripts from both strands (Table S6). We focused on the 45 XLOCS with RPF reads values ≥ 1 RPM in either control and HS (35 °C) or both, including XLOC_020146 plus and minus transcripts (Table S10).

**Fig. 7:**
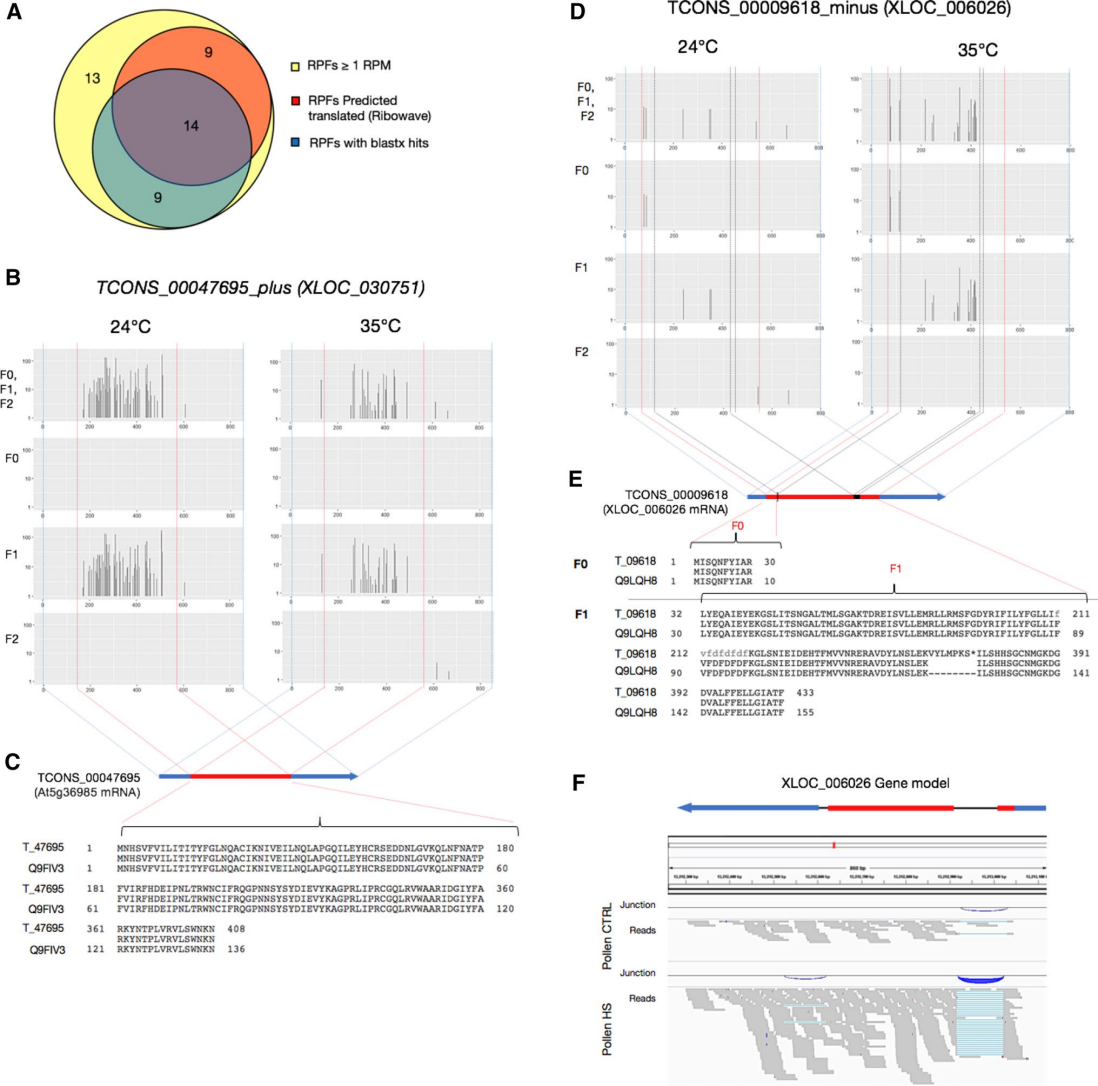
Periodic movement in ribosome-protected mRNA. **a** Venn comparison of significant RPFs (RPM > 1) showing overlaps between RPFs predicted to move on ribosomes and those with ORFs showing homology to protein sequences. **b**–**f** characterization of the HS-induced PEXs that are predicted to be translated. B. Periodic footprint of the P-sites (PF P-sites) plot along the three frames (F0-F2) of the transcripts of XLOC_030751, generated by the RiboWave algorithm, indicating the activity of elongating ribosomes. **d** PF P-sites plot along the three frames (F0-F2) of the transcripts of XLOC_006026. **c** and **e** show the mRNA model of XLOC_030751 (At5g36985) and XLOC_006026, respectively. Blue boxes—UTRs, red boxes—CDSs. The black box in **e** represents the unspliced intron-2. The dotted blue, red, and black dotted vertical lines in **b** and **d** indicate the transcripts’ borders, intron–exon junctions, and intron-2 borders, respectively. **f** XLOC_006026 gene model corresponding to the IGV plot of the RNAseq reads in control and HS mature pollen, generated from the MP_HS dataset. Splice junctions are represented by blue arcs

To gain further insight into the translational profiles revealed by the RPF values, we used the RiboWave v1.0 tool (Xu et al. 2018), a pipeline able to denoise the original RPF signal by extracting the periodic footprint of the P-sites (PF P-sites) of actively elongating ribosomes. The PF P-site values were decomposed into the three different frames, to determine which frame is likely translated by the ribosome. One limitation of the XLOCs annotations is that no clear definition of exons has been established that allows the discrimination between coding and untranslated regions (UTRs). Therefore, we could not exploit the statistical significance of translational prediction of the RiboWave algorithm. However, visual inspection of the denoised PF P-site tracking allowed us to define a minimum number of five PF P-sites in the same frame as a proxy of an increased likelihood for translation. From the initial 45 XLOCs, 23 XLOCs passed this filter and could be considered as very likely translated (Fig. 7a, Table S10).

Additionally, a blastx (nr) for all 312 XLOCs (limited to *Arabidopsis thaliana*) identified partial hits (50–96% identity) or full match hits (≥ 97% identity) with sequences of predicted proteins (*E* value ≤ 1e − 7) for l16 (37%) of the genes, of which 92 (30%) are lncRNAs (Table S11). The proportion of blastx hits positives within the 75 RPFs XLOCs was 51%, and 60% (14 of 23) of the RPFs with the minimum number of five PF P-sites (Fig. 7a, Table S10, S11) corresponded with the increased likelihood of these XLOCs being translated. Interestingly, 6 of these 14 XLOCs encode for predicted UniProt proteins, the putative products for two of which, XLOC_030571 and XLOC_032470, are homologs of pollen-specific self-incompatibility proteins.

In Fig. 7b and d, we present the PF P-site periodicity plots for two of these TUs, TCONS_00047695 (*XLOC_030571*) and TCONS_00009618 (*XLOC_006026*), both pollen-specific and HS-induced expressed loci (Table S5).

XLOC_030751, annotated in Araport11 as unknown gene AT5G36985, is intronless with an ORF (DOC S3) coding for self-incompatibility protein homolog 25 (SPH25) (Fig. 7c). The periodicity plot for *XLOC_030571* clearly shows that only one reading frame (F1) is being translated, with the PF P-sites concentrated within the CDS along the transcript.

In contrast, the periodicity plot of *XLOC_006026* (Figs. 3, 4, 6) revealed PF P-sites in two frames, F0 and F1 (Fig. 7d), suggesting that the TU contains an unprocessed intron. The blastx search identified a near-perfect identity with a phosphoenolpyruvate carboxykinase (PEPCK) protein. The pairwise alignment of the translated XLOC_006026 mRNA with the protein sequence resulted in two ways split, with the first part showing 100% identity with the first 10 amino acids. The second part is identical with the other 145 amino acids, except for seven extra amino acids and a stop codon in the C-terminus (Fig. 7e), corresponding to the two frames being read (Fig. 7d). Inspection of the XLOC _006026 CDS revealed two premature stop codons at + 37 and + 347; the first originated by what seems like an inaccurate exon1-intron1 site splicing event leading to frameshift, and the second by the inclusion of intron 2 (Doc S4, Fig. 7f). Yet, although the vast majority of XLOC _006026 transcripts seem to be incorrectly processed, IGV output shows that few transcripts may still be fully and correctly spliced to produce the full-length protein-coding sequence CDS (Fig. 7f).

## Discussion

### Discovery of novel transcripts in *Arabidopsis* pollen

Novel genes that might only be expressed in a restricted context, such as a single cell type alone or in combination with stress, are only slowly being incorporated into reference genome annotations. The reason for overlooking these genes is partly because RNAseq studies tend to focus on known genes and exclude sequence reads that do not match an existing reference genome. Therefore, it is not surprising that even 20 years after the first draft of the *Arabidopsis* genome was published (Arabidopsis Genome 2000), additional new genes are still being discovered. The recent Araport11 annotation update released in 2016 added more than 600 and 5000 novel protein-coding and noncoding RNAs, respectively, relying predominantly on RNAseqs from sporophytic tissues (Cheng et al. 2017). Comparison between microarray datasets of flower, roots, and leaves identified 32% of ~ 3700 lincRNAs in *Arabidopsis* as showing preferential expression in one of these organs (Liu et al. 2012). Epigenetic reprogramming taking place during plant sexual reproduction involves the overall reduction in DNA methylation in the germline during gametogenesis, allowing the potential expression of otherwise silent genetic elements, including TEs, endogenous protein-coding genes, and introduced transgenes (Kawashima and Berger 2014). It is therefore likely that the 312 PEX genes (pollen-expressed intergenic expressed loci) reported herein have gone unnoticed for such a long time is due, in part, for their expression being mostly restricted to pollen (Figs. 1, 2, 3), rather than the depth of the RNA sequencing (Table S4).

Compared with sporophytic tissues, such as root or leaf, which are composed of multiple cell types with different specialized functions, the male gametophyte is simple, containing a haploid vegetative nucleus and two sperm cells in tricellular pollen. This feature is advantageous for obtaining a large quantity of uniform cell-type-specific for any omics profiling. In comparison, obtaining a uniform sporophytic single-cell population from homogenized tissue for transcriptomics requires the isolation of tag-labeled cells by fluorescent-activated cell sorter (FACS). Moreover, RNA extracted from the enriched isolated cells needs to be amplified to construct a library, which is biased toward transcripts with relatively high abundance, and transcripts with low copy number are often omitted (Efroni and Birnbaum 2016).

In recent years, RNAseq studies have added many new genes to the genome annotations of *Arabidopsis* and human, organisms completely sequenced two decades ago. Genomic studies based on various transcriptomic platforms identified thousands of lncRNAs in diverse animal and plant genomes, including over 58,000 in the human genome (Ulitsky 2016). As lncRNAs are more tissue-specific and expressed at lower levels than protein-coding mRNAs (Ulitsky 2016), it is plausible that future studies will identify many more yet unidentified cell-type-specific lncRNA loci in the *Arabidopsis* genome.

### The majority of the lncRNAs PEX genes reflect the age of the *Arabidopsis* pollen transcriptome

Our finding that the vast majority of the 312 PEX genes belong to the *Brassicaceae* family (Fig. 4), 180 of which are *A. thaliana* specific, suggests these genes evolved relatively recently. These findings are in agreement with the feature of lncRNAs evolving more rapidly compared with protein-coding genes (Ulitsky 2016; Yu et al. 2019; Ruiz-Orera et al. 2020). The transcriptome of the male gametophyte appears to be enriched for recently evolved genes (i.e., lineage-specific genes and orphan genes lacking homologs in other lineages). These young genes include short peptides, intergenic transcripts, long noncoding RNAs (lncRNAs), and de novo genes at their transitory stages, also known as proto-genes (Cui et al. 2015). Similarly, 180 and 39 of the PEXs are species-specific and taxon-specific orphan genes, respectively (Fig. 4), indicating that they are young genes that recently evolved in the *Brassicaceae* lineage. The emergence of de novo genes from non-genic regions is relatively frequent in eukaryotes and may be part of a progressive evolutionary process that starts with the expression of intergenic regions to proto-genes and, finally, functional genes (Cui et al. 2015).

A survey of RNAseq datasets from 15 diverse flowering plant species indicated that transcription from unannotated intergenic regions is quite frequent in plants, but a large part of it is due to random non-functional transcriptional noise (Lloyd et al. 2018). Yet, predictions based on a computational analysis in *Arabidopsis* indicated that 38% of intergenic transcribed regions and 40% of the annotated ncRNAs have similar features to protein-coding or RNA genes and are likely functional (Lloyd et al. 2018). Since the majority of the 312 XLOC genes are relatively abundant (RPM > 10), expressed exclusively in developing/maturing pollen, and HS-responsive, we postulate that most, if not all, are functional genes. The function of these young genes may be diverse, including direct or indirect gene expression control. For example, the 50 PEXs we identified having miRNA targets may function as target mimics and molecular sponges that inhibit the action from a subset of miRNAs. Furthermore, 30% of the lncRNA PEXs contain smORFs that matches protein sequences. Indication of interaction with ribosomes, for those present in the riboseq dataset, and the demonstration of movement along the ribosome (active PF-P footprint) indicated that some of the lncRNAs produce micro-proteins and proteins that may be functional (DOC S2, Fig. 7a, Table S11). Functional studies in a broad range of species from yeast to humans demonstrate that microproteins can functionally impact development and physiology (Plaza et al. 2017). In the context of pollen, there are several examples of microproteins having a profound impact on pollen growth and fertilization, including self-incompatibility, tube growth, and sperm release inside the ovule (Cui et al. 1999; Dong et al. 2013; Uebler et al. 2015; Ge et al. 2017).

The occurrence of XLOC_030751 and XLOC_032470 encoding for self-incompatibility-related protein homologs among the 23 PEXs PF P-sites-positive very likely to be translated (Fig. 7a, Table S10) lend support for the plausibility that some of the ORF-containing PEXs are functional. Interestingly, the expression of both self-incompatibility related genes is relatively abundant in mature pollen, and they responded to the stress regime in the MP_Hot/Cold experiment (Table S5). Direct experimental evidence at the protein level is needed to determine their actual presence and whether they function in pollen–pistil interactions. Yet, they might be involved in switching from selfing to outcrossing mode of mating, which is employed in about half of the species in the *Brassicaceae* family (Nasrallah 2019).

### Potential involvement of PEX in pollen HSR

The limited HSR of pollen compared to cauline leaves indicated by the activation of *HSPs* (Figure S3) may account, at least in part, for its increased thermosensitivity, but also suggests that pollen has distinct requirements for coping with high temperatures. A major conceptual difference between pollen and sporophytic cells is that the latter can respond to HS by reducing metabolism into ‘survival mode,’ waiting for better growth conditions to resume growth or other physiological activities, whereas developing pollen and pollen tubes have a limiting window of time to properly mature and fertilize an ovule (Rahmati Ishka et al. 2018). We found that the expression of a large proportion (~ 42%) of the 312 PEXs significantly changed ≥ twofold at least in one of the pollen HS experiments datasets (Fig. 6b) and many of the other 58% are potentially HS-responsive (Fig. 5e–f). A more comprehensive survey of the expression pattern of all these potential DEXs at different stages of pollen development along with different time points during an HS is required to determine their relevance to pollen HSR. However, given that the majority of the PEX specifically express in pollen (i.e., PSXs), it is likely that some of them either function in the pollen HSR, or are at least responsive to regulatory pathways that are controlling the HSR.

Several of the PSXs that might function at the core pollen HSR include XLOC_006026, which was induced in all three pollen experiments, and others that were increased in two of the pollen datasets, for example XLOC_027632, XLOC_002603 (Fig. 6c), XLOC_009073, XLOC_009261 (Figure S5), XLOC_008196, XLOC_008185 (Figure S6), and XLOC_008726 (Figure S6). XLOC_006026 is intriguing as it is one of the most conserved PEXs that might code for a functional PEPCK, an enzyme involved in malate metabolism and gluconeogenesis. The closest PEPCK homolog of XLOC_006026 is AtPKC1 (AT4G37870), with 55.6% identity, which was shown to function in malate metabolism in stomatal closure and drought tolerance in young *Arabidopsis* plants (Penfield et al. 2012). PEPCK catalyzes the reversible decarboxylation of oxaloacetate to yield phosphoenolpyruvate (PEP) and CO_2_ at the expense of ATP. Since PEP is a precursor for either the glucose and shikimate biosynthesis pathways or to pyruvate, it is situated at an important crossroad in plant metabolism, lying between organic and amino acids, lipids and sugars (Lea et al. 2001). Increased demand for carbohydrates during HS was suggested to contribute to the enhanced thermosensitivity of pollen (Rieu et al. 2017). HS was shown to deplete the level of accumulated starch and soluble sugars in developing pollen, whereas HS-tolerant tomato genotypes were better able to maintain pollen starch and sugar levels than sensitive genotypes (Pressman et al. 2002; Firon et al. 2006; Sato et al. 2006). Therefore, the induction of PEPCK activity might help pollen accumulate specific sugars and better tolerate HS. It would therefore be highly interesting to test the potential impact of XLOC_006026 on pollen development and activity both during favorable conditions and elevated temperatures.

### Concluding remarks

It was recently suggested that lncRNAs make suitable environmental sensors or effectors to help plants adapt to changing environments, as their expression is extremely responsive to stresses and they evolve rapidly compared with protein-coding genes (Yu et al. 2019). However, functional studies of the involvement of lncRNAs in pollen development and physiology, let alone pollen acclimation to stress, are still at their earliest stages. The large proportion of genes encoding lncRNAs added to the *Arabidopsis* genome annotation in the previous Araport11 update, together with the thousands that have been identified in many other plant species in RNAseq experiments, raise the question about whether they have a function. Finding whether these lncRNAs play a significant role and how do they perform their function is currently a major challenge in plant biology. Our identification in pollen and characterization of the novel and ‘Araport recent’ PEXs genes significantly provide a foundation for understanding potential functions for some of these lncRNAs in pollen development and HSR.

#### Author contribution statement

NR, LP, and TD conducted bioinformatic data analysis; NR and AR performed PCR analyses. LP and GL designed and performed the experiments; RLT, KAS, and JF performed bioinformatics and statistical analyses; NR and GM wrote the manuscript and prepared the figures. GM, JFH, and AF conceived the research and supervised the project. All authors read and approved the manuscript.

## Electronic supplementary material

Below is the link to the electronic supplementary material.

Supplementary file1 (PDF 1583 kb)

Supplementary file1 (PDF 8293 kb)

Supplementary file1 (PDF 50 kb)

Supplementary file1 (PDF 304 kb)

Supplementary file1 (DOCX 1357 kb)

Supplementary file2 (XLSX 471 kb)

## References

[CR1] Anders S, Huber W (2010). Differential expression analysis for sequence count data. Genome Biol.

[CR2] Arabidopsis Genome I (2000). Analysis of the genome sequence of the flowering plant *Arabidopsis thaliana*. Nature.

[CR3] Begcy K, Nosenko T, Zhou LZ, Fragner L, Weckwerth W, Dresselhaus T (2019). Male sterility in maize after transient heat stress during the tetrad stage of pollen development. Plant Physiol.

[CR4] Bolger AM, Lohse M, Usadel B (2014). Trimmomatic: a flexible trimmer for Illumina sequence data. Bioinformatics.

[CR5] Borges F, Pereira PA, Slotkin RK, Martienssen RA, Becker JD (2011). MicroRNA activity in the *Arabidopsis* male germline. J Exp Bot.

[CR6] Cheng CY, Krishnakumar V, Chan AP, Thibaud-Nissen F, Schobel S, Town CD (2017). Araport11: a complete reannotation of the *Arabidopsis* thaliana reference genome. Plant J.

[CR7] Chettoor AM, Givan SA, Cole RA, Coker CT, Unger-Wallace E, Vejlupkova Z, Vollbrecht E, Fowler JE, Evans MM (2014). Discovery of novel transcripts and gametophytic functions via RNA-seq analysis of maize gametophytic transcriptomes. Genome Biol.

[CR8] Cui Y, Brugiere N, Jackman L, Bi YM, Rothstein SJ (1999). Structural and transcriptional comparative analysis of the S locus regions in two self-incompatible Brassica napus lines. Plant Cell.

[CR9] Cui X, Lv Y, Chen M, Nikoloski Z, Twell D, Zhang D (2015). Young genes out of the male: an insight from evolutionary age analysis of the pollen Transcriptome. Mol Plant.

[CR10] Dai X, Zhao PX (2011). psRNATarget: a plant small RNA target analysis server. Nucleic Acids Res.

[CR11] Dai X, Zhuang Z, Zhao PX (2018). psRNATarget: a plant small RNA target analysis server (2017 release). Nucleic Acids Res.

[CR12] Deforges J, Reis RS, Jacquet P, Vuarambon DJ, Poirier Y (2019). Prediction of regulatory long intergenic non-coding RNAs acting in trans through base-pairing interactions. BMC Genom.

[CR13] Di C, Yuan J, Wu Y, Li J, Lin H, Hu L, Zhang T, Qi Y, Gerstein MB, Guo Y, Lu ZJ (2014). Characterization of stress-responsive lncRNAs in *Arabidopsis thaliana* by integrating expression, epigenetic and structural features. Plant J.

[CR14] Ding J, Lu Q, Ouyang Y, Mao H, Zhang P, Yao J, Xu C, Li X, Xiao J, Zhang Q (2012). A long noncoding RNA regulates photoperiod-sensitive male sterility, an essential component of hybrid rice. Proc Natl Acad Sci U S A.

[CR15] Dong X, Wang D, Liu P, Li C, Zhao Q, Zhu D, Yu J (2013). Zm908p11, encoded by a short open reading frame (sORF) gene, functions in pollen tube growth as a profilin ligand in maize. J Exp Bot.

[CR16] Efroni I, Birnbaum KD (2016). The potential of single-cell profiling in plants. Genome Biol.

[CR17] Firon NSR, Peet MM, Pharr DM, Zamski E, Rosenfeld K, Althan L, Pressman E (2006). Pollen grains of heat tolerant tomato cultivars retain higher carbohydrate concentration under heat stress conditions. Sci Hortic (Amst).

[CR18] Fragkostefanakis S, Mesihovic A, Hu Y, Schleiff E (2016). Unfolded protein response in pollen development and heat stress tolerance. Plant Reprod.

[CR19] Franco-Zorrilla JM, Valli A, Todesco M, Mateos I, Puga MI, Rubio-Somoza I, Leyva A, Weigel D, Garcia JA, Paz-Ares J (2007). Target mimicry provides a new mechanism for regulation of microRNA activity. Nat Genet.

[CR20] Ge Z, Bergonci T, Zhao Y, Zou Y, Du S, Liu MC, Luo X, Ruan H, Garcia-Valencia LE, Zhong S, Hou S, Huang Q, Lai L, Moura DS, Gu H, Dong J, Wu HM, Dresselhaus T, Xiao J, Cheung AY, Qu LJ (2017). *Arabidopsis* pollen tube integrity and sperm release are regulated by RALF-mediated signaling. Science.

[CR21] Jacott CN, Boden SA (2020). Feeling the heat: developmental and molecular responses of wheat and barley to high ambient temperatures. J Exp Bot.

[CR22] Johnson-Brousseau SA, McCormick S (2004). A compendium of methods useful for characterizing *Arabidopsis* pollen mutants and gametophytically-expressed genes. Plant J.

[CR23] Kawashima T, Berger F (2014). Epigenetic reprogramming in plant sexual reproduction. Nat Rev Genet.

[CR24] Kim D, Langmead B, Salzberg SL (2015). HISAT: a fast spliced aligner with low memory requirements. Nat Methods.

[CR25] Krishnakumar V, Contrino S, Cheng CY, Belyaeva I, Ferlanti ES, Miller JR, Vaughn MW, Micklem G, Town CD, Chan AP (2017). ThaleMine: a warehouse for *Arabidopsis* data integration and discovery. Plant Cell Physiol.

[CR26] Lea PJ, Chen ZH, Leegood RC, Walker RP (2001). Does phosphoenolpyruvate carboxykinase have a role in both amino acid and carbohydrate metabolism?. Amino Acids.

[CR27] Liao Y, Smyth GK, Shi W (2014). featureCounts: an efficient general purpose program for assigning sequence reads to genomic features. Bioinformatics.

[CR28] Liu J, Jung C, Xu J, Wang H, Deng S, Bernad L, Arenas-Huertero C, Chua NH (2012). Genome-wide analysis uncovers regulation of long intergenic noncoding RNAs in *Arabidopsis*. Plant Cell.

[CR29] Lloyd JP, Tsai ZT, Sowers RP, Panchy NL, Shiu SH (2018). A model-based approach for identifying functional intergenic transcribed regions and noncoding RNAs. Mol Biol Evol.

[CR30] Lohani N, Singh MB, Bhalla PL (2020). High temperature susceptibility of sexual reproduction in crop plants. J Exp Bot.

[CR31] Loraine AE, McCormick S, Estrada A, Patel K, Qin P (2013). RNA-seq of *Arabidopsis* pollen uncovers novel transcription and alternative splicing. Plant Physiol.

[CR32] Love MI, Huber W, Anders S (2014). Moderated estimation of fold change and dispersion for RNA-seq data with DESeq2. Genome Biol.

[CR33] Luria G, Rutley N, Lazar I, Harper JF, Miller G (2019). Direct analysis of pollen fitness by flow cytometry: implications for pollen response to stress. Plant J.

[CR34] Lv Y, Hu F, Zhou Y, Wu F, Gaut BS (2019). Maize transposable elements contribute to long non-coding RNAs that are regulatory hubs for abiotic stress response. BMC Genomics.

[CR35] Ma L, Bajic VB, Zhang Z (2013). On the classification of long non-coding RNAs. RNA Biol.

[CR36] Mascarenhas JP, Crone DE (1996). Pollen and the heat shock response. Sex Plant Reprod.

[CR37] Mesihovic A, Iannacone R, Firon N, Fragkostefanakis S (2016). Heat stress regimes for the investigation of pollen thermotolerance in crop plants. Plant Reprod.

[CR38] Mishra A, Bohra A (2018). Non-coding RNAs and plant male sterility: current knowledge and future prospects. Plant Cell Rep.

[CR39] Moldovan D, Spriggs A, Yang J, Pogson BJ, Dennis ES, Wilson IW (2010). Hypoxia-responsive microRNAs and trans-acting small interfering RNAs in *Arabidopsis*. J Exp Bot.

[CR40] Muller F, Rieu I (2016). Acclimation to high temperature during pollen development. Plant Reprod.

[CR41] Nasrallah JB (2019). Self-incompatibility in the *Brassicaceae*: regulation and mechanism of self-recognition. Curr Top Dev Biol.

[CR42] Plaza S, Menschaert G, Payre F (2017). In search of lost small peptides. Annu Rev Cell Dev Biol.

[CR43] Poidevin L, Forment J, Unal D, Ferrando A (2020). Transcriptome and translatome changes in germinated pollen under heat stress uncover roles of transporter genes involved in pollen tube growth. BioRxiv.

[CR44] Pressman E, Peet MM, Pharr DM (2002). The effect of heat stress on tomato pollen characteristics is associated with changes in carbohydrate concentration in the developing anthers. Ann Bot.

[CR45] Quinlan AR, Hall IM (2010). BEDTools: a flexible suite of utilities for comparing genomic features. Bioinformatics.

[CR46] Rahmati Ishka M, Brown E, Weigand C, Tillett RL, Schlauch KA, Miller G, Harper JF (2018). A comparison of heat-stress transcriptome changes between wild-type *Arabidopsis* pollen and a heat-sensitive mutant harboring a knockout of cyclic nucleotide-gated cation channel 16 (cngc16). BMC Genom.

[CR47] Rathore P, Geeta R, Das S (2016). Microsynteny and phylogenetic analysis of tandemly organised miRNA families across five members of *Brassicaceae* reveals complex retention and loss history. Plant Sci.

[CR48] Rieu I, Twell D, Firon N (2017). Pollen development at high temperature: from acclimation to collapse. Plant Physiol.

[CR49] Ruiz-Orera J, Villanueva-Canas JL, Alba MM (2020). Evolution of new proteins from translated sORFs in long non-coding RNAs. Exp Cell Res.

[CR50] Sarkar Das S, Yadav S, Singh A, Gautam V, Sarkar AK, Nandi AK, Karmakar P, Majee M, Sanan-Mishra N (2018). Expression dynamics of miRNAs and their targets in seed germination conditions reveals miRNA-ta-siRNA crosstalk as regulator of seed germination. Sci Rep.

[CR51] Sato S, Kamiyama M, Iwata T, Makita N, Furukawa H, Ikeda H (2006). Moderate increase of mean daily temperature adversely affects fruit set of *Lycopersicon esculentum* by disrupting specific physiological processes in male reproductive development. Ann Bot.

[CR52] Slattery RA, Ort DR (2019). Carbon assimilation in crops at high temperatures. Plant Cell Environ.

[CR53] Suzuki G (2009). Recent progress in plant reproduction research: the story of the male gametophyte through to successful fertilization. Plant Cell Physiol.

[CR54] Swarbreck D, Wilks C, Lamesch P, Berardini TZ, Garcia-Hernandez M, Foerster H, Li D, Meyer T, Muller R, Ploetz L, Radenbaugh A, Singh S, Swing V, Tissier C, Zhang P, Huala E (2008). The *Arabidopsis* information resource (TAIR): gene structure and function annotation. Nucleic Acids Res.

[CR55] Szczesniak MW, Rosikiewicz W, Makalowska I (2016). CANTATAdb: a collection of plant long non-coding RNAs. Plant Cell Physiol.

[CR56] Szczesniak MW, Bryzghalov O, Ciomborowska-Basheer J, Makalowska I (2019). CANTATAdb 2.0: expanding the collection of plant long noncoding RNAs. Methods Mol Biol.

[CR57] Uebler S, Marton ML, Dresselhaus T (2015). Classification of EA1-box proteins and new insights into their role during reproduction in grasses. Plant Reprod.

[CR58] Ulitsky I (2016). Evolution to the rescue: using comparative genomics to understand long non-coding RNAs. Nat Rev Genet.

[CR59] Wunderlich M, Gross-Hardt R, Schoffl F (2014). Heat shock factor HSFB2a involved in gametophyte development of *Arabidopsis thaliana* and its expression is controlled by a heat-inducible long non-coding antisense RNA. Plant Mol Biol.

[CR60] Xu Z, Hu L, Shi B, Geng S, Xu L, Wang D, Lu ZJ (2018). Ribosome elongating footprints denoised by wavelet transform comprehensively characterize dynamic cellular translation events. Nucleic Acids Res.

[CR61] Yu Y, Zhang Y, Chen X, Chen Y (2019). Plant noncoding RNAs: hidden players in development and stress responses. Annu Rev Cell Dev Biol.

[CR62] Yuan J, Li J, Yang Y, Tan C, Zhu Y, Hu L, Qi Y, Lu ZJ (2018). Stress-responsive regulation of long non-coding RNA polyadenylation in *Oryza sativa*. Plant J.

[CR63] Zinn KE, Tunc-Ozdemir M, Harper JF (2010). Temperature stress and plant sexual reproduction: uncovering the weakest links. J Exp Bot.

