# Supplementary material for: Characterization of novel pollen-expressed transcripts reveals their potential roles in pollen heat stress response in Arabidopsis thaliana
Source: Plant Reprod. 2021 Jan 18;34(1):61–78. doi: 10.1007/s00497-020-00400-1 (PMC7902599; doi:10.1007/s00497-020-00400-1)

Doc S2: Periodicity plots

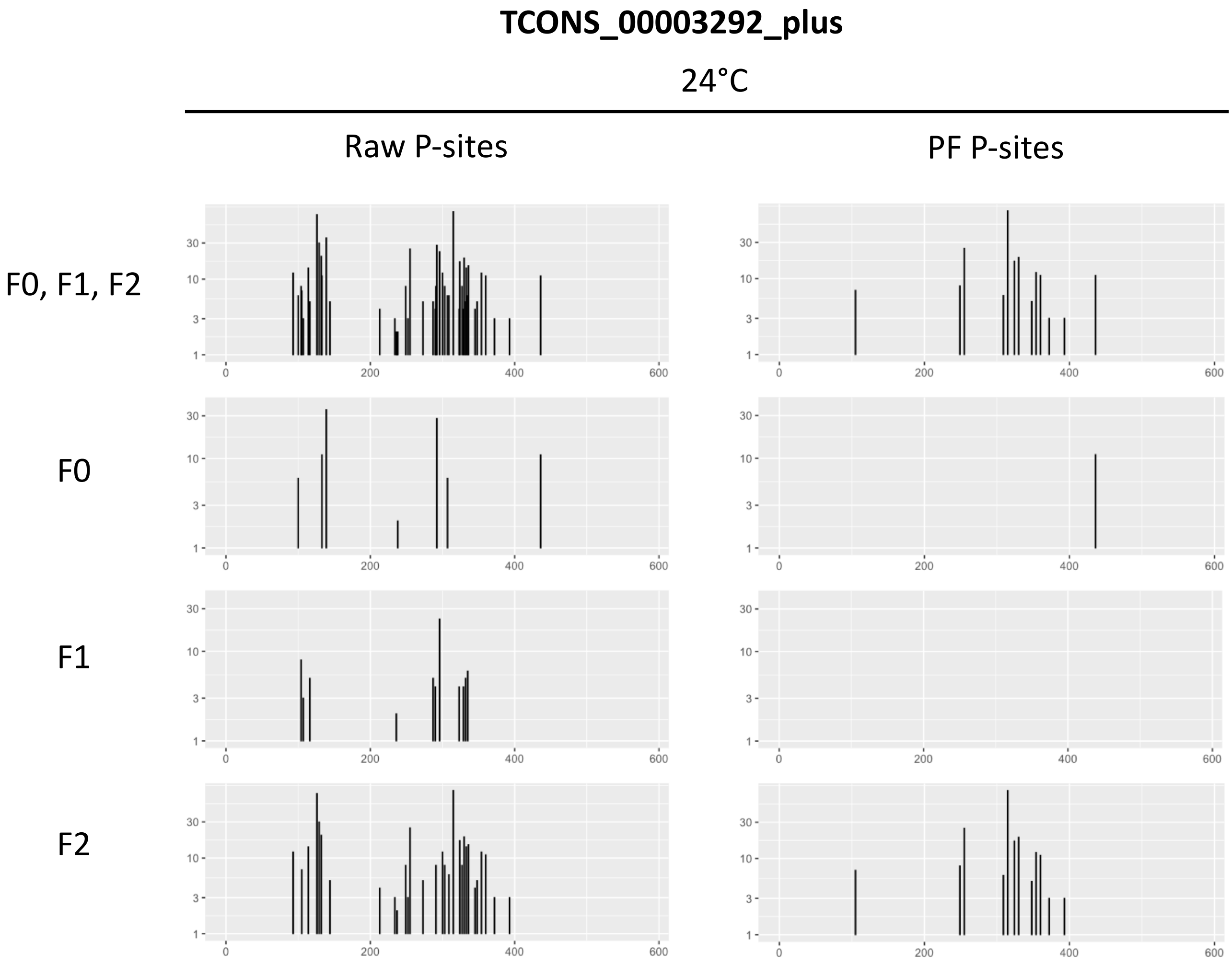

TCONS_00013674_plus

24°C

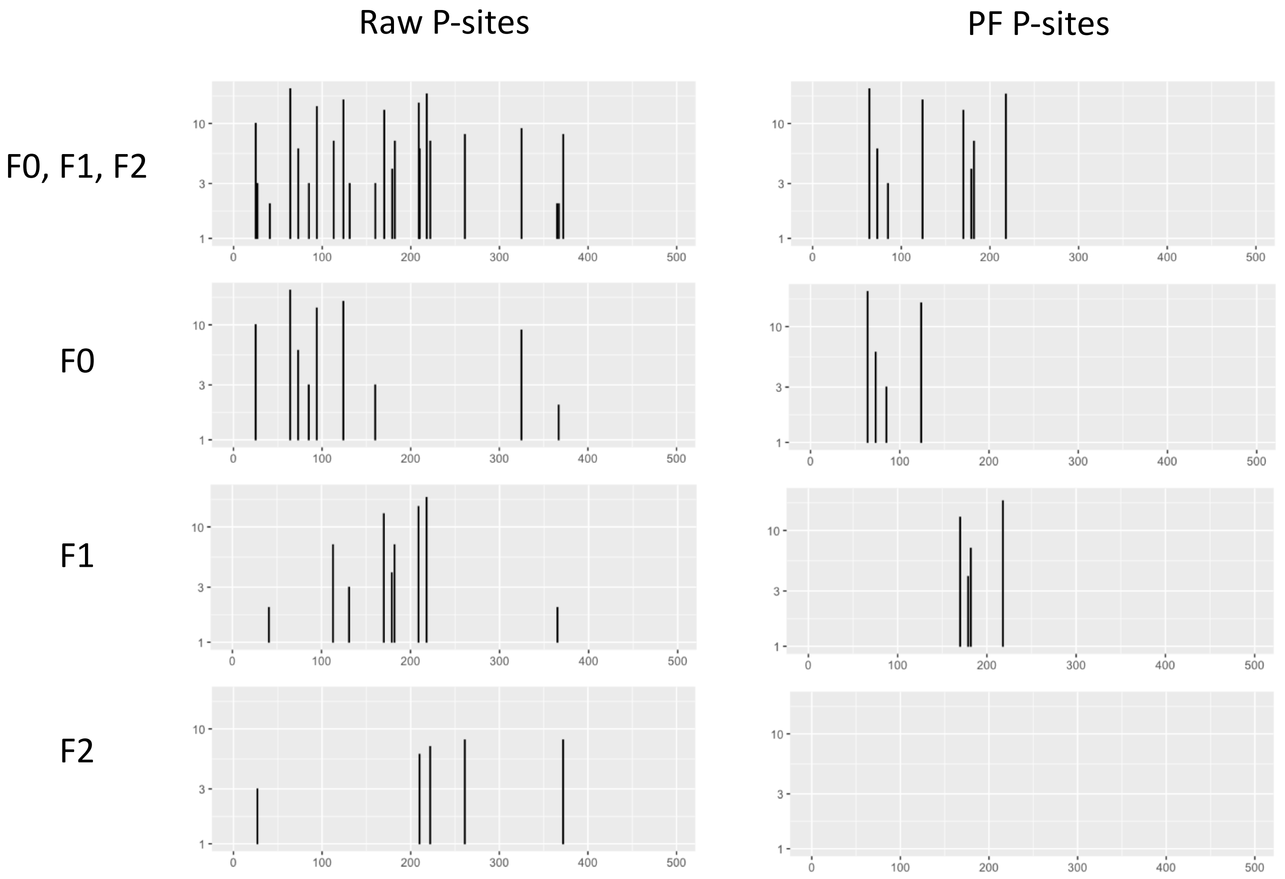

TCONS_00014294_plus

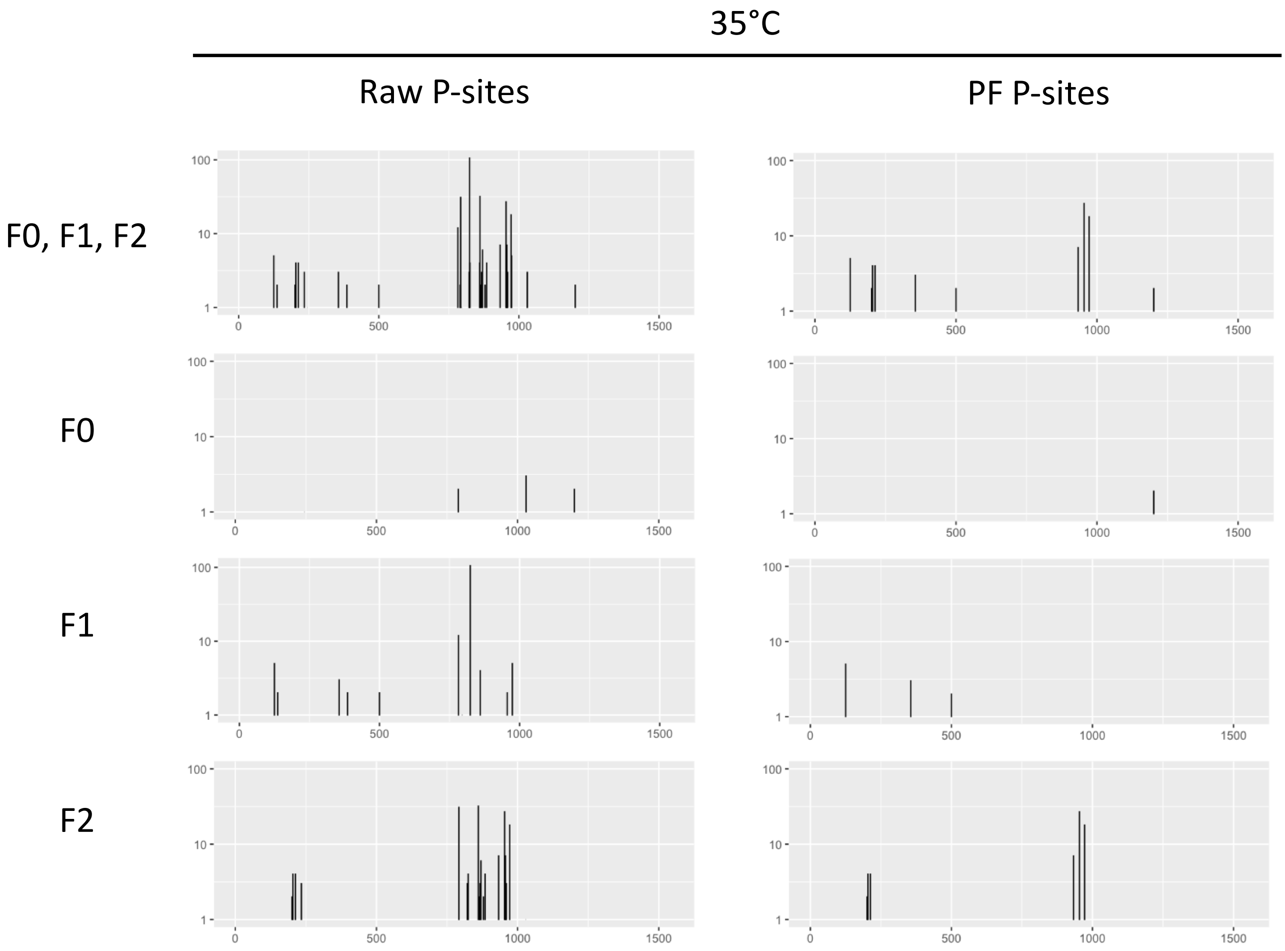

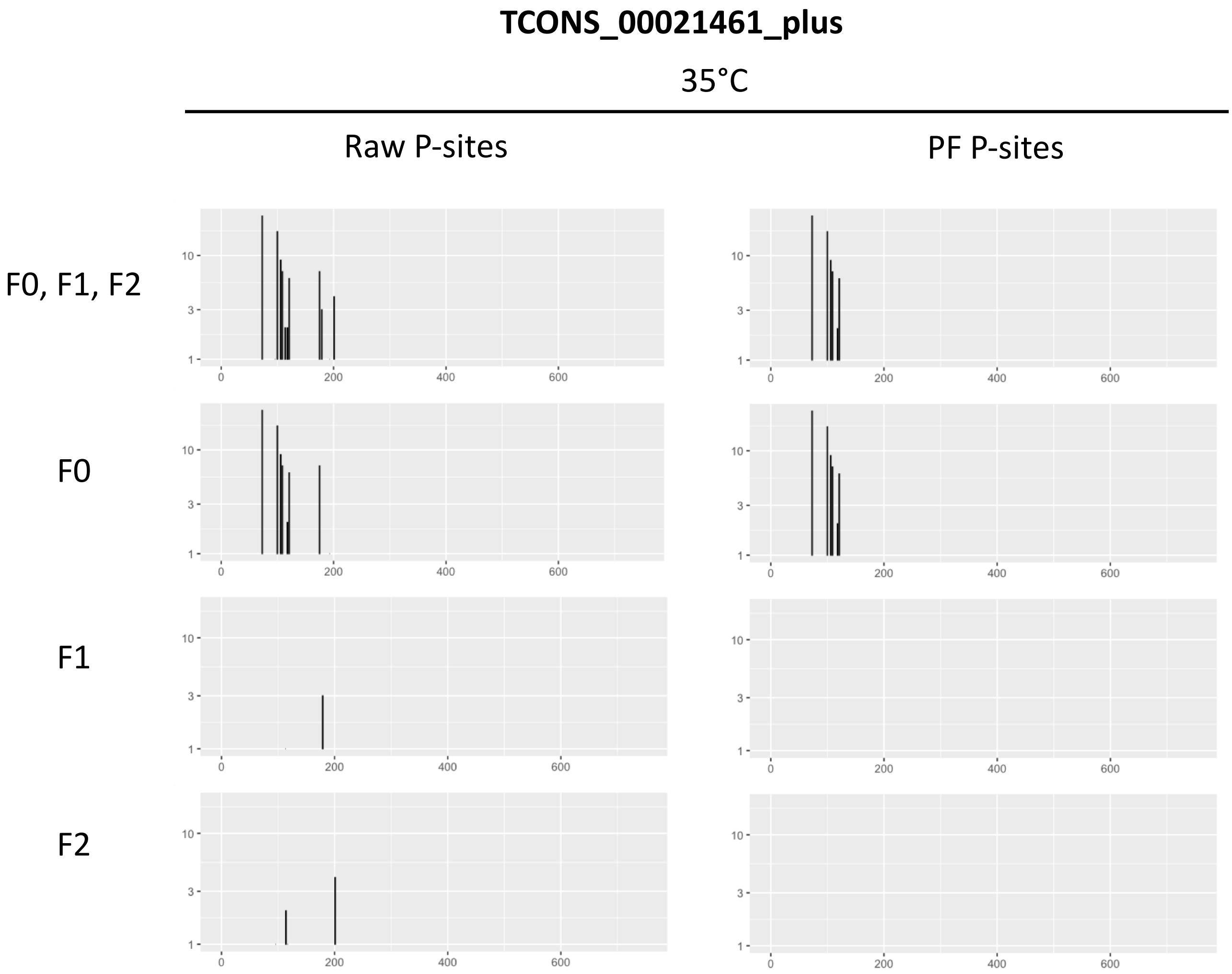
TCONS_00021461_plus
35°C
Raw P-sites
PF P-sites
F0, F1, F2
F0
F1
F2
10
3
1
0
200
400
600

TCONS_00024073_plus

35°C

Raw P-sites | PF P-sites

Rows: F0, F1, F2 / F0 / F1 / F2

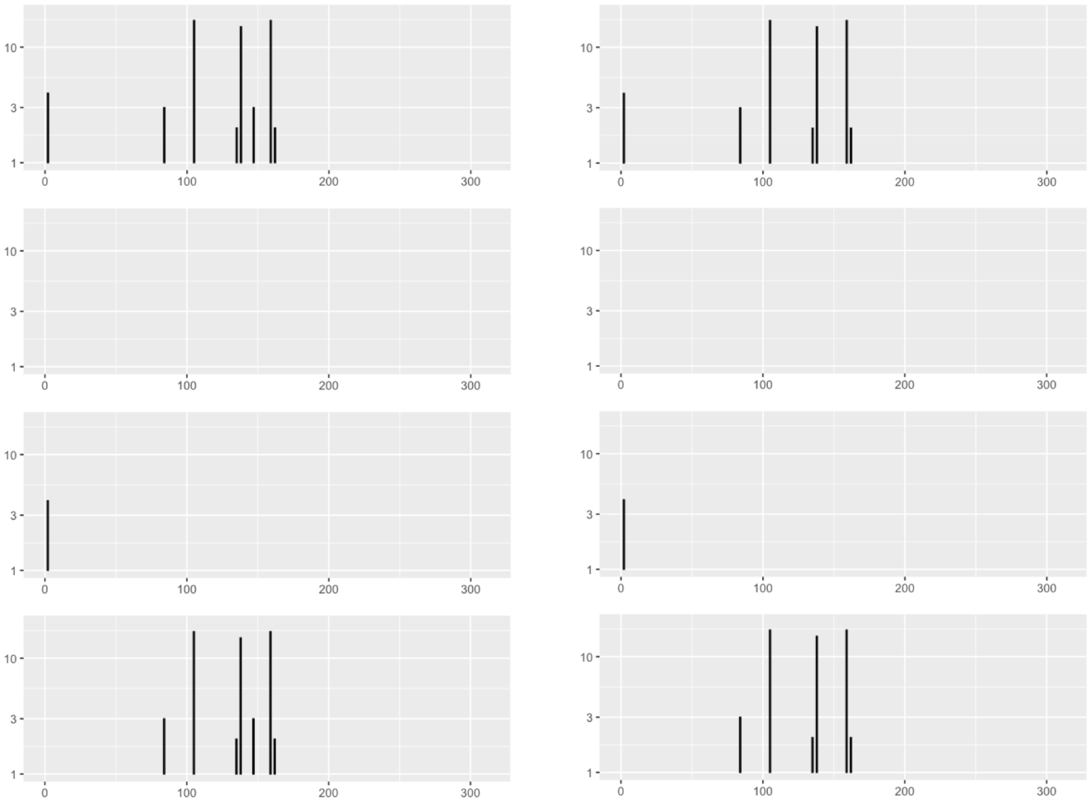

TCONS_00026848_plus

24°C

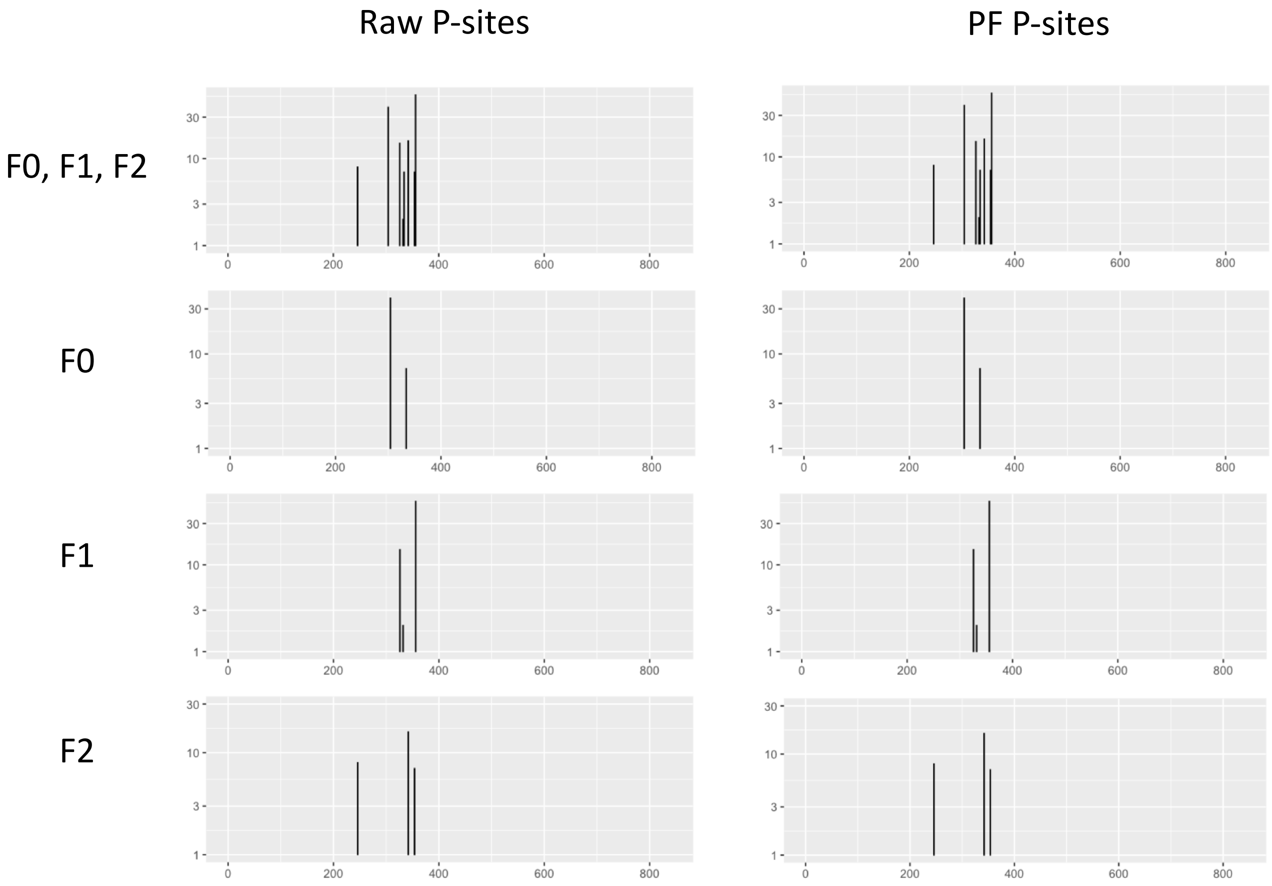

TCONS_00029361_plus

24°C

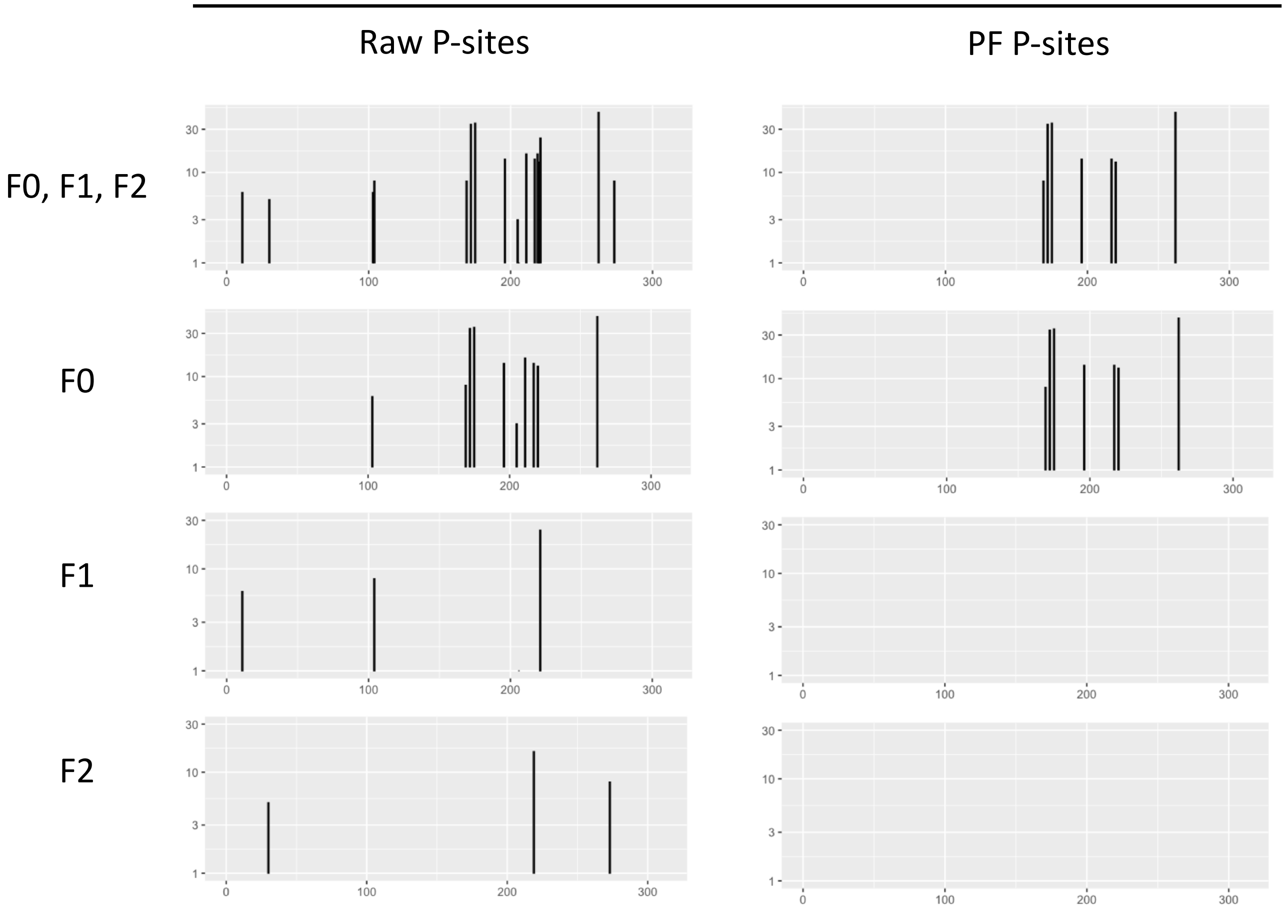

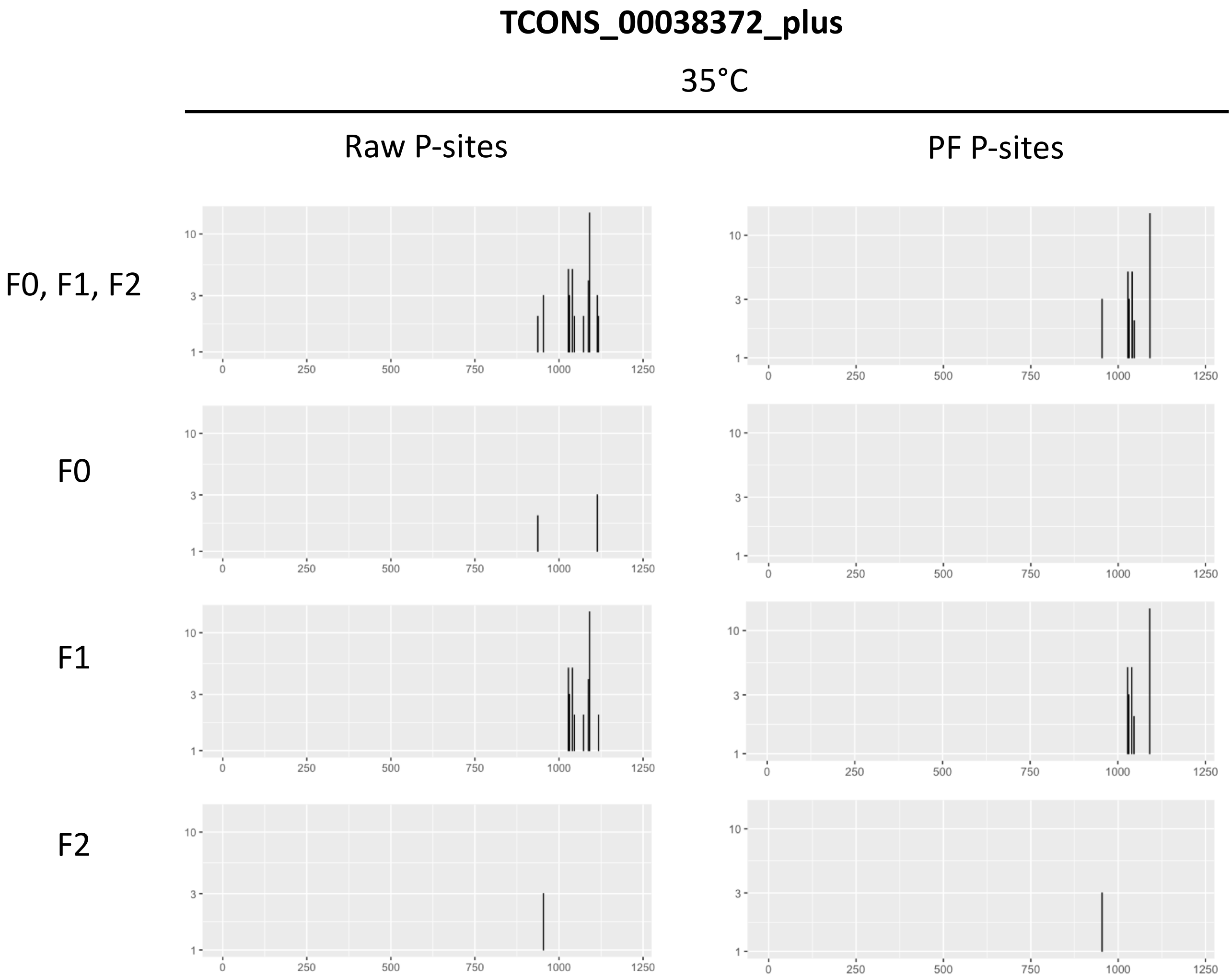
TCONS_00038372_plus
35°C
Raw P-sites
PF P-sites
F0, F1, F2
F0
F1
F2

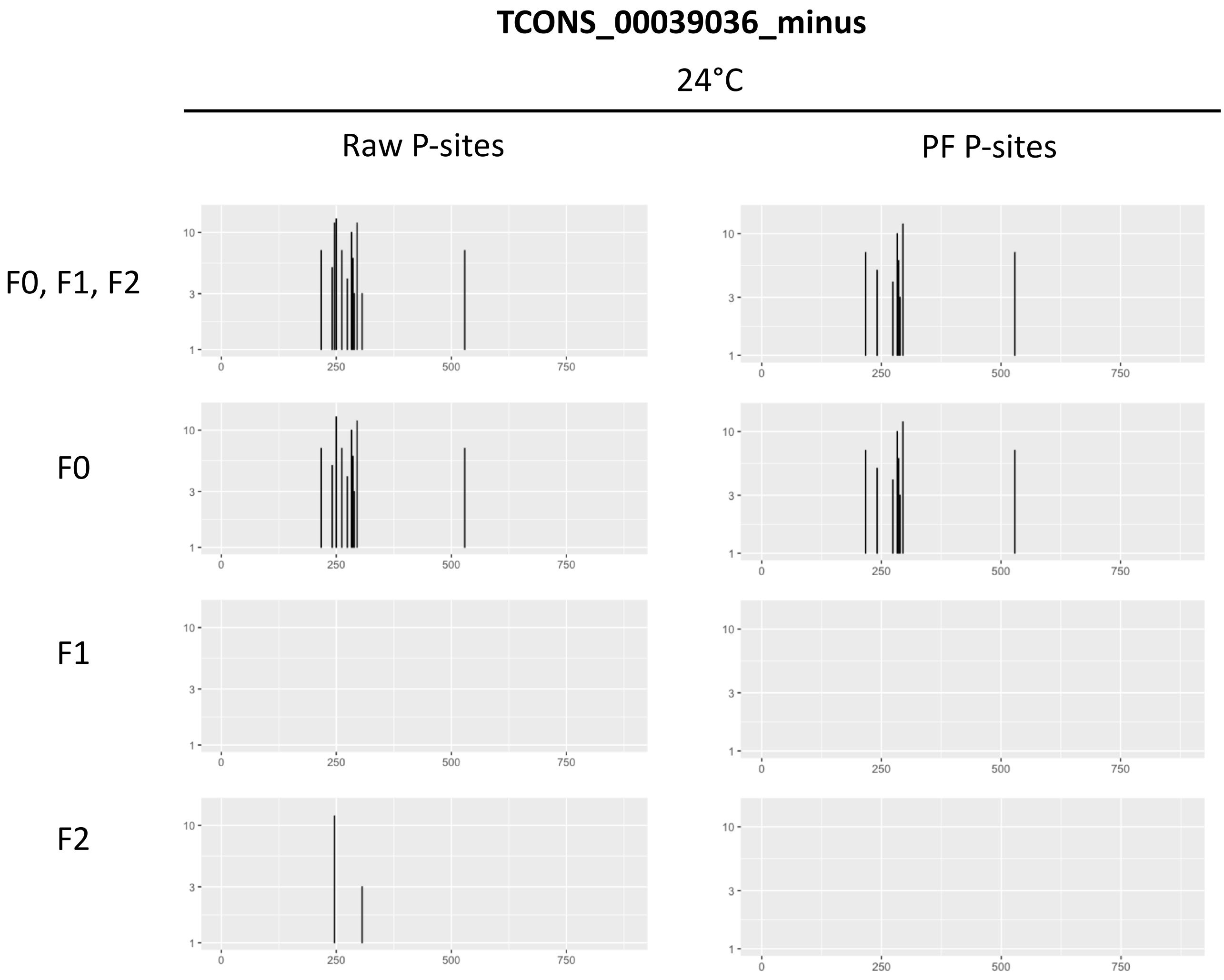

TCONS_00039036_minus
24°C
Raw P-sites
PF P-sites
F0, F1, F2
F0
F1
F2
10
3
1
0
250
500
750

TCONS_00042679_plus

24°C

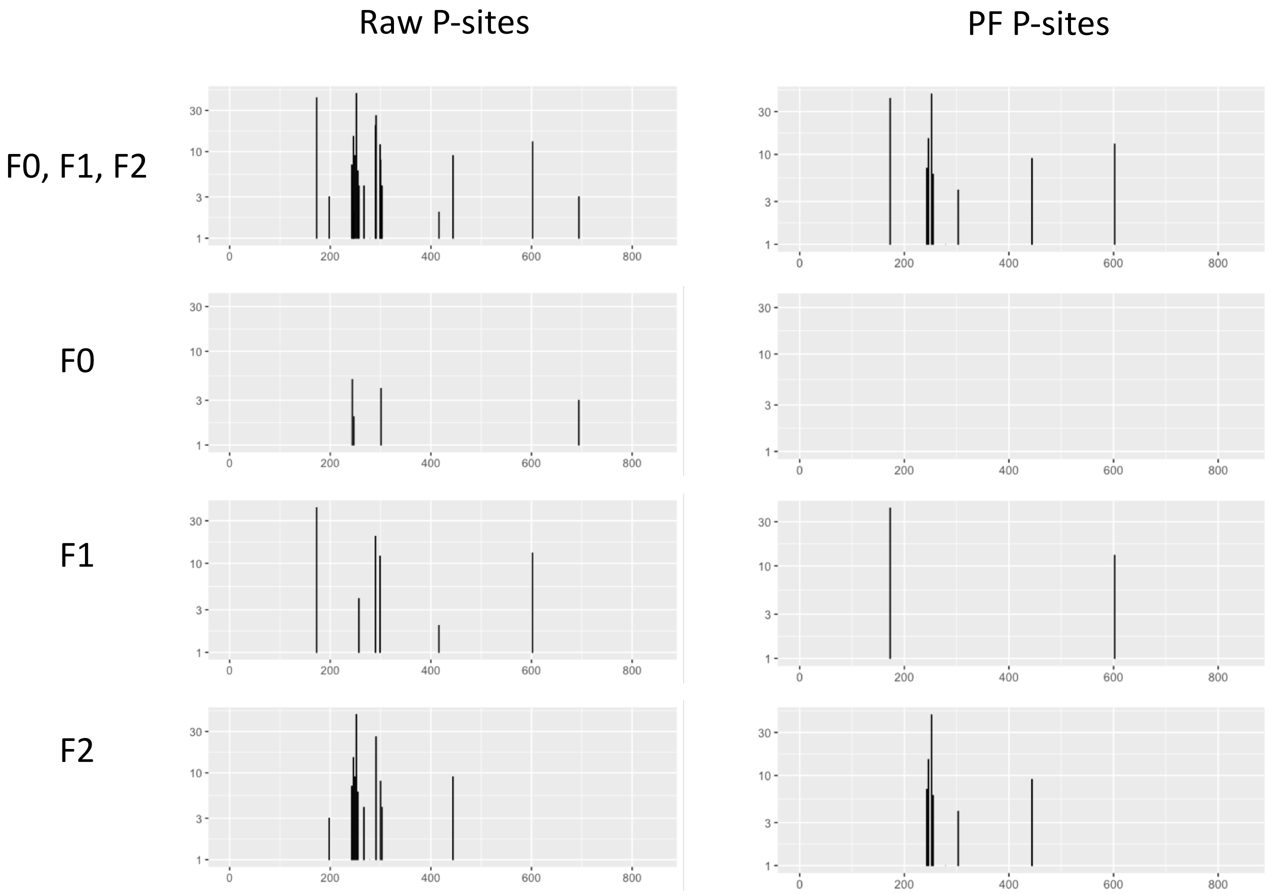

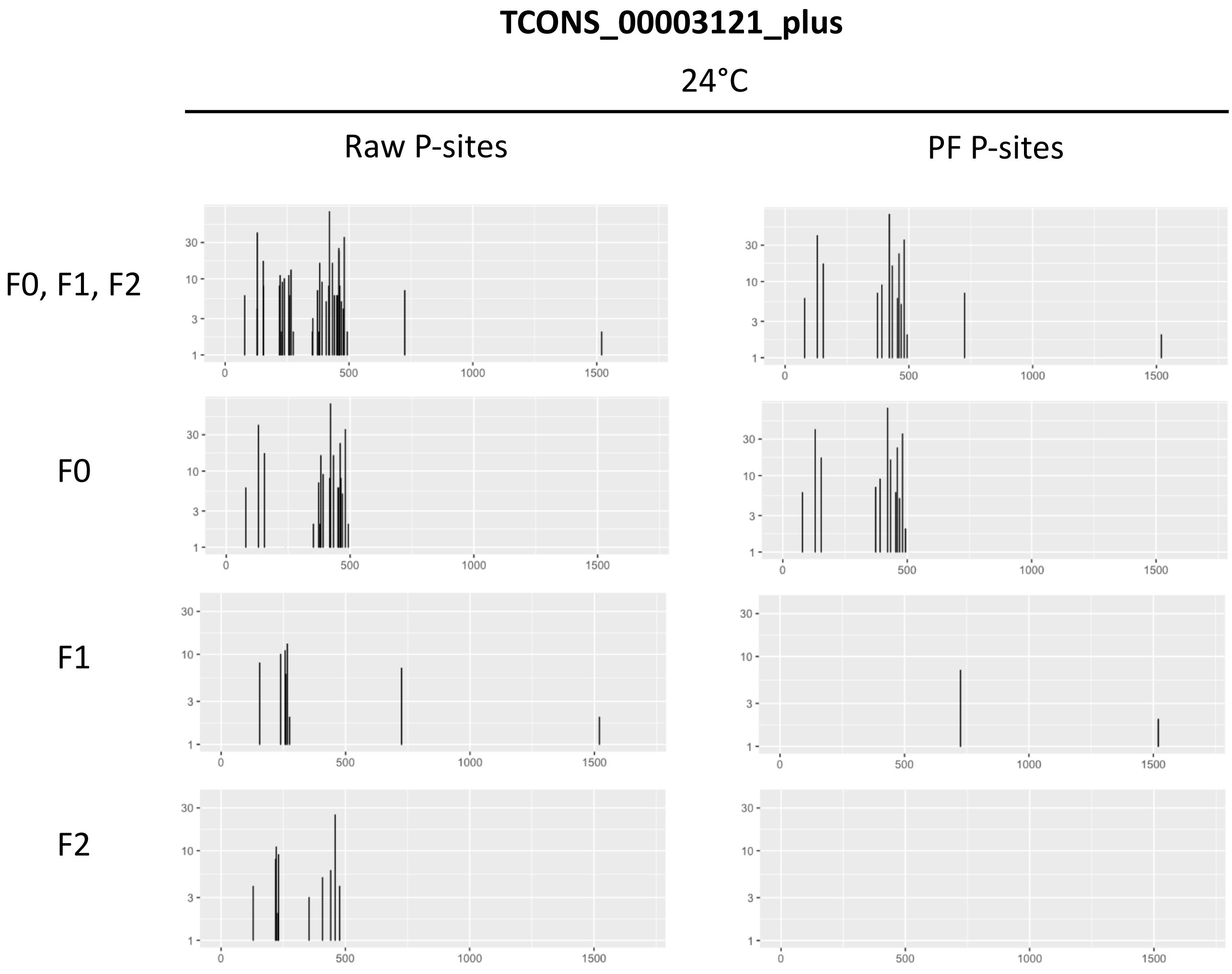
TCONS_00003121_plus
24°C
Raw P-sites
PF P-sites
F0, F1, F2
F0
F1
F2
30
10
3
1
0
500
1000
1500

TCONS_00009618_minus

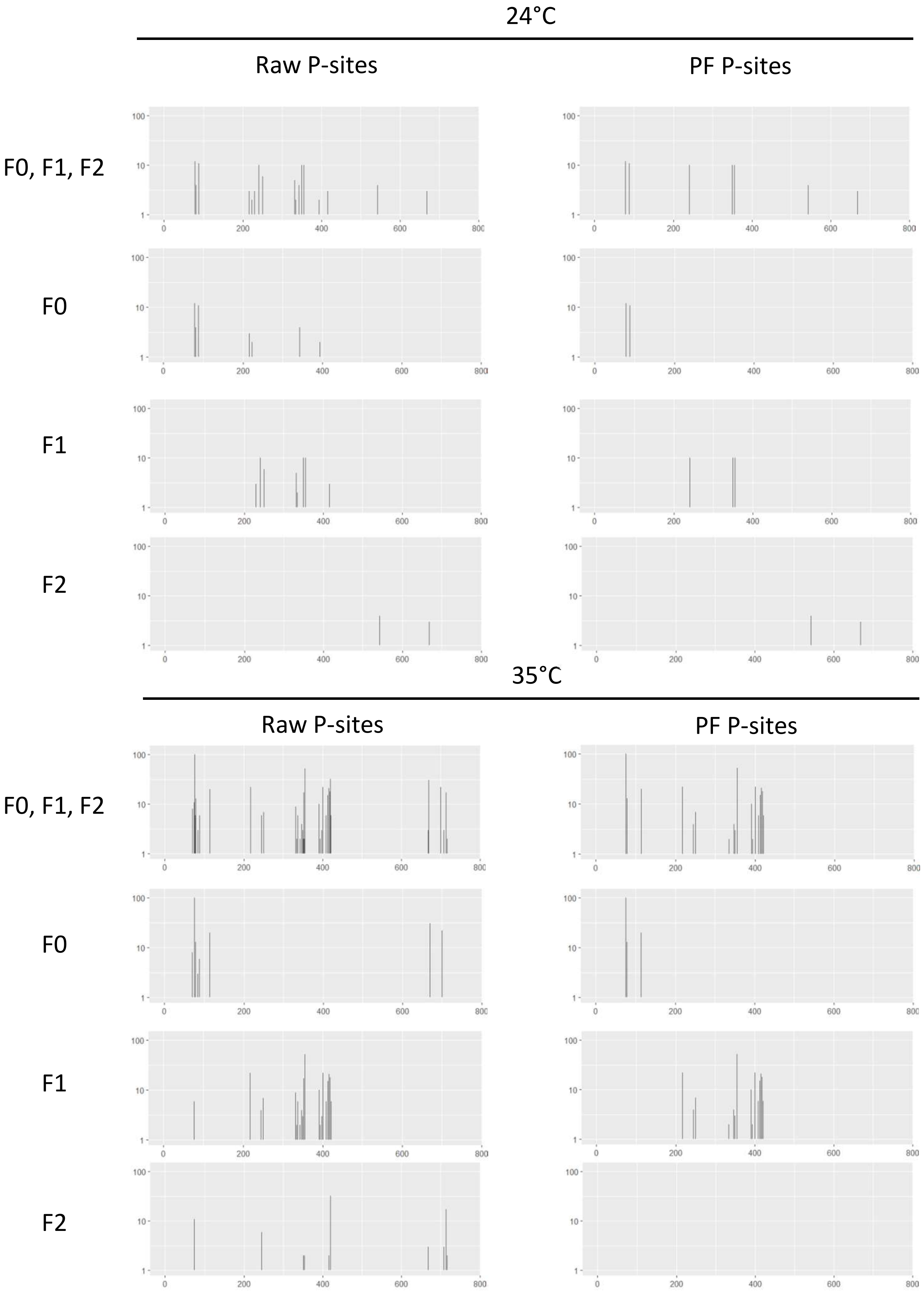

TCONS_00012928_minus

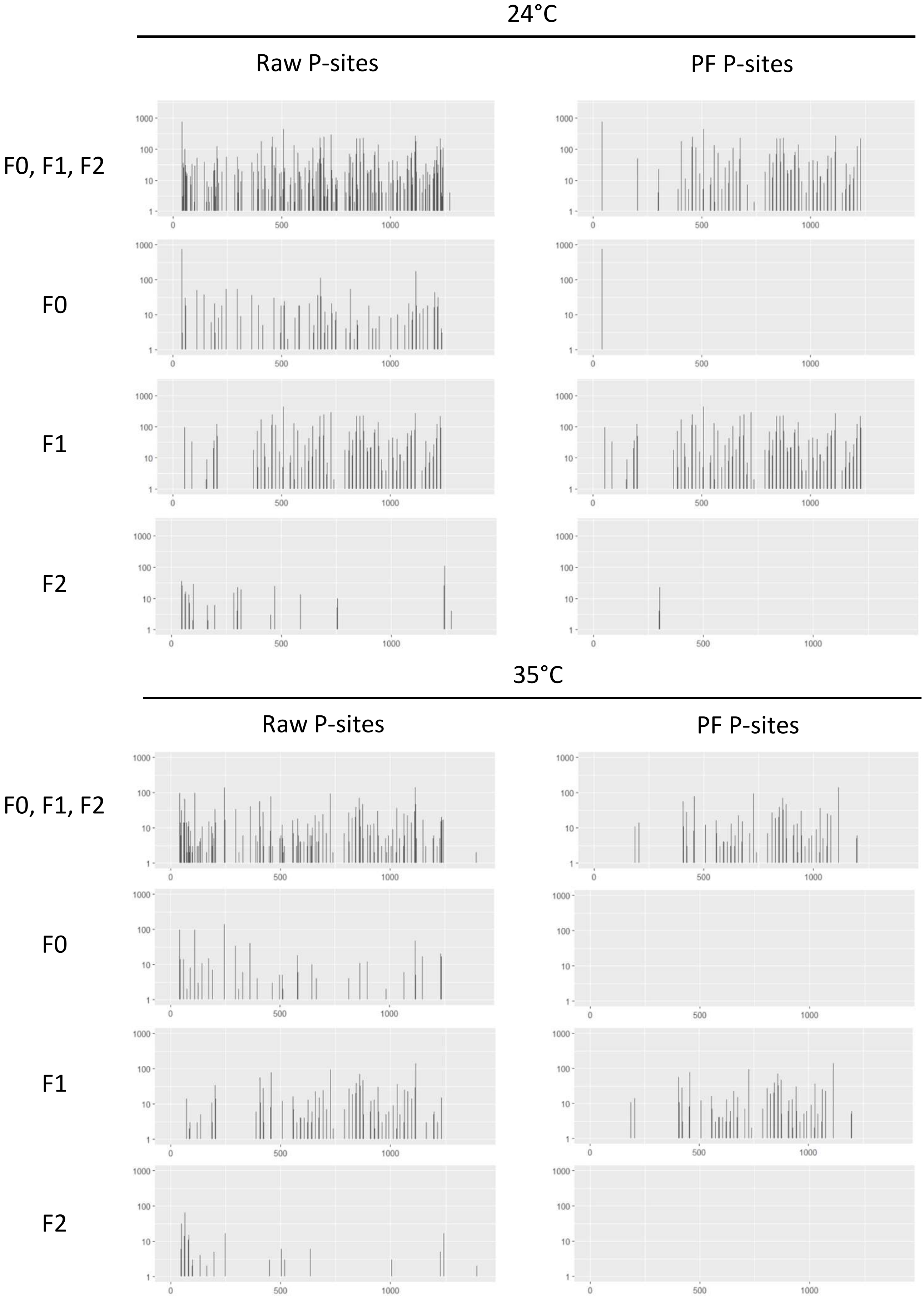

TCONS_00013972_plus

24°C

Raw P-sites

PF P-sites

F0, F1, F2

F0

F1

F2

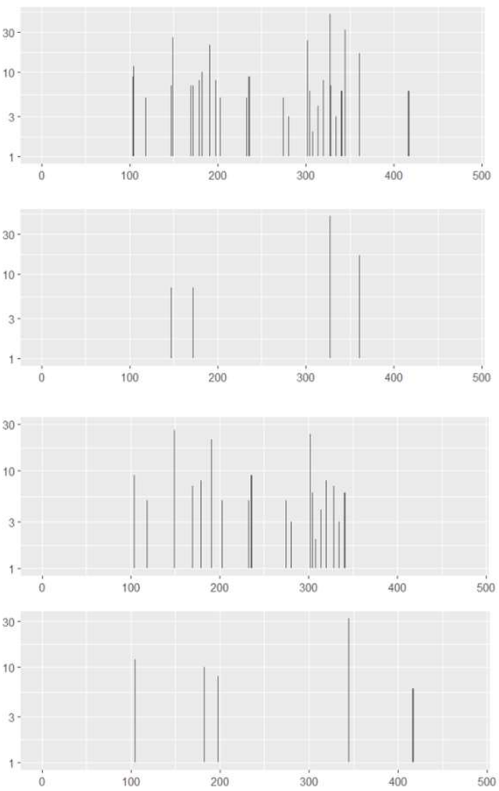

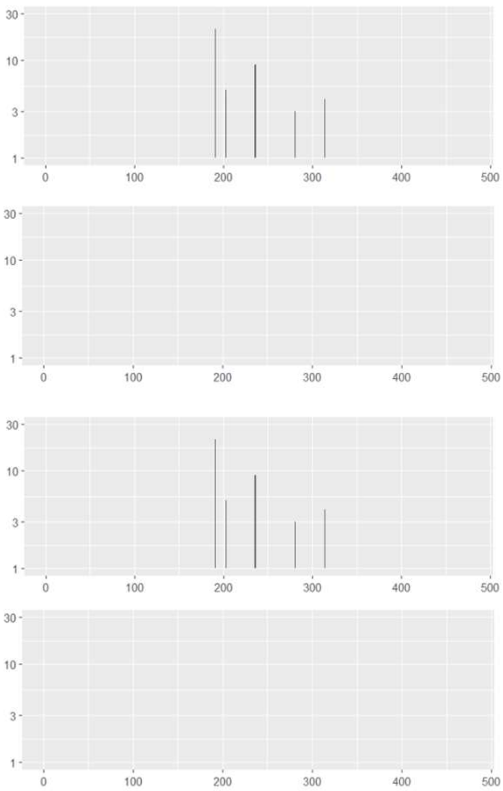

35°C

Raw P-sites

PF P-sites

F0, F1, F2

F0

F1

F2

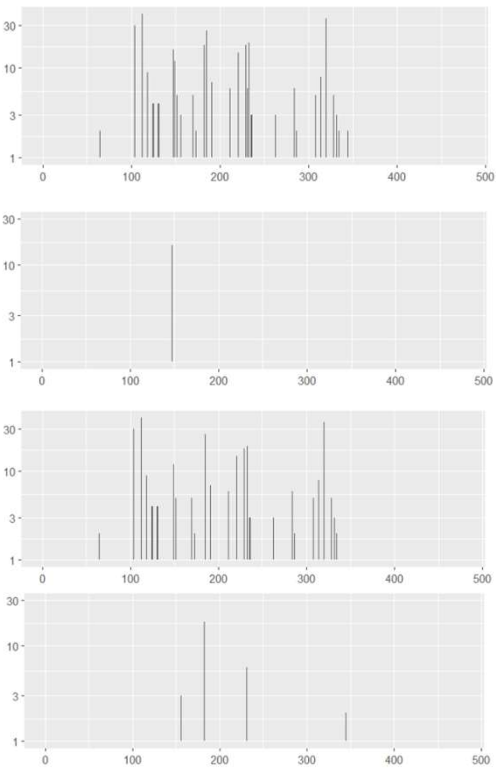

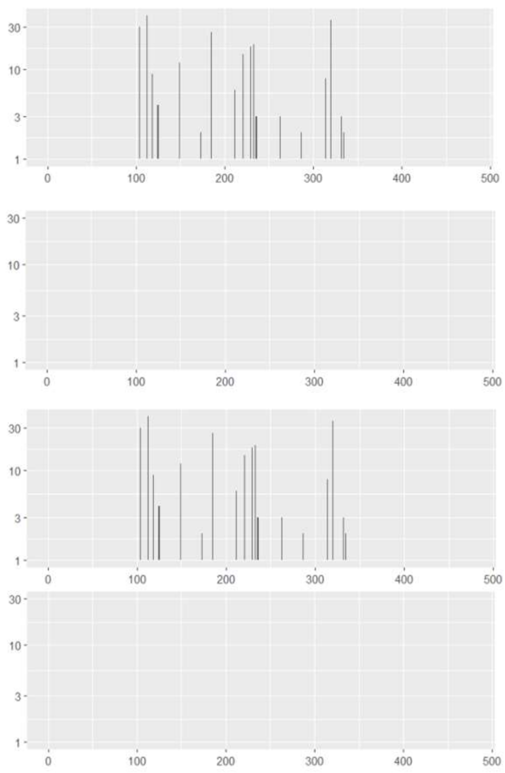

TCONS_00021207_minus

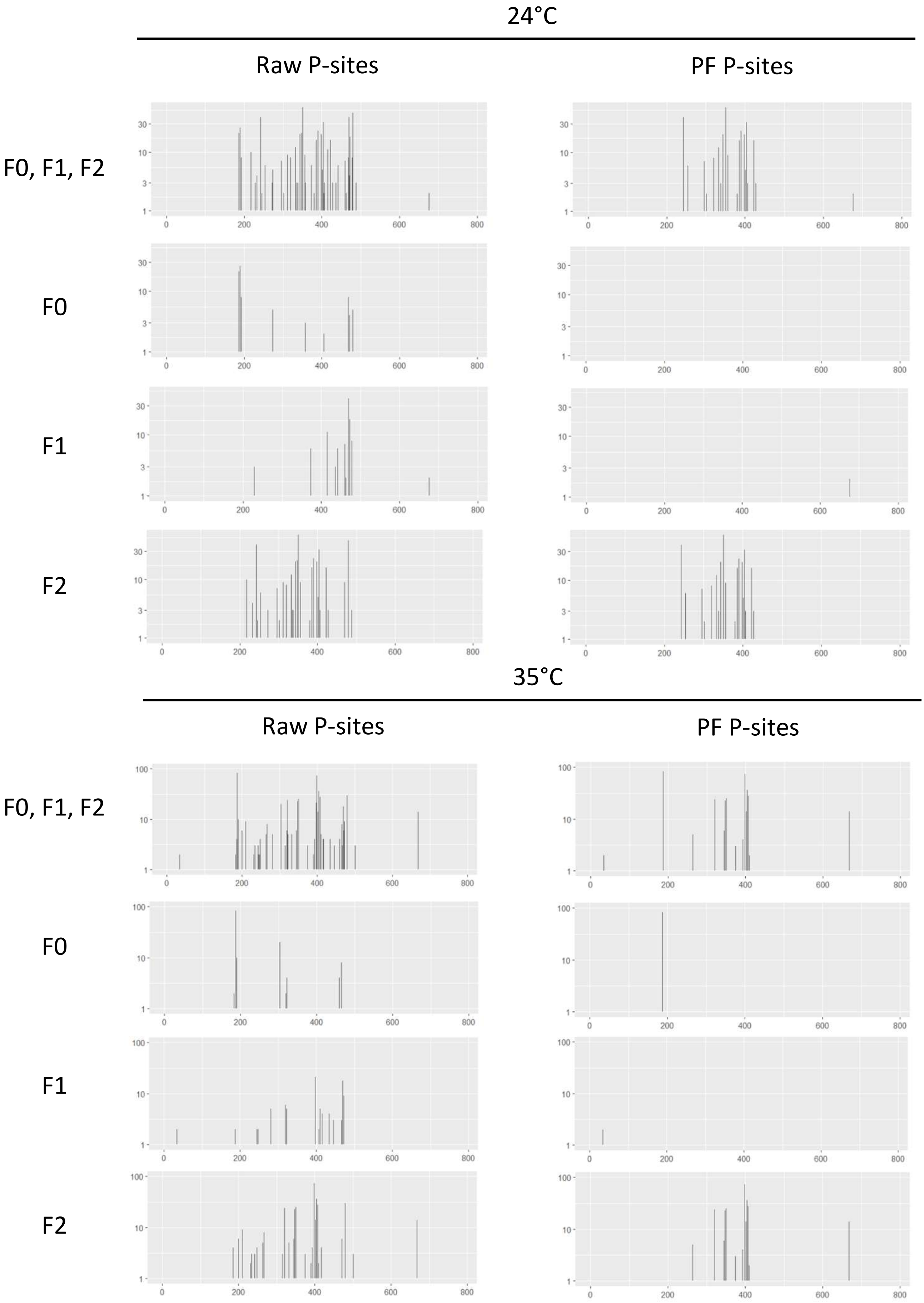

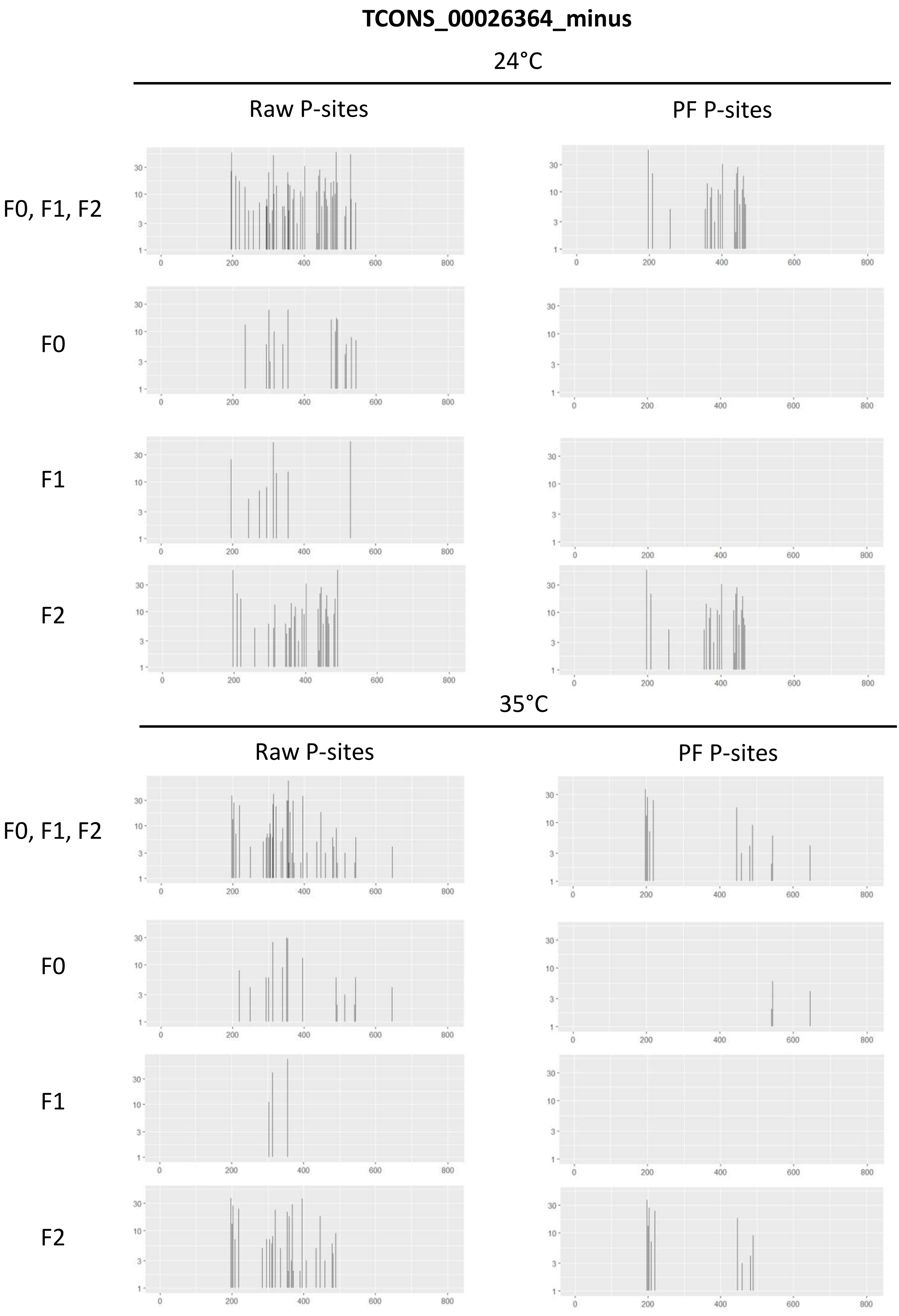

TCONS_00026364_minus
24°C
Raw P-sites
PF P-sites
F0, F1, F2
F0
F1
F2
35°C
Raw P-sites
PF P-sites
F0, F1, F2
F0
F1
F2
1
3
10
30
0
200
400
600
800

TCONS_00031274_plus

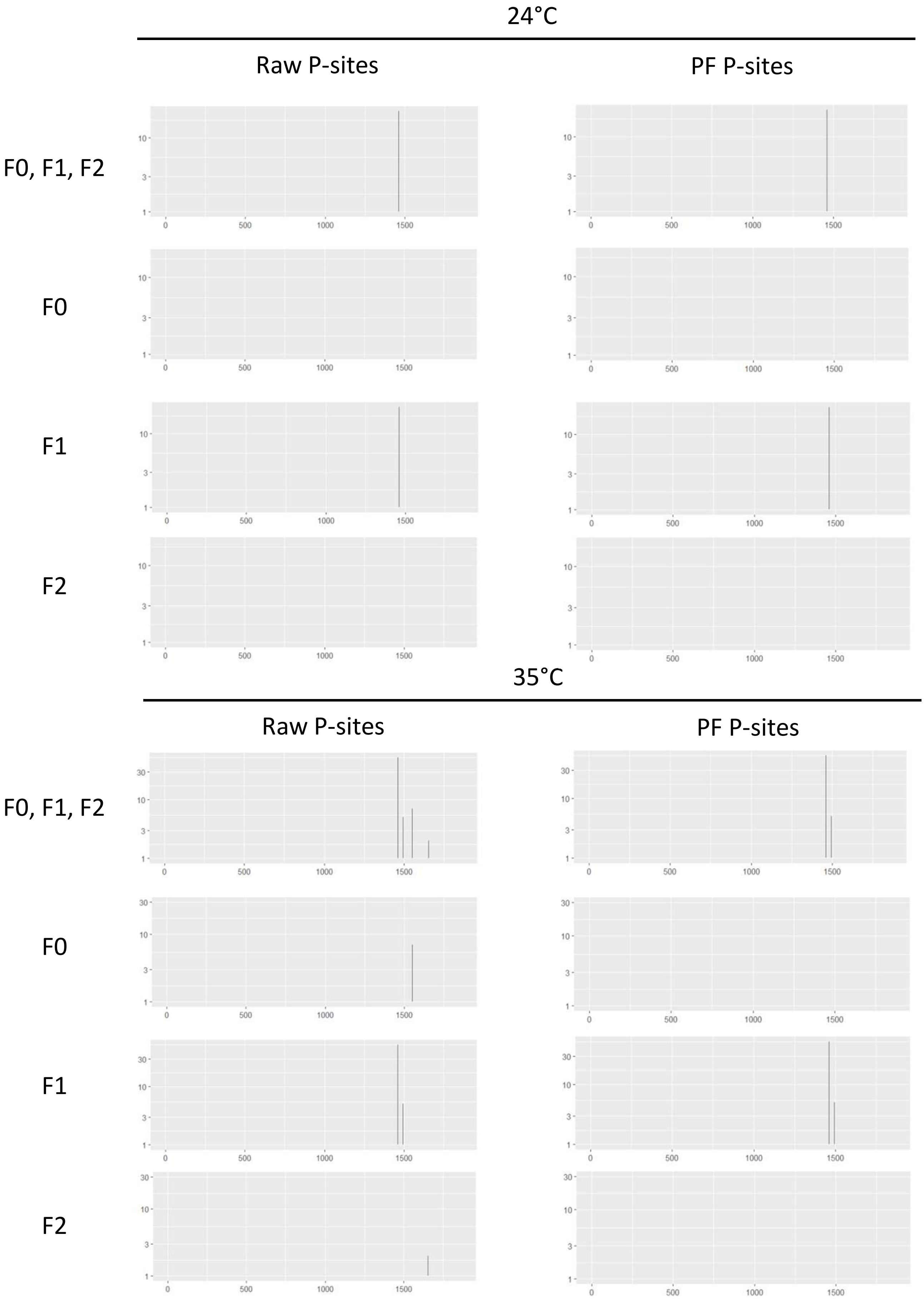

TCONS_00032246_minus

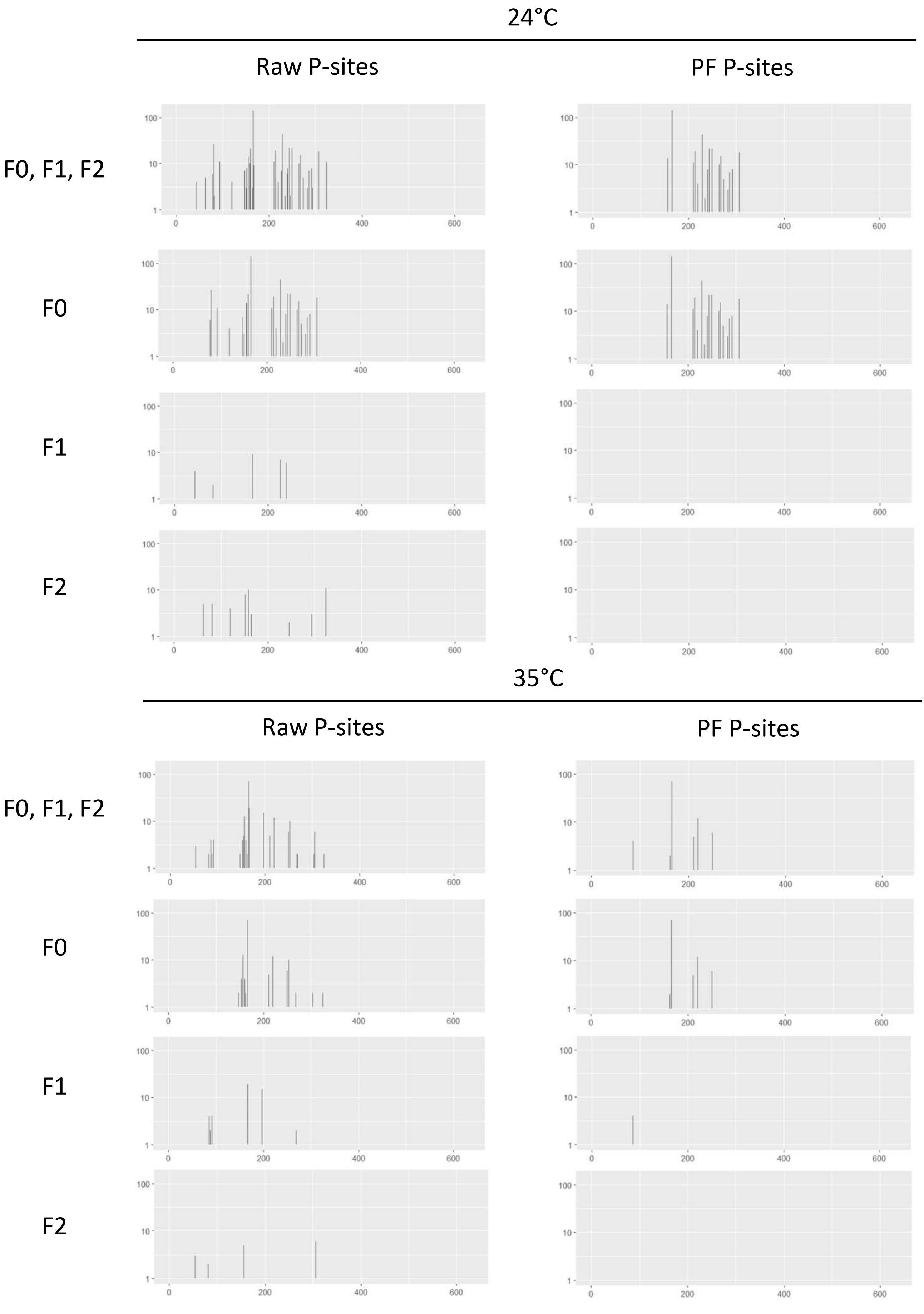

TCONS_00035135_plus

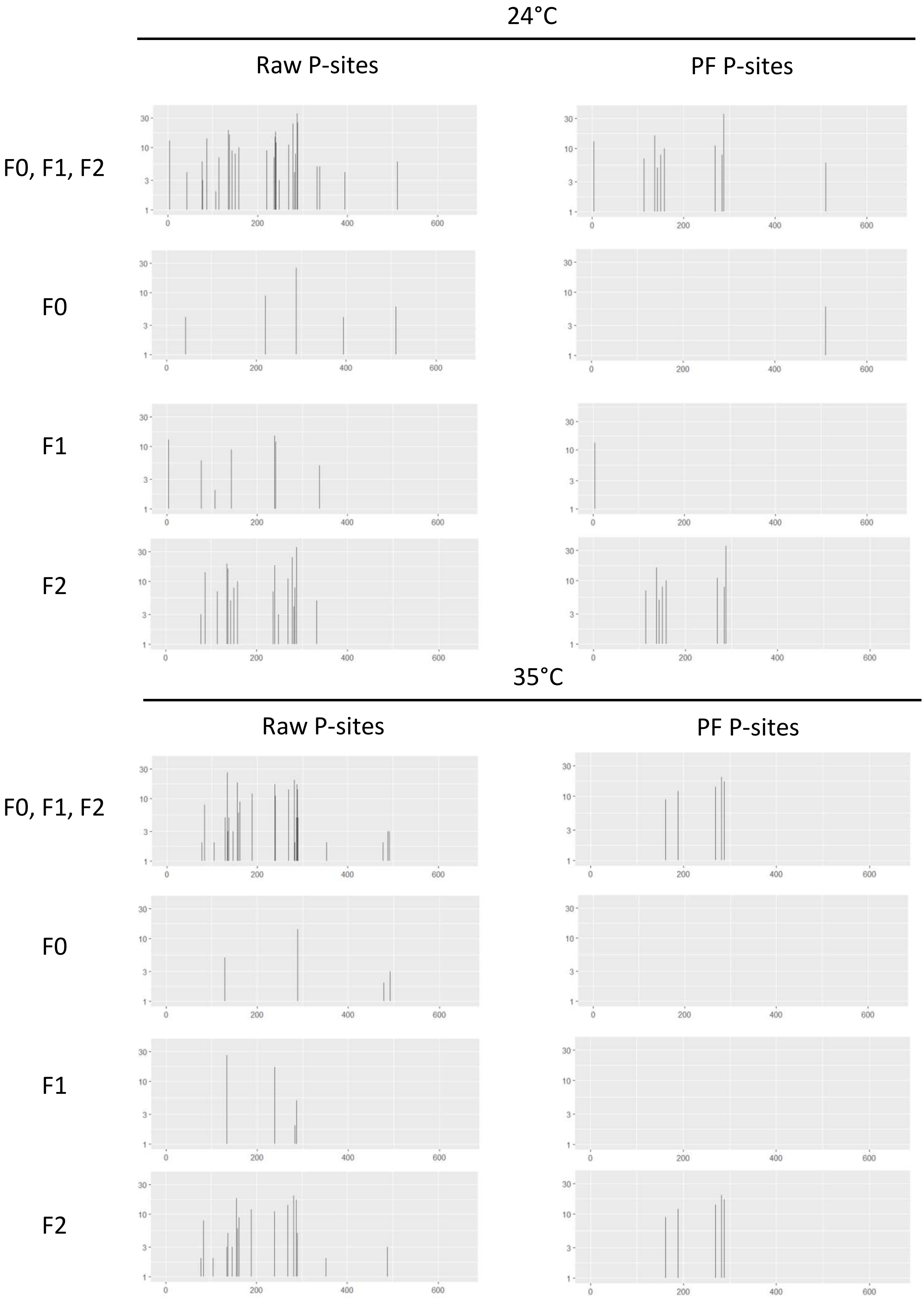

TCONS_00047195_minus

24°C

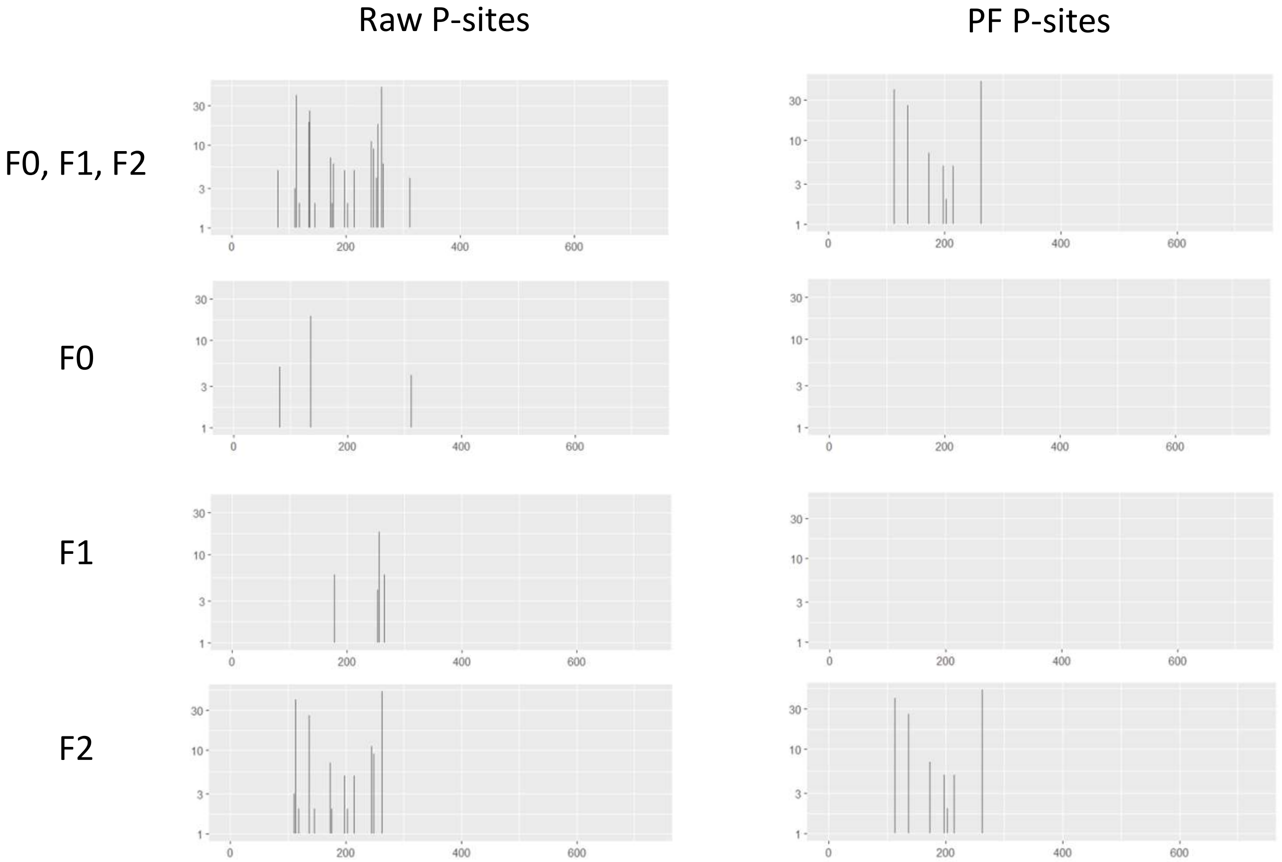

35°C

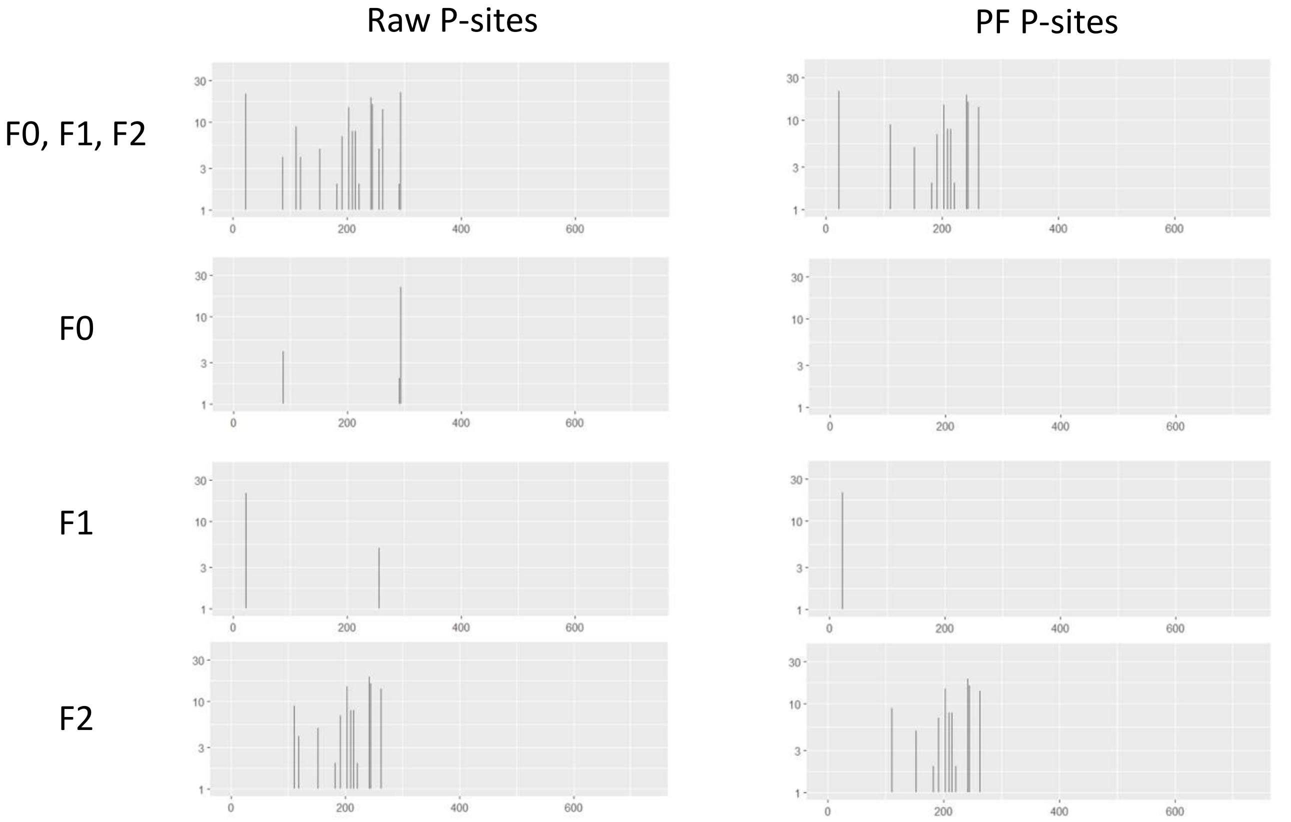

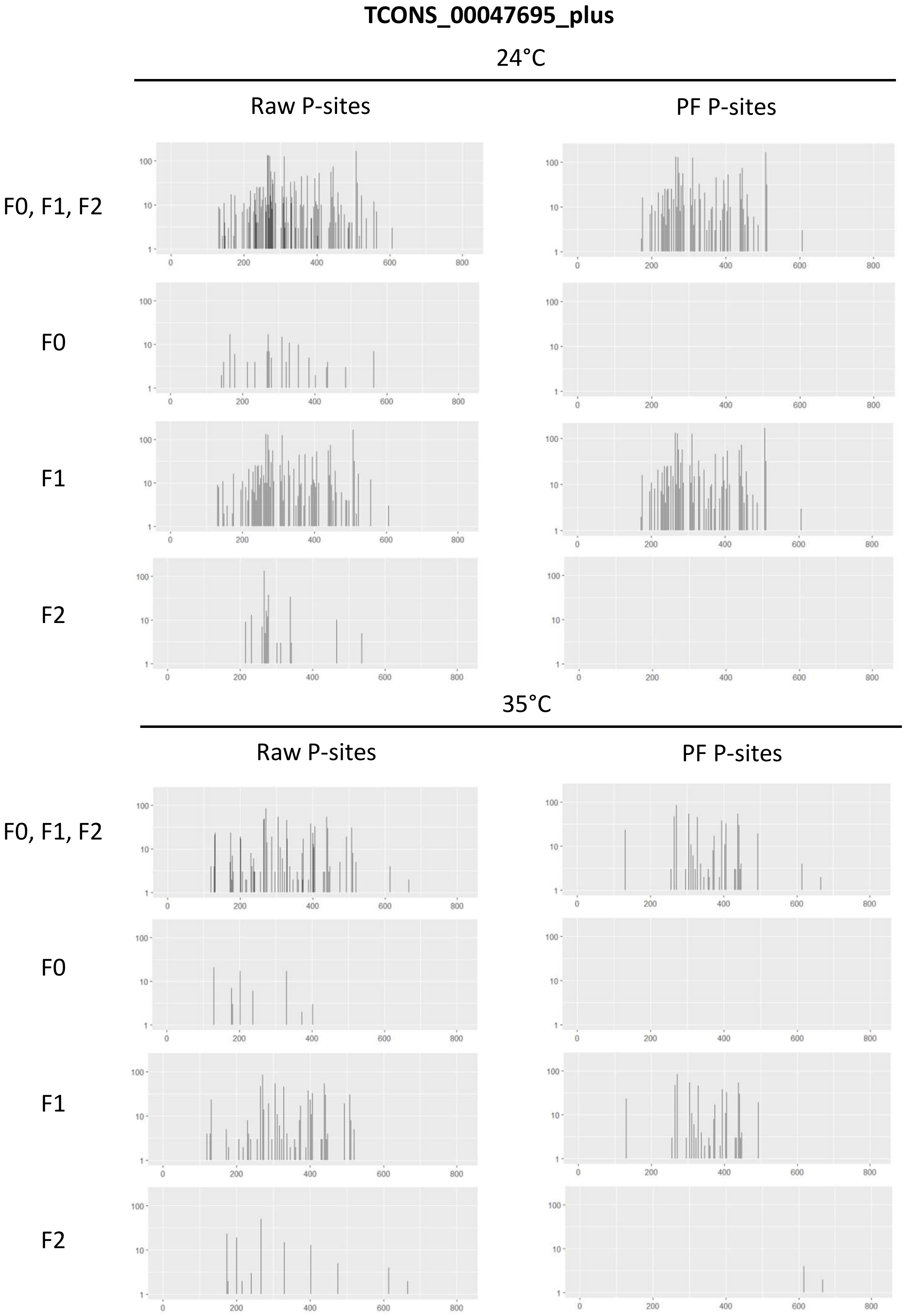
TCONS_00047695_plus
24°C
Raw P-sites
PF P-sites
F0, F1, F2
F0
F1
F2
35°C
Raw P-sites
PF P-sites
F0, F1, F2
F0
F1
F2
100
10
1
0
200
400
600
800

TCONS_00048440_minus

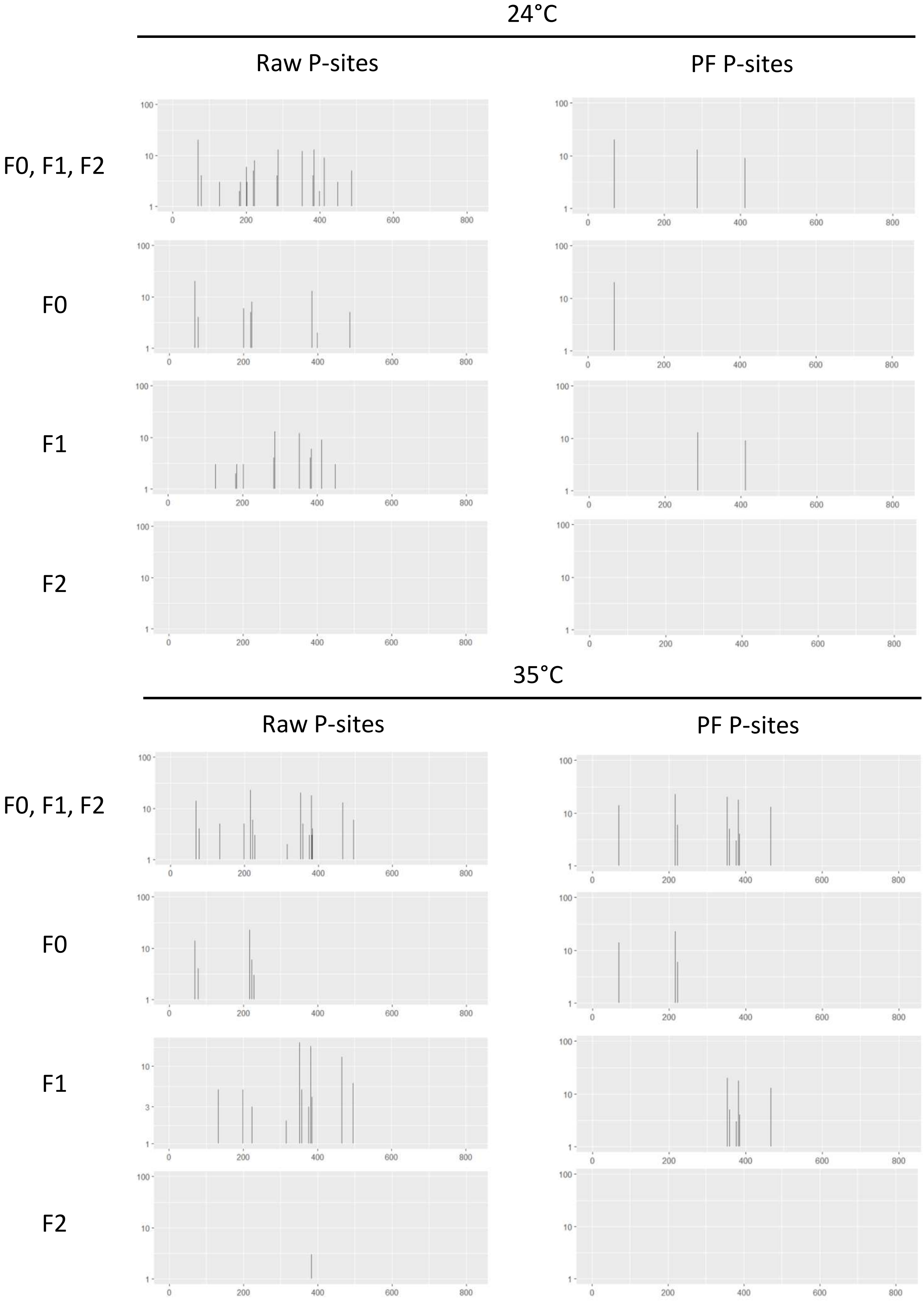

TCONS_00050361_plus

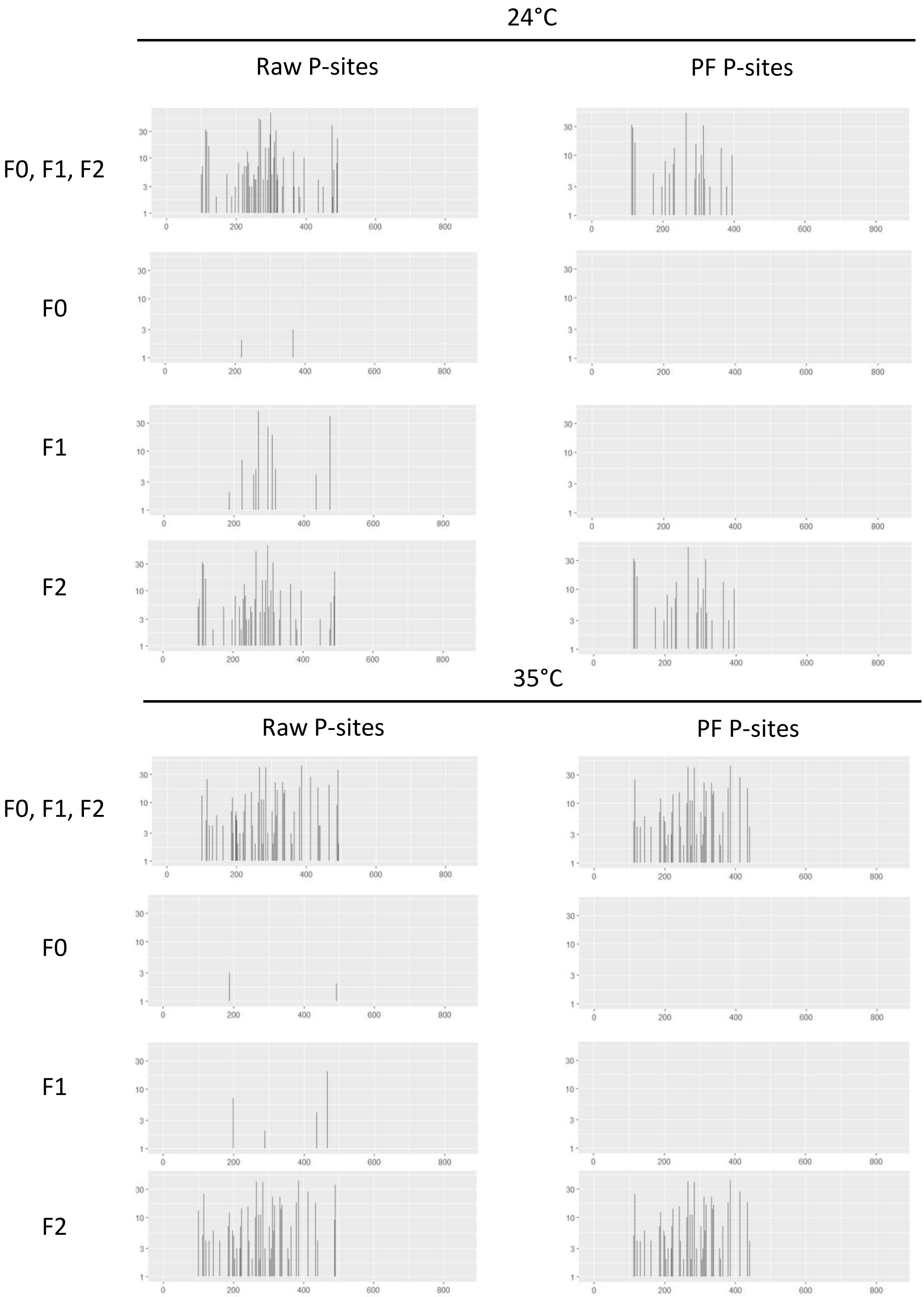

TCONS_00050361_plus

24°C

Raw P-sites | PF P-sites

F0, F1, F2

F0

F1

F2

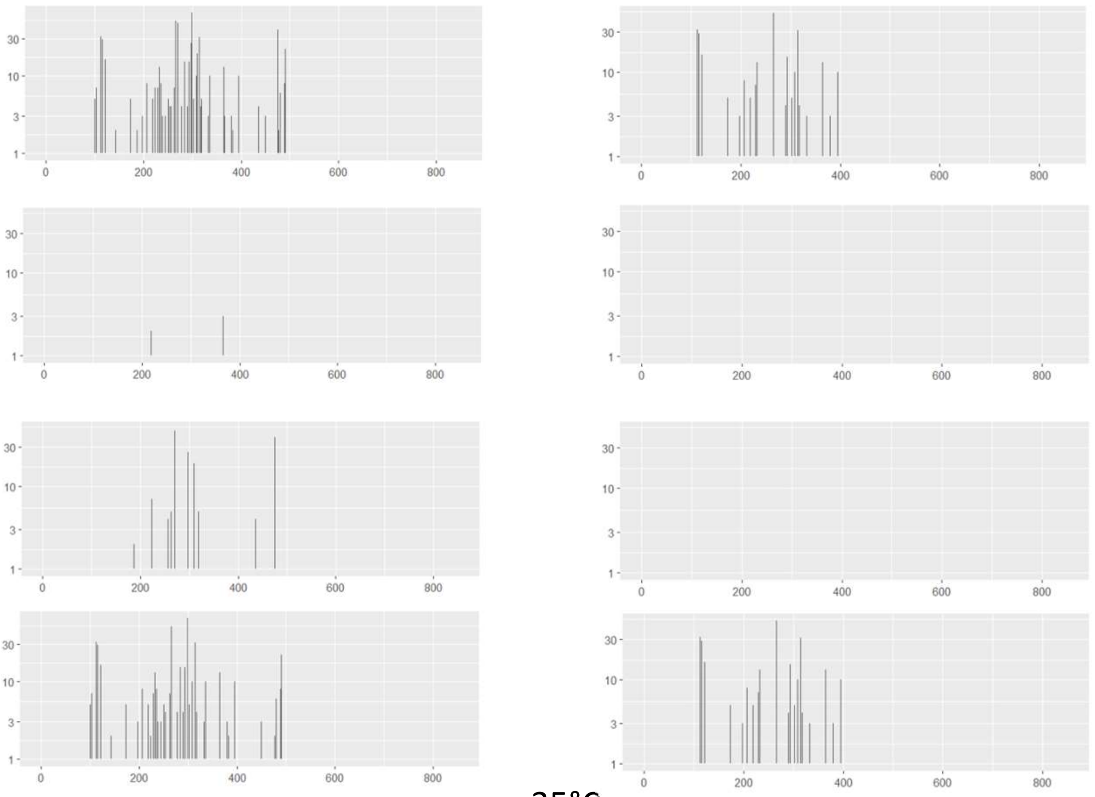

35°C

Raw P-sites | PF P-sites

F0, F1, F2

F0

F1

F2

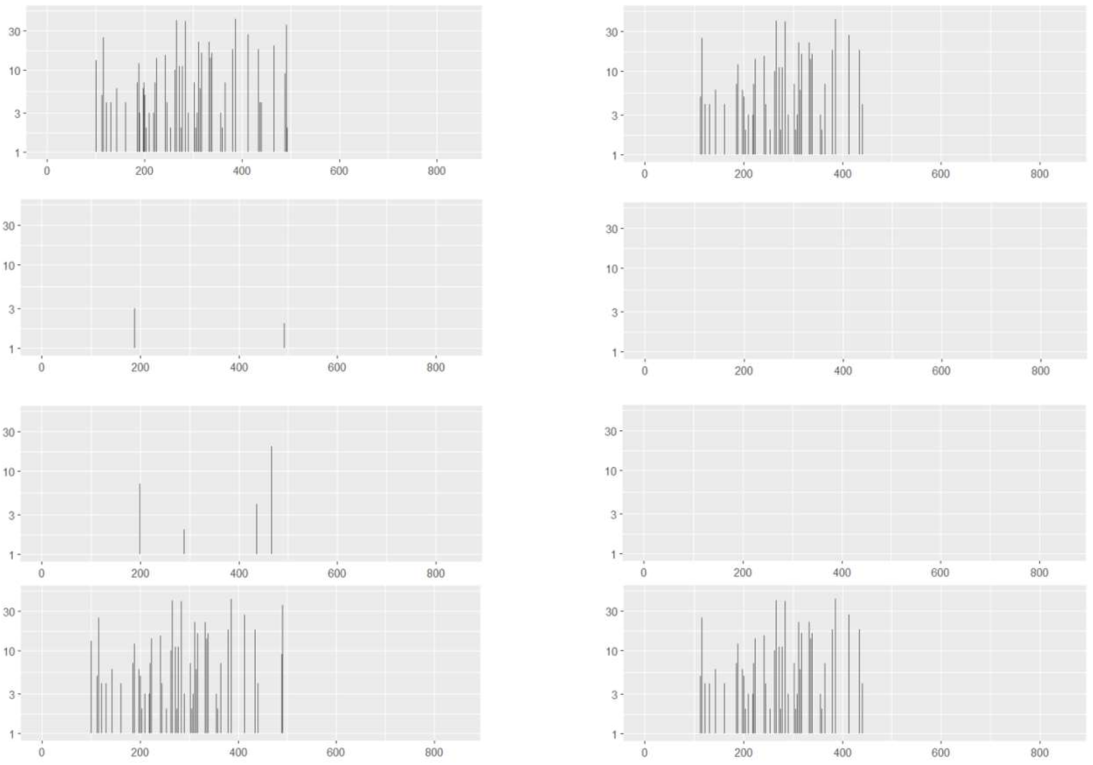

Supplement: Supplementary file 2 — Supplementary file1 (PDF 8293 kb) [file 497_2020_400_MOESM2_ESM.pdf]
