# Supplementary material for: Characterization of novel pollen-expressed transcripts reveals their potential roles in pollen heat stress response in Arabidopsis thaliana
Source: Plant Reprod. 2021 Jan 18;34(1):61–78. doi: 10.1007/s00497-020-00400-1 (PMC7902599; doi:10.1007/s00497-020-00400-1)
Supplement: Supplementary file 3 — Supplementary file1 (PDF 50 kb) [file 497_2020_400_MOESM3_ESM.pdf]

Doc S3

XLOC_030751 genomic fragment

CTATGTTTCTTTTTTTTTTTAGTTTTCCTATTTATTTAAGAGAAAAGTAAATATTTGTTGACACATGTCAACATCTGAACGATA
GACTTGACGCGTGTCACAATCACACGAGTTAGTATTTTAAAAACCATATTTTATATAATAAGAAGATATATGAACTCATATTG
TATTATAACATTACACAATACTCTAAACAAAATTAGACTTAGTTCATAAGATGCAATAAATGTCAATTACATAAACATTGGTT
ACTTTTGGAAAAAGAGAAGCCACCAATTAATTTTTGTTCCAAGATAATACGCGTACCAATGGTGTATTATATTTTCTTGCAAA
GTATATCCCATCAATTCTGGCAGCCCATACGCGTAGCTGGCCACATCGAGGAATAAGTCTTGGTCCTGCTTTGTATACTTCAA
TGTCGTATGAATACGAATTGTTAGGTCCTTGCCTAAAAATACAATTCCATCTTGTTAAATTGGGTATTTCATCATGGAATCTA
ATGACAAATGGAGTGGCATTGAAGTTTAATTGTTTTACGCCAAGATTGTCATCTTCGGAACGACAATGATATTCGAGAATTTG
ACCAGGAGCGAGTTGATTAAGAATCTCGACTATATTTTTTATGCAAGCTTGATTTAGCCCAAAATACGTAATAGTAATCAAAA
TTACAAATACAGAATGGTTCATTTTTTGGATCTAAACCCTTTATTGAAGTAACAATGACATCAACATGTAGTATTTATAATTA
TTTTGTGTGGAAGACGTTTTTTCCTAAAATTACTAGATAAGCGTTTTATGGTTTTAGCTAAATCGAAAC

Reverse complement

TCONS_00047695 (XLOC_030751 minus strand, At5g36985)

GTTTCGATTTAGCTAAAACCATAAAACGCTTATCTAGTAATTTTAGGAAAAAACGTCTTCCACACAAAATAATTATAAATACT
ACATGTTGATGTCATTGTTACTTCAATAAAGGGTTTAGATCCAAAAA**ATG**AACCATTCTGTATTTGTAATTTTGATTACTATT
ACGTATTTTGGGCTAAATCAAGCTTGCATAAAAAATATAGTCGAGATTCTTAATCAACTCGCTCCTGGTCAAATTCTCGAATA
TCATTGTCGTTCCGAAGATGACAATCTTGGCGTAAAACAATTAAACTTCAATGCCACTCCATTTGTCATTAGATTCCATGATG
AAATACCCAATTTAACAAGATGGAATTGTATTTTTAGGCAAGGACCTAACAATTCGTATTCATACGACATTGAAGTATACAAA
GCAGGACCAAGACTTATTCCTCGATGTGGCCAGCTACGCGTATGGGCTGCCAGAATTGATGGGATATACTTTGCAAGAAAATA
TAATACACCATTGGTACGCGTATTATCTTGGAACAAAAATTAATTGGTGGCTTCTCTTTTTCCAAAAGTAACCAATGTTTATG
TAATTGACATTTATTGCATCTTATGAACTAAGTCTAATTTTGTTTAGAGTATTGTGTAATGTTATAATACAATATGAGTTCAT
ATATCTTCTTATTATATAAAATATGGTTTTTAAAATACTAACTCGTGTGATTGTGACACGCGTCAAGTCTATCGTTCAGATGT
TGACATGTGTCAACAAATATTTACTTTTCTCTTAAATAAATAGGAAAACTAAAAAAAAAAAGAAACATAG
