# Supplementary material for: Characterization of novel pollen-expressed transcripts reveals their potential roles in pollen heat stress response in Arabidopsis thaliana
Source: Plant Reprod. 2021 Jan 18;34(1):61–78. doi: 10.1007/s00497-020-00400-1 (PMC7902599; doi:10.1007/s00497-020-00400-1)
Supplement: Supplementary file 4 — Supplementary file1 (PDF 304 kb) [file 497_2020_400_MOESM4_ESM.pdf]

Doc S4

XLOC_006026 genomic fragment

```
Ch1: 13222206 -
AACGTCTAAATGATCCACTATAAATAACCATCTCATATCGAACAATTACCACAACCACAATTTGCCTCAGTCTAAATGATCTCTCAGAATTTT
TATATAGCAAGGTATTTCTTATGTATAGCTCTAGAGATGTTTTGCTATACAGAATATTTATCAGTGCTTGTGTGACGAATTTAATAGATTgag
tttgtgattttgttgtttgtagAGCTTTATGAGCAAGCTATTGAGTATGAGAAAGGTTCGTTAATCACTTCTAATGGAGCTTTGACGATGCTT
TCTGGTGCTAAGACTGATCGAGAGATAAGCGTGTTGTTAGAGATGCGATTACTGAGGATGAGCTTTGGCGATTACAGAATTTTTATCctctat
tttggtttattgattttgttttgattttgattttgattttaaggGTTTGTCTAATATTGAAATCGATGAACATACTTTCATGGTGGTGAAC
AGAGAAAGAGCTGTTGATTACTTGAATTCCTTGGAAAAGGTTTACCTTATGCCTAAAAGTTAGATTCTTTCCCATCATTCTGGATGCAATATG
GGAAAAGATGGAGATGTTGCCCTCTTCTTTGAACTTTTAGGTATTGCAACTTTTTAAGCCGACGTGAACgctgaaaaaaaagagaaacaccGA
CGTGAACGCAGAGAGAAGATTACTTGCGCAACCATCGAcggaaagaaagagaaacaaaacctttcttcttcttttttttttctatatttgtct
agaaaaaaaaaattaacgttTCTTTTGGATGTAAAGAATCTTTTCTATTTAGATGTTTTGGCacaaaaaacgaagaaaaaaactataattttg
taatttattttattttagtttatgatTAAg     - 13221341
```

In black – exons, Red – introns. Orange – part of the coding sequence of the Q9LQH8 protein that is included in the extended first intron (Orange and red sequence) spliced to give the sequence of TCONS_00009618.
GT and ag – the alternative donor and acceptor splice sites of the extended intron removed in TCONS_00009618.
GT and AG – indicate the correct donor and acceptor sites of intron 1 and 2.

TCONS_00009618 (XLOC_006026)

```
AACGTCTAAATGATCCACTATAAATAACCATCTCATATCGAACAATTACCACAACCACAATTTGCCTCAGTCTAAATGATCTCTCAGAATTTT
TATATAGCAAGAGCTTTATGAGCAAGCTATTGAGTATGAGAAAGGTTCGTTAATCACTTCTAATGGAGCTTTGACGATGCTTTCTGGTGCTAA
GACTGATCGAGAGATAAGCGTGTTGTTAGAGATGCGATTACTGAGGATGAGCTTTGGCGATTACAGAATTTTTATCCTCTATTTTGGTTTATT
GATTTTTGTTTTTGATTTTGATTTTGATTTTAAGGGTTTGTCTAATATTGAAATCGATGAACATACTTTCATGGTGGTGAACAGAGAAAGAGC
TGTTGATTACTTGAATTCCTTGGAAAAGGTTTACCTTATGCCTAAAAGTTAGATTCTTTCCCATCATTCTGGATGCAATATGGGAAAAGATGG
AGATGTTGCCCTCTTCTTTGAACTTTTAGGTATTGCAACTTTTTAAGCCGACGTGAACGCTGAAAAAAAAGAGAAACACCGACGTGAACGCAG
AGAGAAGATTACTTGCGCAACCATCGACGGAAAGAAAGAGAAACAAAACCTTTCTTCTTCTTTTTTTTTTCTATATTTGTCTAGAAAAAAAAA
ATTAACGTTTCTTTTGGATGTAAAGAATCTTTTCTATTTAGATGTTTTGGCACAAAAAACGAAGAAAAAAACTATAATTTTGTAATTTATTTT
ATTTTAGTTTATGATTAAG
```

Black – UTRs, AG – the acceptor site left out from the incorrect splicing of intron 1 that caused a frame shift.
Red sequence – unspliced intron 2. TGA – early stop codon caused by the frame shist.

Pairwise alignment of XLOC_006026 and TCONS_00009618:

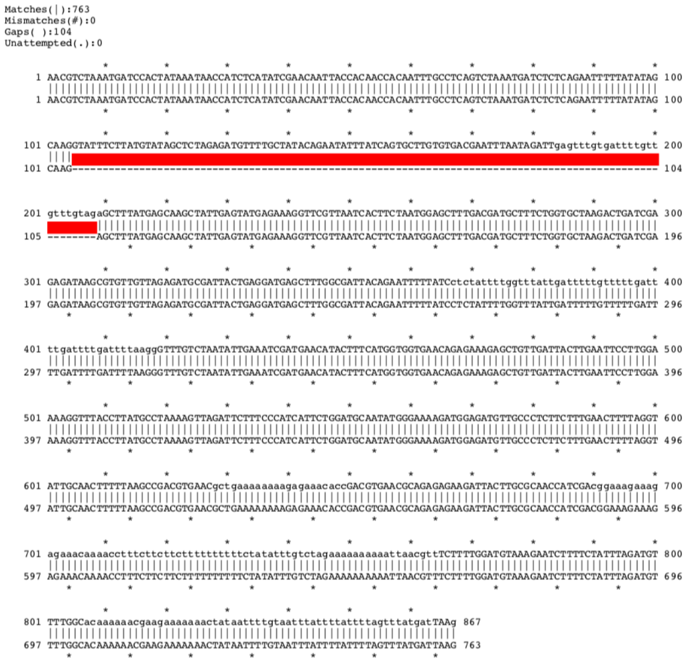

Doc S4

```
Position 107 to 508 with stop at 422-424
```

F15O4.6/Q9LQH8 protein sequence from UnipProt:

```
>tr|Q9LQH8|Q9LQH8_ARATH F15O4.6 OS=Arabidopsis thaliana OX=3702 PE=4
SV=1
MISQNFYIARYFLCIALEMFCYTEYLSVLLYEQAIEYEKGSLITSNGALTMLSGAKTDRE
ISVLLEMRLLRMSFGDYRIFILYFGLLIFVFDFDFDFKGLSNIEIDEHTFMVVNRERAVD
YLNSLEKILSHHSGCNMGKDGDVALFFELLGIATF
```

In red – the polypeptide part of the protein coded by 3’ half of exon 1 (see above in orange) that is excluded from TCONS_00009618
