# Supplementary material for: Characterization of novel pollen-expressed transcripts reveals their potential roles in pollen heat stress response in Arabidopsis thaliana
Source: Plant Reprod. 2021 Jan 18;34(1):61–78. doi: 10.1007/s00497-020-00400-1 (PMC7902599; doi:10.1007/s00497-020-00400-1)

Figure S1. MP_HS RNAseq experiment regime plot


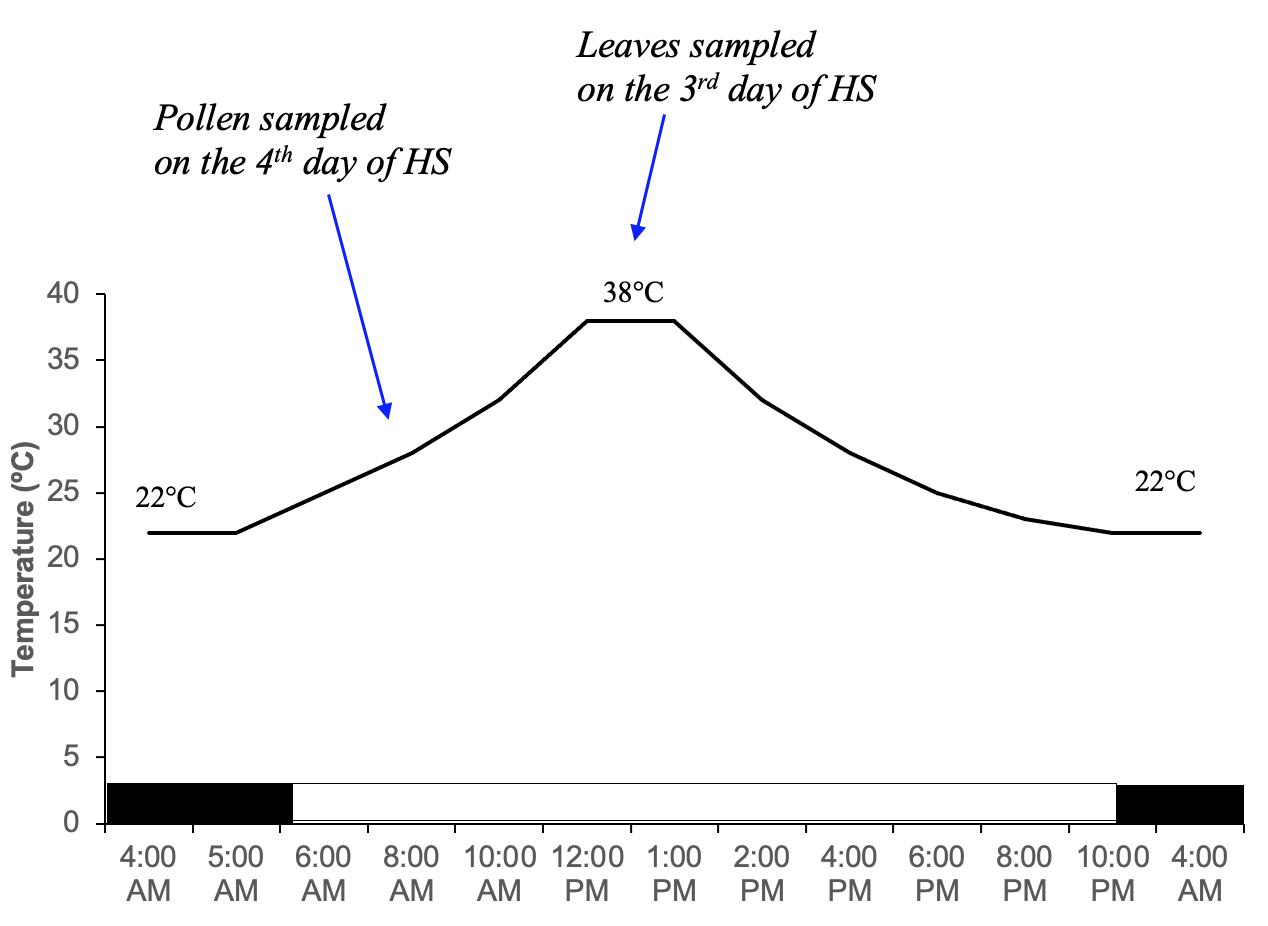


Figure S2: Principal Component Analysis of the MP_HS RNAseq data. PCA plots of pollen (A) and cauline leaves (B).


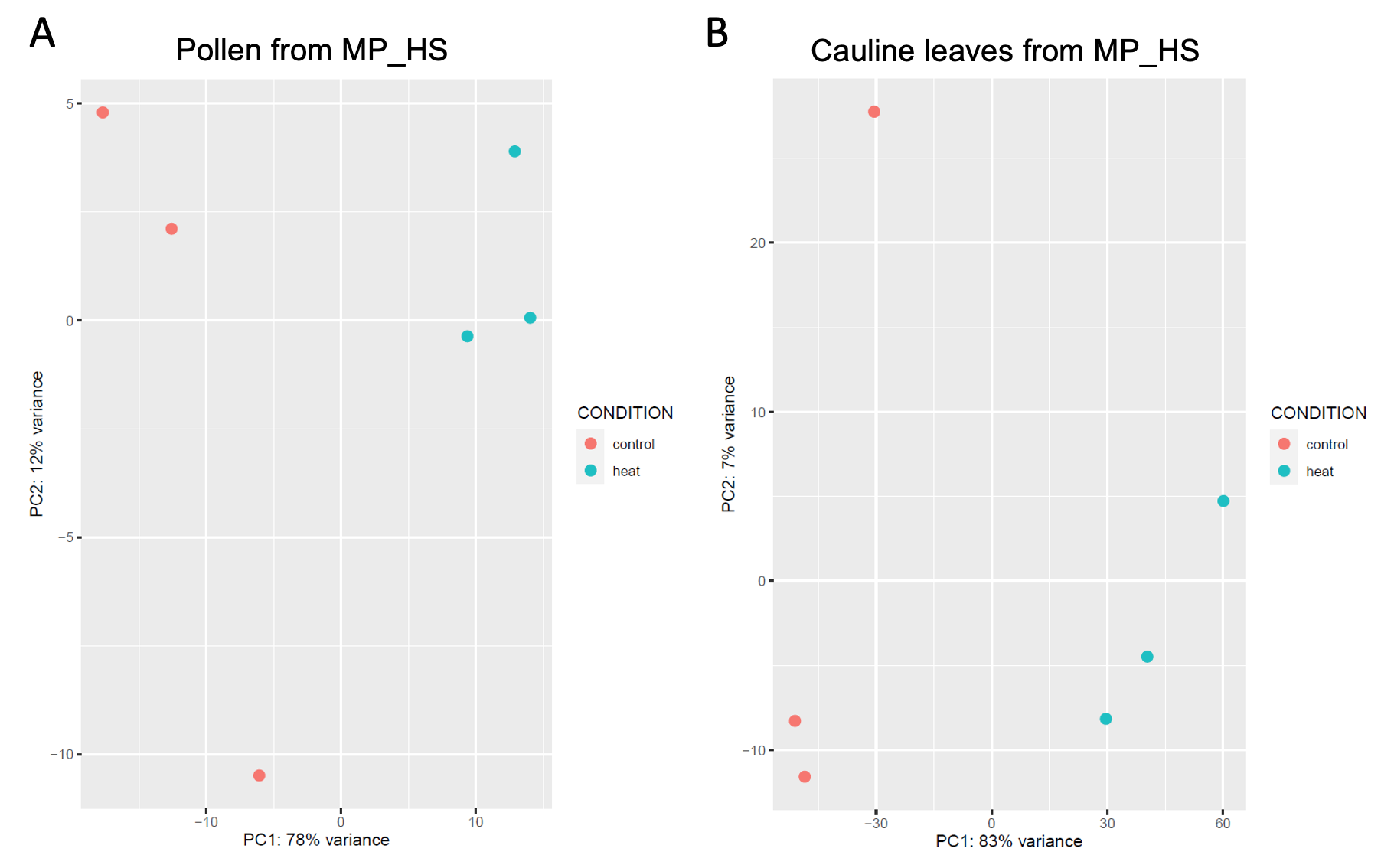


Figure S3. Expression and log2 fold change level of HSPs (A) and HSPs in pollen vs cauline leaves (B).


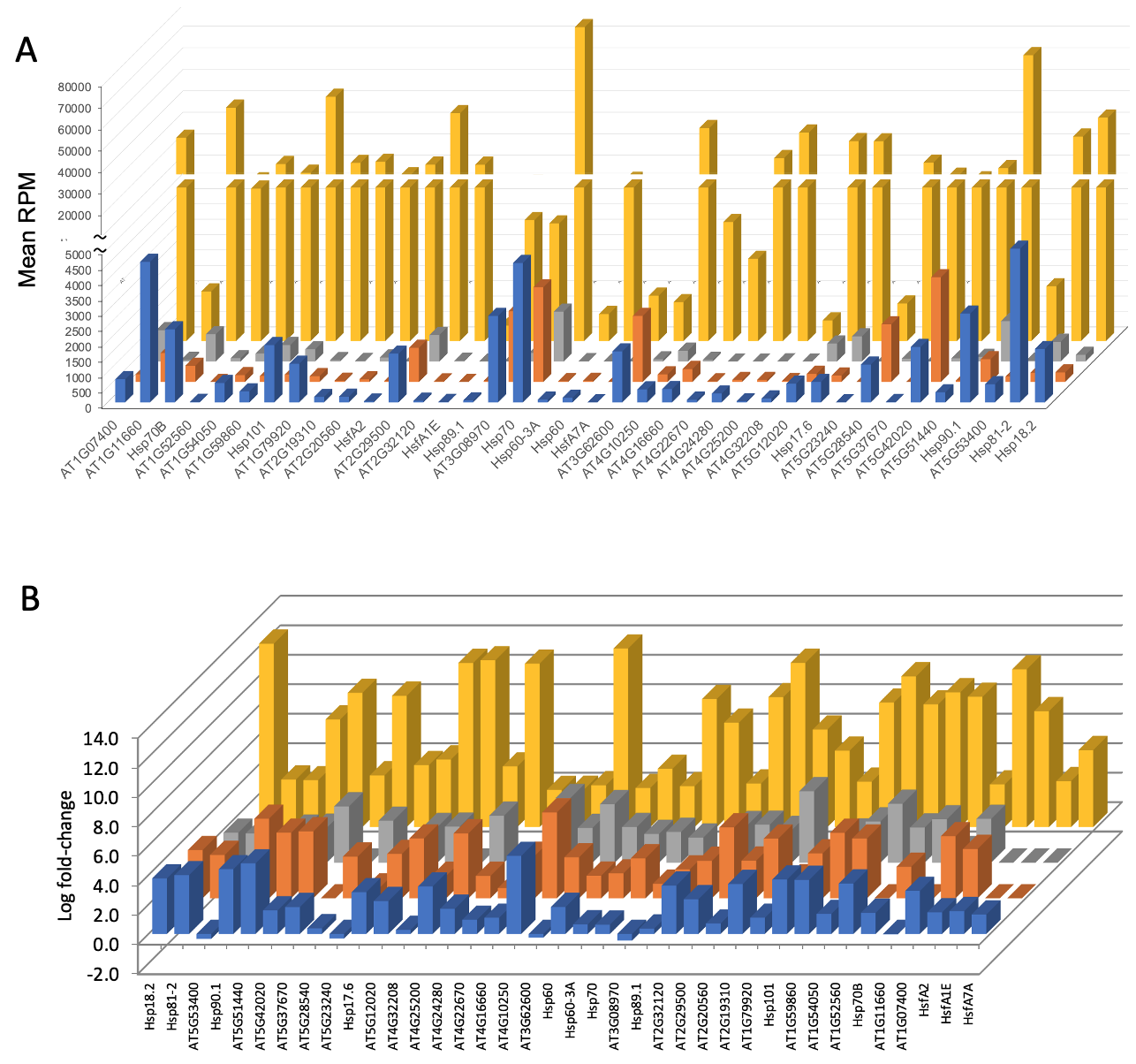


Figure S4. Venn diagrams showing overlap between XLOCs represented in the yellow (A) and red (B) sections in the pie charts in Figure 5D-F.


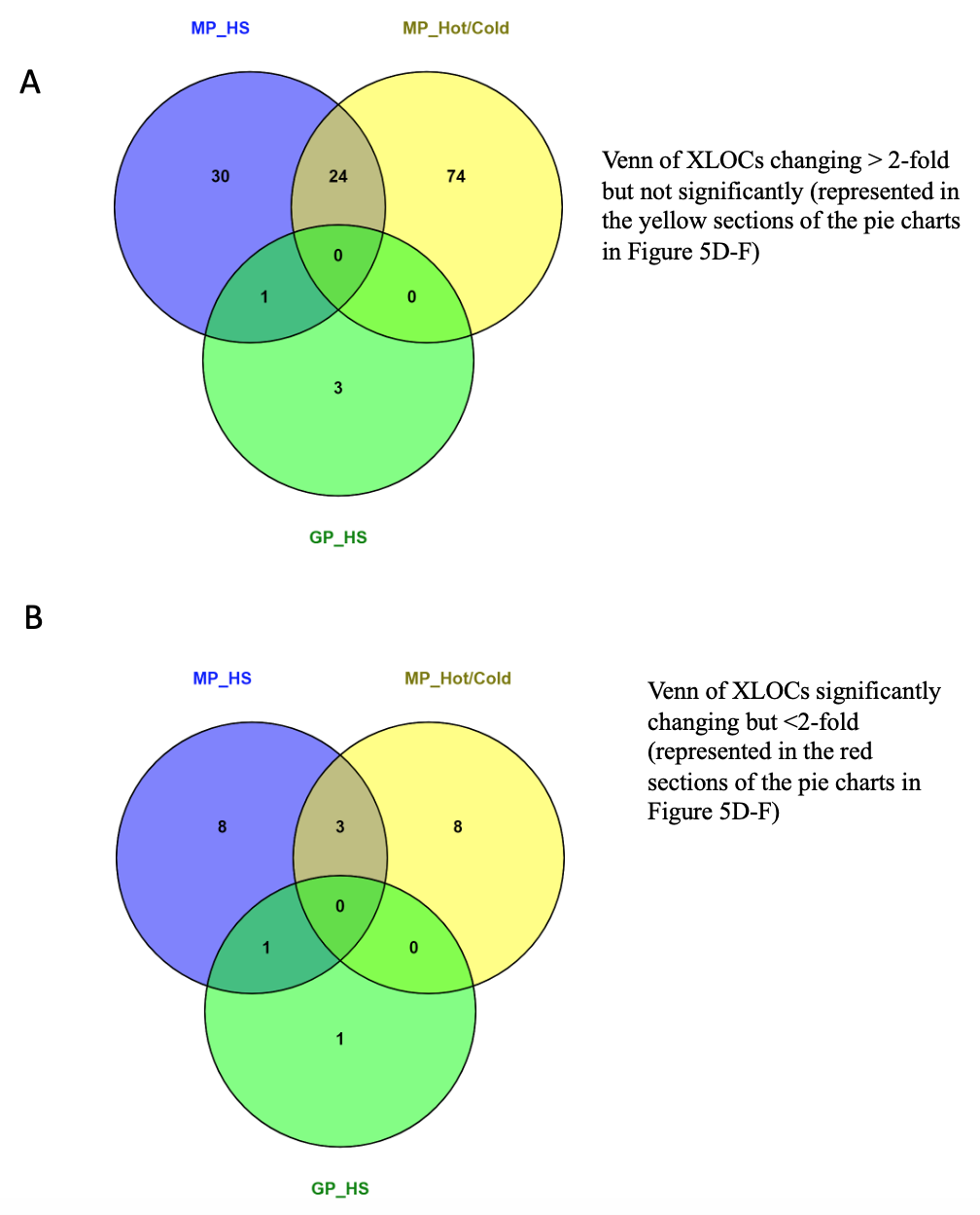


Figure S5: Heatmap comparison of the expression of the 24 MP_HS-specific DEXs, represented in Figure 6B, in the three pollen RNAseq experiments.


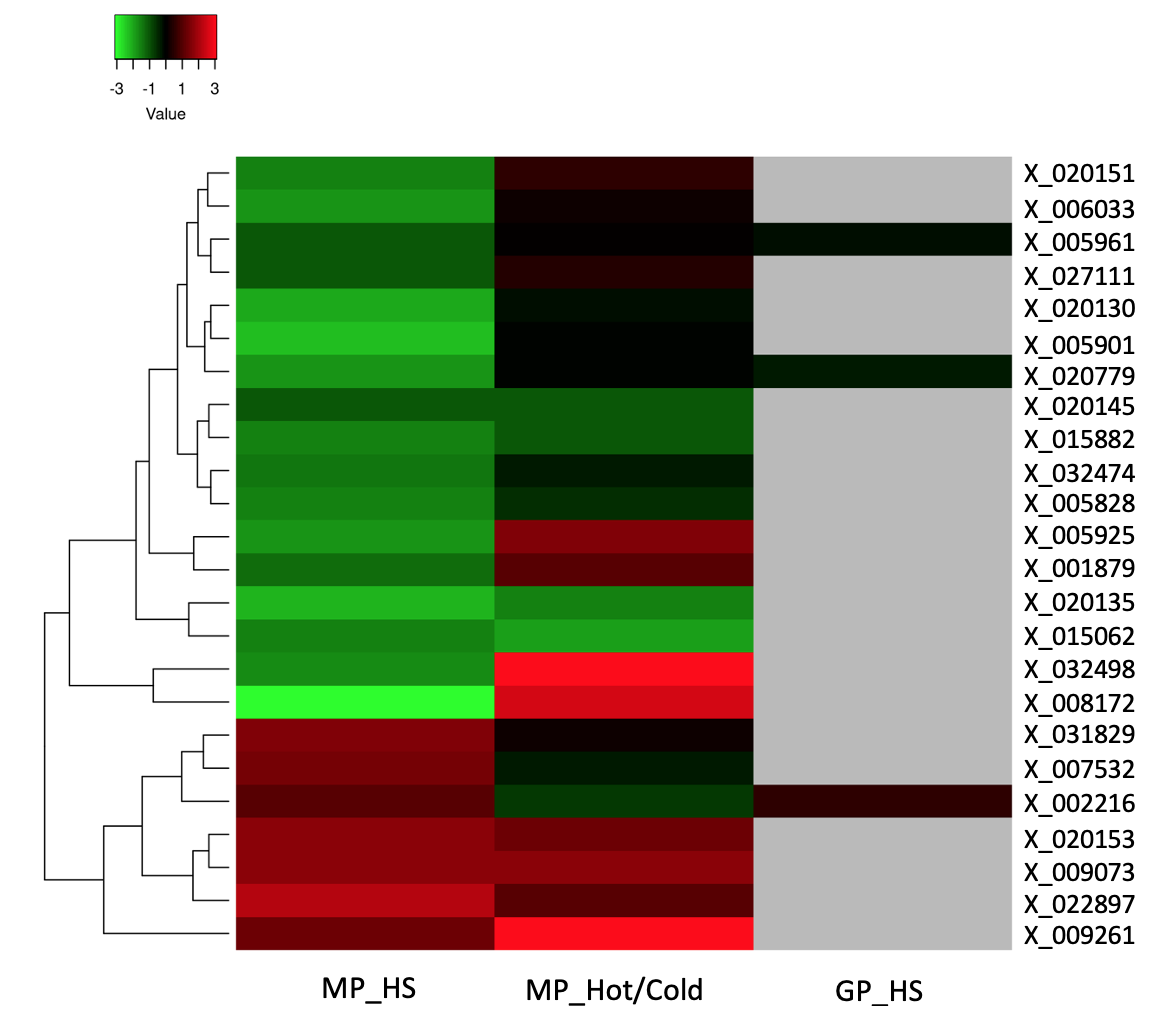


Figure S6: Heatmap comparison of the expression of the 84 MP_Hot/Cold-specific DEXs, represented in Figure 6B, in the three pollen RNAseq experiments.


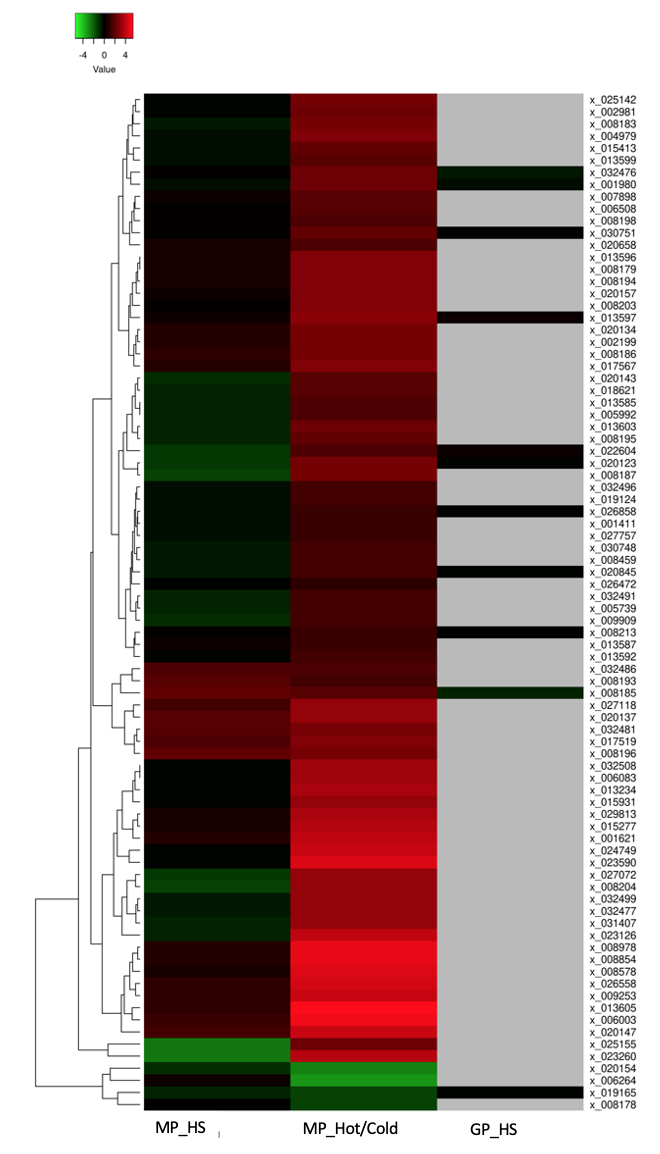


Figure S7: Heatmap comparison of the expression of the 7 GP_HS-specific DEXs, represented in Figure 6B, in the three pollen RNAseq experiments.


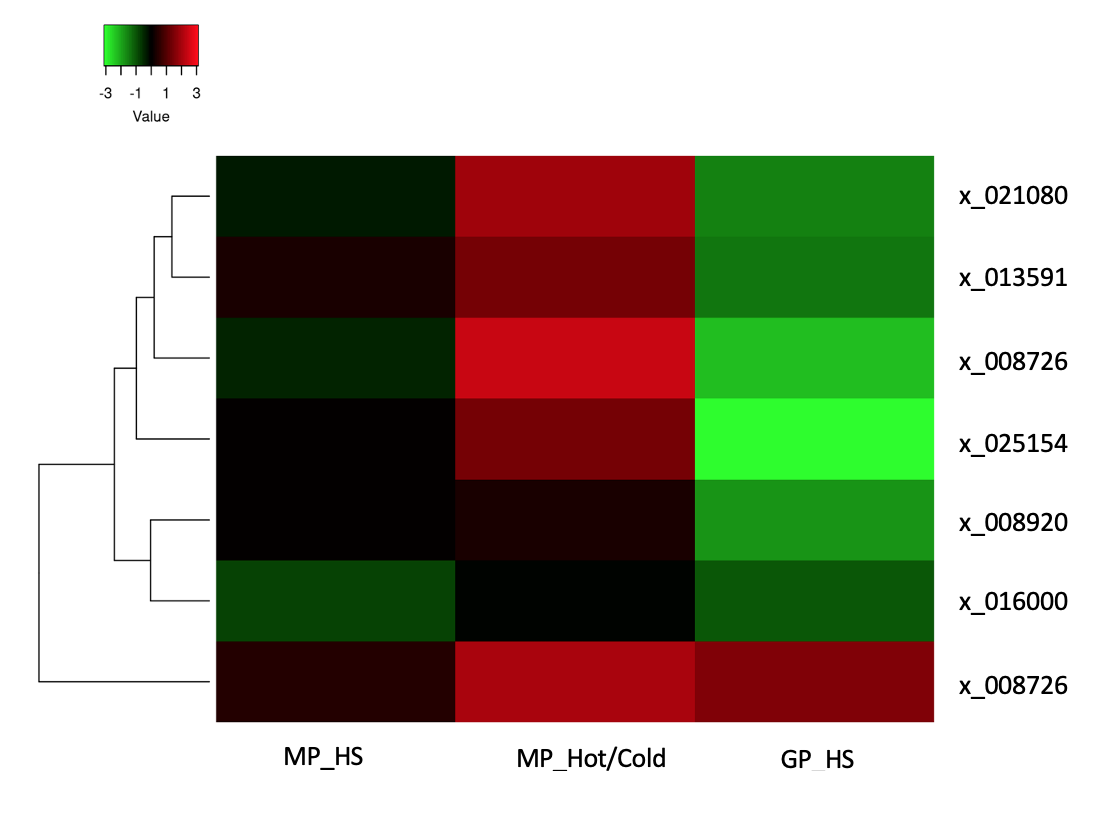


Figure S8: Prediction of miRNA targets among PEGs. A. Pie chart showing the proportion of PEGs predicted to be targets of miRNAs according to the psRNATarget tool. B. Expression of XLOC_032495 and its predicted targeting pri-miR477 in the mature pollen datasets.


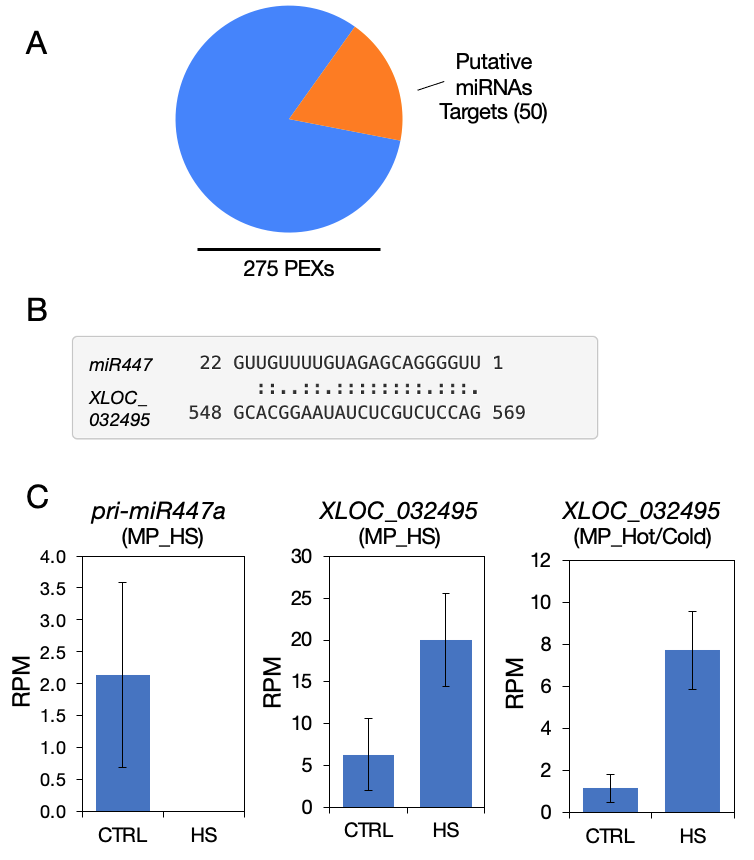

Supplement: Supplementary file 5 — Supplementary file1 (DOCX 1357 kb) [file 497_2020_400_MOESM5_ESM.docx]
